# Evaluation of community-based heat adaptation interventions: a systematic review

**DOI:** 10.1136/bmjph-2024-002332

**Published:** 2025-07-15

**Authors:** Jai K Das, Bhavita Kumari, Abdu R Rahman, Maha Azhar, Sara Khan, Ujala Masood, Deepna Karan, Rahima Yasin, Christopher Burman, Joseph Luke Augustin, Ana Bonell, Zulfiqar A Bhutta

**Affiliations:** 1 Institute for Global Health Department, Aga Khan University, Karachi, Pakistan; 2 Department of Paediatrics and Child Health, Aga Khan University, Karachi, Pakistan; 3 University of Cincinnati, Cincinnati, Ohio, USA; 4 Institute of Business Administration, Karachi, Pakistan; 5 Bielefeld University, Bielefeld, Germany; 6 University College London, London, UK; 7 Centre on Climate Change and Planetary Health, London School of Hygiene & Tropical Medicine, London, UK; 8 Medical Research Council Unit The Gambia at the London School of Hygiene and Tropical Medicine, Banjul, Gambia

**Keywords:** Systematic Review, Community Health, Environmental Medicine, Environmental Exposure

## Abstract

**Introduction:**

Heat adaptation strategies are crucial to minimising the adverse effects of heat on human health. We systematically reviewed published studies till February 2024 to synthesise and evaluate the evidence of the impact of community-based heat adaptation strategies on surface and indoor temperatures and health outcomes.

**Methods:**

Two investigators independently screened relevant databases and extracted data. The review included a total of 141 studies; 124 landscape interventions, out of which 114 focused on green walls/roofs and modified pavements, 21 building modifications including altered construction materials and improved ventilation; and 45 targeted individual-level interventions including heat education and warning systems, heat action plans and modifications in clothing. Meta-analysis was conducted for surface and indoor temperature for each intervention/comparison group for landscape and building modifications, and descriptive analysis was performed on human outcomes for individual-level interventions due to wide variability in reporting of outcomes.

**Results:**

The meta-analysis suggests that green roofs significantly reduced surface temperatures by 10.88°C (95% CI: −15.26°C to –6.50°C) and indoor temperatures by 2.4°C (95% CI: −3.54°C to –1.26°C) compared with conventional roofs. Green walls significantly reduced surface temperatures by 2.39°C (95% CI: −4.03°C to –0.74°C) and indoor temperatures by 2.08°C (95% CI: −3.00°C to –1.16°C) compared with bare walls. Pavements with modified asphalt materials reduced surface temperatures by 5.45°C (95% CI: −6.75°C to –4.15°C) compared with conventional asphalt. Reflective paints significantly reduced surface temperature by 4.55°C (95% CI: −6.74°C to –2.36°C) and indoor temperatures by 1.69°C (95% CI: −3.35°C to –0.02°C) compared with conventional roofs. Most studies examining heat action plans reported a decrease in mortality, and heat education and heat warning systems led to reduced morbidity and mortality and improved heat-related knowledge, attitudes and practices. Studies have reported physical strain alleviation and improvement in body temperature while using modified garments.

**Conclusions:**

With the increasing exposure to extreme heat, contextual evidence will provide valuable insights for effectiveness, acceptance and cost-effectiveness for various interventions.

**WHAT IS ALREADY KNOWN ON THIS TOPIC**

- There is existing evidence on heat adaptation strategies, but most have focused on a single modification without rigorous statistical analysis and have included simulations, modelling and experimental settings.

**WHAT THIS STUDY ADDS**

- This systematic review synthesises the existing evidence through robust statistical measures and provides evidence on what community-based strategies can reduce surface and indoor temperatures and impact human health.

**HOW THIS STUDY MIGHT AFFECT RESEARCH, PRACTICE OR POLICY**

- Integrating these strategies into planning and regulations can help mitigate the adverse effects of rising temperatures and improve thermal comfort and health.

## Introduction

 The climate crisis is rapidly intensifying, causing an increase in the frequency and intensity of heatwaves (Three consecutive days or more when the max. temperature each day is in top 10% of local 15-day average^1^), at both global and regional levels.^2^,^4^ The mean global temperature has increased by at least 1.1°C since the late 1800s.^5^ Heatwaves have been progressively more severe since the 1950s, with unprecedented incidents frequently observed in many different parts of the world,^2^ including scorching temperatures above 48°C in South Asia, historic droughts in Africa and record-breaking temperatures in Europe negatively affecting human health and nutrition.^1^ Projections indicate that by the end of this century, the average global surface temperature will surpass 2.7°C above preindustrial temperatures, if the current policies persist.^6^

Heatwaves have emerged as a pressing environmental challenge, profoundly impacting various aspects of human life and the natural world.^7^ Public health is particularly affected by heatwaves, as prolonged exposure to extreme heat can lead to heat-related illnesses and fatalities, especially among vulnerable populations like the elderly, children and individuals with pre-existing health conditions.^8 9^ Globally, heat is responsible for almost half a million deaths each year,^1^ and heatwaves can also exacerbate air pollution, increasing harmful particulate concentrations and further compromising respiratory health, particularly in urban areas.^10^ The resulting adverse health outcomes put significant strains on healthcare systems, necessitating urgent interventions to protect communities’ well-being.

Urbanisation stands as a prominent challenge in the modern world. The swift surge in urbanisation, particularly in the developed world, has led to the substantial conversion of natural vegetation into concrete structures, often characterised by low-reflectivity surfaces. This transformation gives rise to a range of grave environmental concerns, foremost among them being the urban heat island effect.^11^ Escalating temperatures also trigger heat stress in crops and livestock, leading to reduced yields and food insecurity, posing challenges to global food systems.^12^ Given the multifaceted consequences of heatwaves on public health, agriculture, ecology and the environment, it is crucial to comprehensively assess the targeted adaptation and mitigation efforts, enhancing resilience and minimising future heatwave impacts.

Urgent measures are required to develop evidence-based cooling strategies for managing health risks linked to the inevitable course of climate change during heatwaves and hot weather. Implementing approaches at both the urban and building levels, such as incorporating blue and green spaces into landscapes and using alternative building materials and natural ventilation, can significantly enhance society’s ability to adapt to heatwaves and hot conditions.^13^ Other strategies targeting individuals’ adaptation to heat may include heat warning systems with increased awareness, and modification of clothing and work schedules. Effective cooling solutions can also be adopted at the individual level, even in resource-limited settings, to alleviate physiological heat strain.^13^ To ensure optimal health protection, robust heat action plans (HAPs) grounded in evidence, well-communicated and informed by real-time surveillance are essential.

Multiple efforts to synthesise heat adaptation evidence have been attempted, most of which have focused on a single modification,^14^,^19^ and reviews have not attempted a stringent statistical analysis^13 20 21^ and have also included simulations, modelling and experimental settings.^14 22 23^ The evidence on heat adaptation strategies is expanding; hence, there is a need to holistically evaluate the impact of the heat adaptation strategies systematically. This systematic review comprehensively summarises the evidence of the various heat adaptation strategies categorised as (1) landscape modifications, (2) modifications to building structures and (3) individual-level adaptation strategies and evaluates its impact on temperature and human health.

## Methods

### Inclusion criteria

We included studies which assessed interventions to mitigate or adapt to heat stress by measuring surface and indoor temperatures compared with standard practices in both rural and urban settings in all countries. The review included randomised controlled trials, quasi-experimental trials and small-scale experiments in the natural environment.^13^

### Exclusion criteria

We excluded studies which were computer-based simulation or modelling or analysed hypothetical situations, and only actual interventions conducted in real life settings were included. Cross-sectional studies evaluating the effects of existing structures were excluded. Studies conducted in heat chambers or where heat stress was higher than natural environments were excluded, for example, brick-kilns or in the mining industry.

### Interventions and outcomes

We have organised heat adaptation interventions as described by Jay *et al*^13^ into three broad categories—landscape, building and individual-level (online supplemental panel 1). The two temperature-related outcomes included were surface and indoor room temperatures, both being measured in centigrade. Outcomes related to human health and behaviours were also assessed but could not be meta-analysed due to variance in interventions, definitions and measurements.

### Search strategy and screening

We developed a search strategy for each of the following selected electronic databases: PubMed, CINAHL, Cochrane, Lilac, Scopus, ClinicalTrials.gov and grey literature to identify studies examining the effects of heat mitigation and adaptation strategies on temperature and human health (see online supplemental annex 1). Only articles available as full text in English were included, and no restrictions were applied on year of publication, region of study settings or age of participants. Medical subject heading (MeSH) and keywords used for heat exposure included: heat stress, climate change, hot temperature, urban heat, temperature rise, heat wave, heat-related illness, thermal stress, extreme heat. Keywords used for interventions included: ergonomics, facility, design, construction, ventilation, heat, health, misting, healthcare preparation, green planting, fans, ice, refrigerator, capacity building, reflective paint, albedo, trees, ice-water, community mobilisation, clothing, early warnings, cooling vest, heat monitoring, education campaign, cool coatings. The last database search was conducted on 29 February 2024. Further, existing systematic reviews and included studies were cross-referenced to capture missing studies.

### Data extraction and analysis

Screening and selection of articles was conducted on Covidence software, independently and in duplicates (BK, SK and MA), guided by the pre-established eligibility criteria. The studies which were considered eligible during the title and abstract screening phase were selected for full-text review. Conflicts were resolved through discussion, and a third reviewer was consulted in case of persistent conflict.

Data from each study was extracted by two reviewers (BK, SK and MA), including the study identifiers, study context, design and limitations and intervention and outcome details into a standardised extraction form. Most studies examining landscape and building modifications present hourly surface and indoor temperature readings in visual presentations (eg, line graphs) for the duration of the experiments; these readings were manually extracted from the graphs into tabular form. Mean temperatures for a day and SD were calculated using MS Excel from these hourly values and meta-analysis conducted. For studies examining individual-level adaptations, a wide range of outcomes were studied including morbidity and mortality, body temperature, knowledge, attitudes and practice (KAP), thermal comfort and heat strain alleviation and vital signs.

Multiple meta-analyses were conducted for the various groups of similar interventions comparing them with common control groups for each set of analysis, as described in online supplemental panel 2. The landscape and building modifications interventions were further subgrouped by intervention subtypes; meta-analyses were conducted for each subtype if there were more than one study which compared the intervention with the same control. The pooled mean difference (MD) and 95% CI were reported using random effects models, as surface and indoor temperatures are continuous outcomes. Meta-analysis was performed for all comparisons, using Hedges’ g for calculating MD through ‘metacont’ function from ‘meta package’ in RStudio.^24 25^

For studies examining individual-level adaptation strategies, descriptive analysis was conducted, as a meta-analysis was not possible, due to the wide range of interventions, study designs and outcome definitions and reporting.

Risk of bias was assessed by two independent reviewers using the Cochrane Risk of Bias tool for all randomised controlled trials (RCTs) and Risk of Bias in Non-randomised Studies-Intervention tool for quasi-experimental studies. A sensitivity analysis was performed to evaluate the robustness of the review’s findings by examining the effect of excluding outlier estimates in a meta-analysis and studies with high risk of bias.

## Results

**Figure 1:**
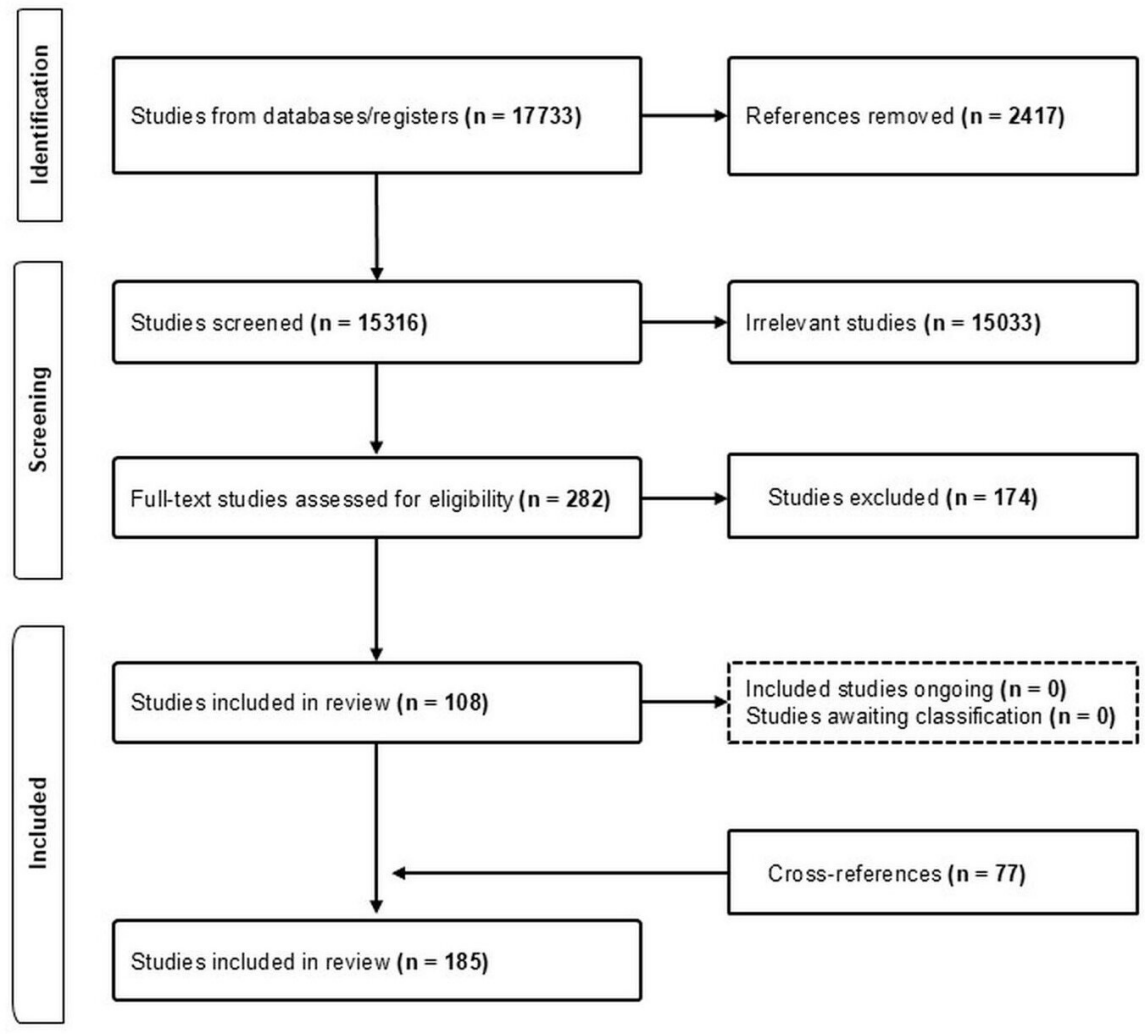
Search strategy flow diagram.

**Figure 2:**
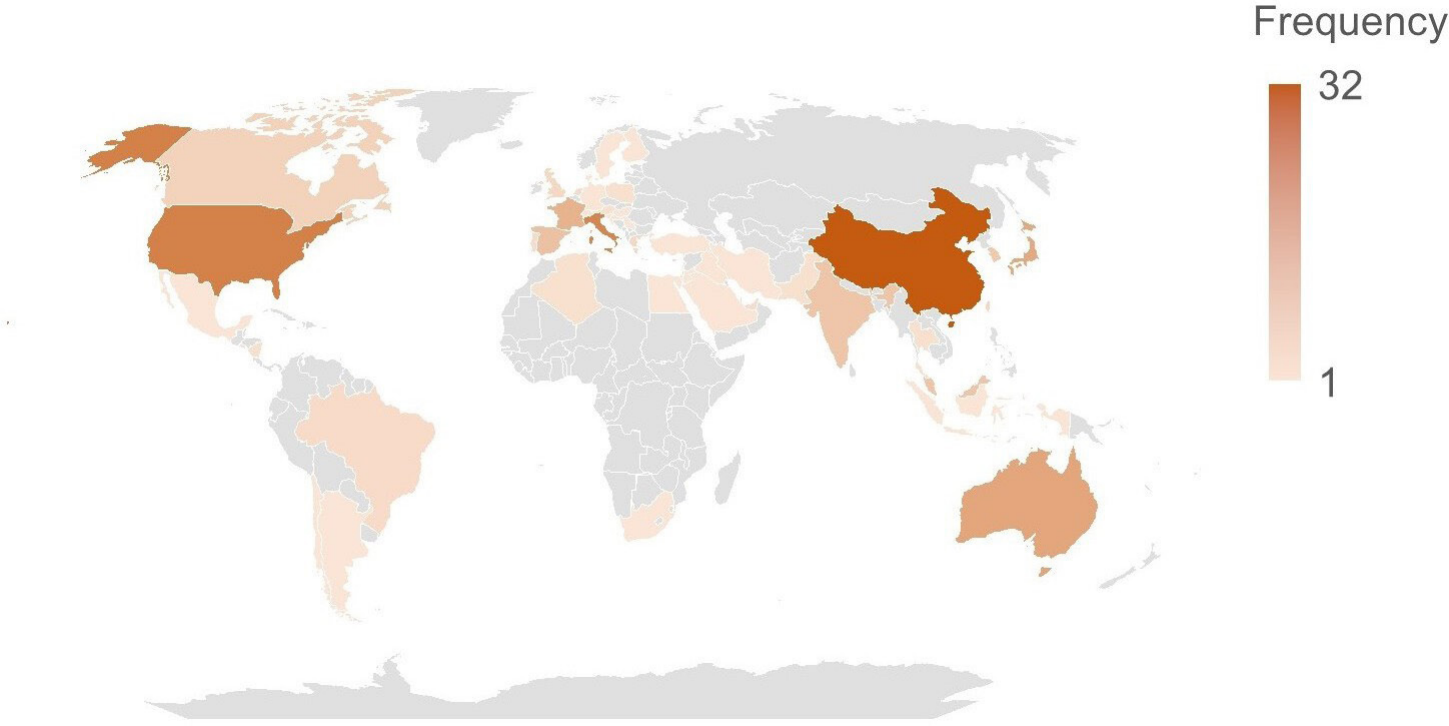
Geographical distribution of the heat adaptation interventions included in the review.

The search identified a total of 17 733 titles and abstracts, and we reviewed full text for 282 papers and included 108 in our review, and identified 77 studies from cross-referencing and grey literature, leading to a total of 185 studies (see figure 1, online supplemental annex 2) and the geographical distribution of the included studies is illustrated in figure 2. Some included studies report more than one intervention. The interventions are categorised into the following sections—landscape (124 studies), building (21 studies) and individual-level (45 studies) adaptations. Online supplemental panel 2 describes the various intervention and comparison groups included in the meta-analyses. The complete set of forest plots is given in onlinesupplemental annex 3  4.

### Landscape interventions

**Figure 3:**
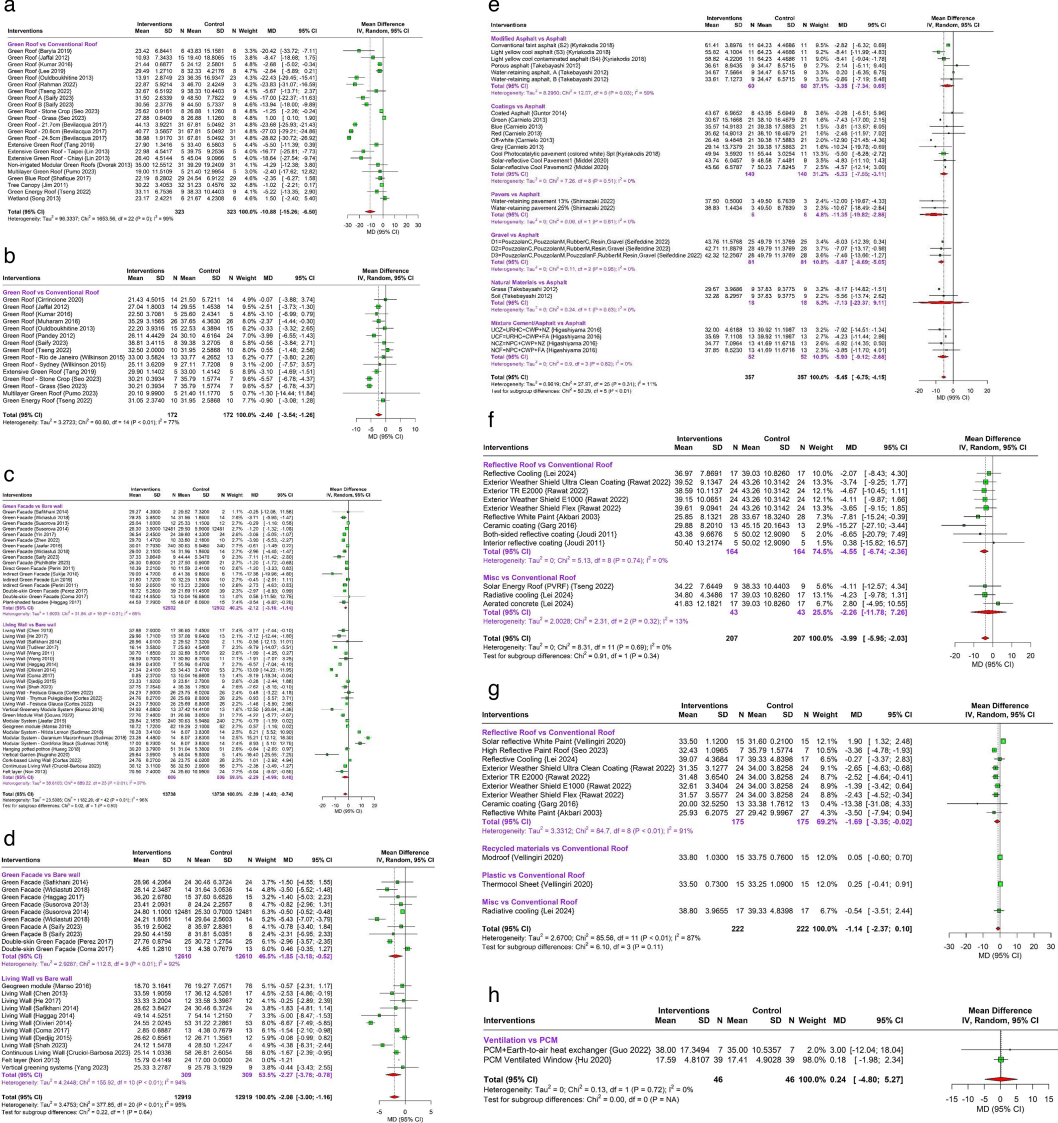
Impact of landscape heat adaptation interventions and modifications in building structures on surface and indoor temperatures. (a) Impact of green roofs on surface temperature compared to conventional roofs. (b) Impact of green roofs on indoor temperature compared to conventional roofs. (c) Impact of green walls on surface temperature compared to bare walls. (d) Impact of green walls on indoor temperature compared to bare walls. (e) Impact of pavement modifications on surface temperature compared with conventional asphalt. (f) Impact of modifications in building roofs on surface temperature compared to conventional roofs. (g) Impact of modifications in building roofs on indoor temperature compared to conventional roofs. (h) Impact of modifications in building ventilation on surface temperature compared to PCM. PCM, phase change material.

The landscape interventions in 124 studies included green walls and roofs and modified pavements and assessed its impact on surface and indoor temperatures (tables1  2, figure 3).

**Table 1:** Summary of findings for the effect of landscape heat adaptation interventions on surface temperature

# of studies	Design	Consistency	Generalisability to population of interest	Generalisability to intervention of interest	# of estimates	MD (95% CI)	Comments
Surface temperature							
Green roofs compared with bare concrete roof							
17	Experimental^26^,^4046 51^	Considerable heterogeneity, I^2^=92%	13 studies were conducted in HICs, 4 were conducted in LMICs.Ambient temp range: 14°C–35°C	The study compared various green roofs to conventional roofs. Results pooled for different types of interventions.	23	−10.88°C(−15.26°C, to 6.50°C)	Interventions included green roofs (14), extensive green roofs (3), non-irrigated modular green roof (1), multilayer green roof (1), green blue roof (1), tree canopy (1), green energy roof (1), wetland (1).
Green roofs compared with bare substrate							
2	Experimental^48 49^	Moderate heterogeneity, I^2^=54%	2 studies were conducted in HICsAmbient temp range: 13°C–24°C	The study compared various green roofs to bare substrate roofs. Results pooled for different types of interventions.	39	−1.16°C(−1.38°C to 0.94°C)	Interventions included green roofs (5), modular green roofs (34).
Green roofs compared with wooden roof							
2	Experimental^44 45^	No heterogeneity	One study was conducted in HIC and in LMIC each.Ambient temp: 36.1°C	The study compared various green roofs to wood roofs. Results pooled for different types of interventions.	3	−0.59°C(−1.98°C to 0.79°C)	Interventions included green roof (1), sand (1), pellet (1).
Green roofs compared with painted roof							
4	Experimental^41^,^43130^	No heterogeneity	3 studies were conducted in HICs, 1 was conducted in LMIC.Ambient temp range: 33°C–45°C	The study compared various green roofs to miscellaneous roofs. Results pooled for different types of interventions.	5	−3.28°C(−7.18°C to 0.62°C)	Interventions included green roofs (4), extensive vegetated roofs (1). Comparison groups include black roof (1), white roof (1), white gravel roof (1), green paint (2).
Green roofs compared with non-reflective (EPDM) roof membrane							
1	Experimental^47^	No heterogeneity	The study was conducted in HICAmbient temp: 29.7°C	The study compared various green roofs to miscellaneous roofs. Results pooled for different types of interventions.	9	−1.77°C(−2.27°C to to 1.28°C)	Interventions included modular green roofs (9).
Green walls compared with bare walls							
33	Experimental (30), Quasi-experimental (1)^3358^,^86 94^	Considerable heterogeneity, I^2^=87%	26 studies were conducted in HICs, 7 were conducted in LMICsAmbient temp range: 15°C–35°C	The study compared various green roofs to bare roofs. Results pooled for different types of interventions.	43	−2.39°C(−4.03°C to to 0.74°C)	Interventions included green facades (18), living walls (26)
Green walls compared with bricked walls							
7	Experimental^87^,^9092 93 95^	Considerable heterogeneity, I^2^=99%	6 studies were conducted in HICs, 1 was conducted in LMIC.Ambient temp range: 10°C–30°C	The study compared various green roofs to miscellaneous roofs. Results pooled for different types of interventions.	11	−2.12°C(−2.87°C to 1.37°C)	Interventions included green facades (8), living walls (3)
Green walls compared with galvanised plate							
1	Experimental^91^	No heterogeneity	The study was conducted in HICAmbient temp: 22.6°C	The study compared various green facades to galvanised plates. Results pooled for different types of interventions.	5	−0.47°C(−1.03°C to 0.08°C)	Interventions included green facades (5)
Modified pavement compared with conventional concrete							
9	Experimental^99 101 102 105 106 108 109 112 113^	Considerable heterogeneity, I^2^=76%	All studies were conducted in HICsAmbient temp range: 27°C–34°C	All studies compared interventions to conventional concrete. Results pooled for different types of interventions.	29	−1.14°C(−2.91°C to 0.63°C)	Studies included modified concrete (4), modified pavers (3), solar-reflective coatings (1), metals (2), natural materials (2)
Modified pavement compared with conventional asphalt							
8	Experimental^100104 107 109^,^112 114 115^	Insignificant heterogeneity, I^2^=10%	8 studies were conducted in HICs.Ambient temp range: 29°C–34°C	All studies compared interventions to conventional asphalt. Results pooled for different types of interventions.	26	−5.45°C(−6.75°C to 4.15°C)	Studies included modified asphalts (2), solar-reflective coatings (4), modified pavers (1), gravel (2), natural materials (1), cement mixtures (1)
Modified pavement compared with white gravel							
1	Experimental^115^	No heterogeneity	Study was conducted in HICAmbient temp: 19.7°C	The study compared variation in gravel thickness to gravel 8–22.4 mm. Results pooled for different types of interventions.	3	−4.46°C(−10.61°C to 1.69°C)	One study comparing variation in gravel thickness
Modified pavement compared with black brick							
1	Experimental^103^	No heterogeneity	Study was conducted in HIC	The study compared variously shaded bricks to black bricks. Results pooled for different types of interventions.	4	−4.50°C(−8.3°C to 0.63°C)	One study comparing shade variation in black bricks
Modified pavement compared with grass							
1	Experimental^112^	No heterogeneity	Study was conducted in HICAmbient temp: 28.9°C	The study compared various modified concretes and asphalts to grass. Results pooled for different types of interventions.	8	4.96°C(4.11°C to 5.81°C)	One study included modified concretes and asphalts
Modified pavement compared with soil							
1	Experimental^112^	No heterogeneity	Study was conducted in HICAmbient temp: 28.9°C	The study compared various modified concretes and asphalts to soil. Results pooled for different types of interventions.	8	2.39°C(1.53°C to 3.25°C)	One study included modified concretes and asphalts
Landscape shades compared with no shade							
2	Experimental^44 93^	No heterogeneity	Both studies were conducted in HICsAmbient temp range: 18°C–36°C	The study compared landscape shading to no shading. Results pooled for different types of interventions.	2	−0.64°C(−2.15, 0.86)	Studies included shaded sail (1) and shaded roof (1)
Landscape irrigation compared with no irrigation							
3	Experimental^117 124 125^	Considerable heterogeneity, I^2^=87%	All studies were conducted in HICsAmbient temp range: 26°C–30°C	The study compared water irrigation systems. Results pooled for different types of interventions.	3	−2.79°C(−22.72, 17.15)	Studies included water irrigation systems (3)

EPDM, Ethylene Propylene Diene Monomer; HICs, high-income countries; LMICs, low-income or middle-income counties; MD, mean difference.

**Table 2:** Summary of findings for the effect of landscape heat adaptation interventions on indoor temperature

# of studies	Design	Consistency	Generalisability to population of interest	Generalisability to intervention of interest	# of estimates	MD (95% CI)	Comments
Indoor temperature							
Green roofs compared with bare concrete roof roofs							
12	Experimental^2829 31 33 34 36 46 51^,^55^	Considerable heterogeneity, I^2^=68%	9 studies were conducted in HICs, 3 were conducted in LMICsAmbient temp range:12°C–35°C	The study compared various green roofs to conventional roofs. Results pooled for different types of interventions.	15	−2.40°C(−3.54°C to 1.26°C)	Interventions included green roofs (13), extensive green roof (1), green energy roof (1)
Green roofs compared with metal roofs							
1	Experimental^56^	No heterogeneity, I^2^=0%	Study was conducted in HICAmbient temp range:28°C–29°C	The study compared various green roofs to metal roofs. Results pooled for different types of interventions.	2	−0.69°C(−1.71°C to 0.32°C)	Interventions included extensive green roof (1), soil substrate (1)
Green roofs compared with wooden roofs							
2	Experimental^45 57^	No heterogeneity, I^2^=1%	Both studies were conducted in HICs	The study compared various green roofs to wood roofs. Results pooled for different types of interventions.	5	−0.83°C(−1.32°C to 0.34°C)	Interventions included extensive green roof (3), soil substrate (1), pellet substrate (1)
Green roofs compared with painted roofs							
2	Experimental^41 50^	No heterogeneity, I^2^=0%	Both studies were conducted in HICsAmbient temp: 33°C	The study compared various green roofs to miscellaneous roofs. Results pooled for different types of interventions.	3	−3.21°C(−7.50°C to 1.08°C)	Interventions included green roofs (3). Comparison groups include black roof (1), green paint (2)
Green walls compared with bare walls							
17	Experimental^3358^,^61 68^	Considerable heterogeneity, I^2^=87%	16 studies were conducted in HICs, 1 was conducted in LMICAmbient temp range:20°C–35°C	The study compared various green roofs to miscellaneous roofs. Results pooled for different types of interventions.	22	−2.08°C(−3.00°C to 1.16°C)	Interventions included green facades (10), living walls (12)
Green walls compared with bricked walls							
1	Experimental^87^	Moderate heterogeneity, I^2^=51%	Study was conducted in HICAmbient temp: 30.9°C	The study compared various green roofs to miscellaneous roofs. Results pooled for different types of interventions.	2	−1.79°C(−9.06°C to 5.48°C)	Interventions included green facades (2)
Green walls compared with double glazed façade							
1	Experimental^98^	Considerable heterogeneity, I^2^=89%	Study was conducted in HICAmbient temp range:30°C–40°C	The study compared various double green glazed facades to double glazed facades. Results pooled for different types of interventions.	4	−0.75°C(−0.88°C to 0.62°C)	Interventions included double green glazed facade (4)
Houses with green roofs and walls compared with bare houses							
2	Experimental^33 55^	Considerable heterogeneity, I^2^=91%	One study was conducted in HIC and one in LMICAmbient temp range:22°C–31°C	The study compared various green houses to bare houses. Results pooled for different types of interventions.	3	−3.16°C(−11.19°C to 4.86°C)	Interventions included green houses with green walls and roofs (3)
Landscape shades compared with no shade							
6	Experimental^44118^,^122^	No heterogeneity, I^2^=74%	All studies were conducted in HICsAmbient temp range:27°C–38°C	The study compared landscape shading to no shading. Results pooled for different types of interventions.	12	−0.10°C(−0.43°C to 0.23°C)	Interventions included shaded sail (7), shaded structure (3) and other sheets (2)
Landscape water irrigation compared with no irrigation							
2	Experimental^123 126^	Low heterogeneity, I^2^=37%	Both studies were conducted in HICsAmbient temp range:26°C–31°C	The study compared water irrigation systems. Results pooled for different types of interventions.	2	−1.99°C(−26.75°C to 22.78°C)	Interventions included water irrigation systems (2)

HIC, high-income country; LMIC, low-income or middle-income country; MD, mean difference.

#### Green roofs

A total of 44 studies with green roofing interventions were included in the meta-analysis for surface and indoor temperature^26^,^51^; separate meta-analyses were conducted for different sets of interventions comparing each intervention with various controls, including conventional, wood, metal, and painted roofs, bare substrate, and non-reflective Ethylene Propylene Diene Monomer (EPDM) roof membrane.

##### Surface temperature

Meta-analysis suggests that green roofs significantly decrease the surface temperature by 10.88°C compared with bare concrete roofs (95% CI: −15.26°C to –6.50°C; 17 studies)^26^,^4051^ (see figure 3a), 1.16°C compared with bare substrate (95% CI: −1.38°C to –0.94°C; two studies),^48 49^ and 1.17°C compared with non-reflective EPDM roof membrane (95% CI: −2.27°C to –1.28°C; one study).^47^ However, there was no significant difference when green roofs were compared with wooden roofs (MD: −0.59°C, 95% CI: −1.98°C to 0.79°C; two studies),^44 45^ and painted roofs (either black, green or white) (MD: −3.28°C, 95% CI: −7.18°C to 0.62°C; 4 studies).^41^,^4350^

##### Indoor temperature

A total of 17 studies analysed indoor temperatures for green roofing interventions.^2829 31 33 34 36 41 45 46 50^,^57^ Meta-analysis suggests that green roofs significantly decrease indoor temperature by 2.40°C compared with conventional roofs (95% CI: −3.54°C to –1.26°C, 15 studies)^2829 31 33 34 36 46 51^,^55^ (see figure 3b) and 0.83°C compared with wood roofs (95% CI: −1.32°C to –0.34°C, 2 studies).^45 57^ However, there was no significant difference when green roofs were compared with metal roofs (MD: −0.69°C, 95% CI: −1.71°C to 0.32°C; one study),^56^ and painted roofs (MD: −3.21°C, 95% CI: −7.50°C to 1.08°C; 2 studies).^41^,^4350^

### Green walls

A total of 51 studies with green wall interventions were included in the meta-analysis for surface and indoor temperature;^3358^,^96^ separate meta-analyses were conducted for different interventions comparing each intervention with various controls, including bare walls, bricked walls, galvanised plate and double-glazed facades. Within each comparison, green walls were further categorised as green facades and living walls.

#### Surface temperature

Meta-analysis suggest that green walls significantly decrease the surface temperature by 2.39°C compared with bare walls (95% CI: −4.03°C to –0.74°C; 33 studies)^3358^,^86 94^ (see figure 3c), and 2.12°C compared with bricked walls (95% CI: −2.87°C to –1.37°C; 7 studies).^87^,^9092 93 95^ The subgroup analyses suggest that green facades significantly decrease the surface temperature by 2.12°C compared with bare walls (95% CI: −3.10°C to –1.14°C; 15 studies)^3358^,^70 95^ and 1.87°C compared with bricked walls (95% CI: −2.87°C to –0.87°C; 8 studies).^87^,^8992 93 95^ Living walls significantly decrease the surface temperature by 2.87°C compared with bricked walls (95% CI: −3.63 to –2.11; 2 studies)^90 92^ However, the subgroup analysis for living walls was not significant compared with bare walls (MD: −2.29°C; 95% CI: −4.99°C to –0.40°C; 21 studies).^5864 69 71^,^86 94 96^ Meta-analysis of green walls did not have a statistically significant difference in surface temperature when compared with galvanised plate (MD: −0.47°C; 95% CI: −1.03°C to 0.08°C).^91^

#### Indoor temperature

A total of 19 studies with green wall interventions were included in the meta-analysis for indoor temperature^3358^,^61 68^ (see figure 3d). Meta-analysis suggest that green walls significantly decrease the indoor temperature by 2.08°C compared with bare walls (95% CI: −3.00 to –1.16; 17 studies),^3358^,^61 68^ and the subgroup analysis was significant for both green facades (MD: −1.85°C; 95% CI: −3.18°C to –0.52°C; 10 studies)^3358^,^61 68^ and living walls (MD: −2.27°C; 95% CI: −3.76°C to –0.78°C; 21 studies).^58 69 71 72 76 77 80 85 86 94 96 97^ ^87^ Meta-analysis of green walls did not have a statistically significant difference in indoor temperature compared with bricked walls (MD: −1.79°C; 95% CI: −9.06°C to 5.48°C; 2 study).^87^

### Pavements

A total of 19 studies with modification in pavement structures were included in the meta-analysis;^99^,^115^ separate meta-analyses were conducted for the sets of interventions comparing the interventions with various controls including conventional concrete and asphalt, black bricks, grass and soil.

#### Surface temperature

Overall, modified pavements when compared with conventional concrete did not have a statistically significant difference in surface temperature (MD: −1.14°C; 95% CI: −2.91°C to 0.63°C; 9 studies)^99 101 102 105 106 108 109 112 113^ (see figure 3e), while subgroup analysis suggests a significant difference for modifications in concrete materials including porous, water-retaining, and permeable concrete, pavers, and interlocking porous blocks (MD: 1.74°C; 95% CI: 0.81°C to 2.68°C; 4 studies)^99 105 112 113^ and modified pavements including porous and cool pavements (MD: −1.68°C, 95% CI: −2.88°C to –0.48°C; 3 studies)^102 106 113^ compared with conventional concrete. However, other subgroups did not show a significant difference including solar-reflective coatings (MD: 0.46°C; 95% CI: −6.19°C to 7.11°C; 1 study),^109^ metals (MD: −6.74°C, 95% CI: −19.34°C to 5.85°C; 2 studies)^101 108^ and natural materials (MD°C: −3.39, 95% CI: −8.55°C to 1.77°C; 2 studies)^108 112^ when compared with conventional concrete.

Modified pavements significantly decrease the surface temperature by 5.45°C when compared with conventional asphalt (95% CI: −6.75 to –4.15; 8 studies)^100104 107 109^,^112 114^ with a significant decrease in surface temperature for subgroups of water-retaining pavers (MD: −11.35°C; 95% CI: –19.82°C to –2.88°C; 1 study),^111^ coated asphalt (coloured coatings and solar-reflective coats) (MD: −5.33°C; 95% CI: −7.55°C to –3.11°C; 4 studies),^100 107 109 114^ gravel or recycled materials, (MD: −6.87°C; 95% CI: −8.69°C to –5.05°C; 1 study)^110^ and mixtures of cement and asphalt (MD: −5.90°C; 95% CI: −9.12°C to –2.68°C; 1 study)^104^ when compared with conventional asphalt. However, other subgroup analyses did not show a significant difference including modified asphalt (cool, porous and water-retaining asphalts) (MD: −3.35°C, 95% CI: −7.34°C to 0.65°C; 2 studies)^107 112^ and natural materials (MD: −7.13°C, 95% CI: −23.37°C to 9.11°C; 1 study).^112^

Bricks painted varied shades of black (ranging from 80% black to white shades) significantly decrease the surface temperature by 4.5°C compared with bricks painted 100% black (95% CI: −8.37°C to –0.63°C; 1 study).^103^ Meta-analysis of modifications in pavement materials (modified concrete and asphalt) suggests a statistically significant increase in surface temperature by 4.96°C when compared with grass (95% CI: 4.11°C to 5.81°C; 1 study) and a 2.39°C increase when compared with soil (95% CI: 1.53°C to 3.25°C; 1 study).^112^

### Miscellaneous landscape structures

Meta-analysis of vegetated houses with both green roofs and green walls when compared with bare houses did not have a significant difference in indoor temperature.^33 116^ Meta-analysis of shaded structures, made of various structures including shaded sails, did not have improvement in surface^93 117^ or indoor^117^,^122^ temperature when compared with areas with no shade. Meta-analysis of water irrigation systems, including pavement watering systems and irrigation of grass, did not have a significant difference in surface^123^,^125^ or indoor^123 126^ temperature when compared with no irrigation.

### Building modifications

The review included 21 studies on modified construction materials and improved ventilation and assessed its impact on surface and indoor temperature (table 3, figure 3).

**Table 3:** Summary of findings for the effect of modifications in building structures for heat adaptation on surface and indoor temperature

# of studies			Design	Consistency	Generalisability to population of interest	Generalisability to intervention of interest	# of estimates	MD (95% CI)	Comments
Surface temperature									
Building roof modifications compared with conventional roofs									
7		Experimental^3446 128 132^,^134 137^		No heterogeneity	5 studies were conducted in HIC and 1 in LMIC.Ambient temp range:27°C–42°C	The study compared building roof modifications to conventional roofs. Results pooled for different types of interventions.	11	−3.99°C(−5.95°C to to 2.03°C)	Studies included reflective roofs (5), solar-energy roof (1), radiative cooling and aerated concrete (1)
Building ventilation compared with no ventilation									
2	Experimental^135 136^			No heterogeneity	Both studies were conducted in HICsAmbient temp range:22°C–30°C	The study compared building ventilation systems to no ventilation. Results pooled for different types of interventions.	2	0.24°C(−4.80°C to 5.27°C)	Studies included earth-to-air heat exchanger (1) and ventilated windows (1)
Indoor temperature									
Building roof modifications compared with conventional roofs									
6	Experimental^46 127 128 132 133 137^			Considerable heterogeneity, I^2^=76%	4 studies were conducted in HICs, 1 in LMIC.Ambient temp range:27°C–42°C	The study compared building roof modifications to conventional roofs. Results pooled for different types of interventions.	12	−1.14°C(−2.37°C to 0.10°C)	Interventions included reflective roofs (5) and miscellaneous interventions (3)
Building roof modifications compared with metal roofs									
3	Experimental^56 127 129^			Considerable heterogeneity, I^2^=99%	1 study was conducted in HIC, 2 were conducted in LMICsAmbient temp range:28°C–35°C	The study compared building roof modifications to metal roofs. Results pooled for different types of interventions.	9	−1.41°C(−2.31°C to 0.51°C)	Interventions included painted roofs (2), solar reflective roofs (2), plastic (2), recycled materials (2), metals (1)
Building construction modification compared with conventional construction									
4	Experimental^138187^,^189^			Considerable heterogeneity, I^2^=89%	3 studies were conducted in HICs, 1 was conducted in LMICAmbient temp range:23°C–27°C	The study compared building construction modifications to miscellaneous conventional constructions. Results pooled for different types of interventions.	5	−0.40°C(−2.02°C to 1.22°C)	Interventions included various construction modifications (5)
Building ventilation compared with no ventilation									
2	Experimental^135 136^			Moderate heterogeneity, I^2^=65%	Both studies were conducted in HICsAmbient temp range:22°C–30°C	The study compared building ventilation systems to no ventilation. Results pooled for different types of interventions.	2	−3.81°C(−47.25°C to 39.63°C)	Interventions included earth-to-air heat exchanger (1) and ventilated windows (1)
Building construction modification and ventilation compared with no ventilation and conventional construction									
1	Experimental^138^			Considerable heterogeneity, I^2^=96%	The study was conducted in HICAmbient temp: 23°C	The study compared modified building construction and ventilation systems to no ventilation. Results pooled for different types of interventions.	3	−4.96°C(−10.57°C to 0.65°C)	Interventions included various construction modifications combined with night ventilation (3)

HIC, high-income country; LMIC, low-income or middle-income country; MD, mean difference.

### Roofs

A total of 16 studies with modifications in building roofs were included in the meta-analysis for surface and indoor temperature;^3446 50 56 127^,^137^ separate meta-analyses were conducted for different interventions comparing each intervention with various controls including conventional, and metal roofs.

#### Surface temperature

Meta-analysis of interventions with various modification in building roofs materials, including reflective paints, and radiative roofs, significantly decreased the surface temperature by 3.99°C when compared with conventional roofs (95% CI: −5.95°C to –2.03°C; 7 studies)^3446 128 132^,^134 137^ (see figure 3f). Subgroup analysis suggests that reflective paints significantly decreased the surface temperature by 4.55°C when compared with conventional roofs (95% CI: −6.74°C to –2.36°C; 6 studies).^46128 132^,^134 137^

#### Indoor temperature

Meta-analysis conducted for interventions with various modifications in the building roofs materials, including painted, solar-reflective and radiative roofs, did not have a significant difference in indoor temperature when compared with conventional roofs (MD: −1.14°C; 95% CI: −2.37°C to 0.10°C; 6 studies)^46 127 128 132 133 137^ (see figure 3g) or when compared with metal roofs (MD: −1.41; 95% CI: −2.31°C to 0.51°C; 3 studies).^56 127 129^ However, subgroup analysis suggests that reflective paints significantly decreased the surface temperature by 1.69°C when compared with conventional roofs (95% CI: −3.35°C to –0.02°C; 6 studies).^46 127 128 132 133 137^

### Ventilation systems

Improved ventilation systems (phase change material (PCM) ventilated windows and PCM earth-to-air heat exchanger) had no significant difference in surface temperatures (MD: 0.24°C; 95% CI: −4.80°C to 5.27°C; 2 studies) or indoor temperatures (MD: −3.81°C; 95% CI: −47.25°C to 39.63°C; 2 studies)^135 136^(see figure 3b).

### Modification in construction

There was no significant difference in indoor temperatures when modifications in construction, including a combination of light or heavy constructions and with or without ventilation, were compared with lightly constructed houses with no ventilation (MD: −4.96°C; 95% CI: −10.57°C to 0.65°C; 1 study).^138^

### Individual-level adaptation to heat stress

The 45 selected studies targeted human behaviours through HAPs (16 studies), heat education and warning systems (10 studies), modifications in clothing (10 studies) and water-rest-shade (04 studies).^1718 139^,^176^ The studies were further subgrouped according to the intervention (online supplemental table 1).

#### Heat action plans

There were 16 studies which examined the impact of HAPs on heat-related mortality,^139^,^149153 157 158 160 161^ out of which only 1 study was conducted in a low-income or middle-income country (LMIC) (India).^143^ 12 studies examined the national heat prevention programmes using pre and post study design.^139^,^143146 148 153 157 158 160 161^ The results from these studies show a reduction in attributable deaths, mortality deficit and mortality risk. Four studies used quasi-experimental design,^144 145 147 149^ examining the impact of community trials for heat preparedness among elderly populations;^145 147 153^ two of these studies reported an increase in knowledge and improvement in health behaviours,^149 153^ while the other two studies reported a reduction in mortality rates.

##### Heat warning systems

Further, there were three studies examining the impact of heat warnings on heat-related mortality, all of them being conducted in high-income countries (HICs) (USA and Canada).^159 162 163^ Two studies examined the widely implemented heat warning systems using a pre–post study design and reported a decrease in mortality. A single RCT reporting improved knowledge and behaviours due to automated phone messages to targeted populations.^159^

##### Heat education and awareness

Seven studies examined the impact of heat education and awareness, including mass media campaigns and targeted towards specific populations (healthcare providers, agricultural workers, school-going children),^150151 154^,^156 177^ and three of these studies were conducted in LMICs (Pakistan and India).^150 155^ Three of these studies were RCTs, two quasi-experimental and three used pre–post designs. There was a wide range of outcomes, including heat-related mortality and morbidity, and KAP. One study reported a decrease in heat-wave mortality due to repeated use of television and newspaper disseminated messages.^150^ Two studies examining heat-related morbidity reported a decrease in hospital visits by 38%–80%,^151 155^ one study reported a 64% in self-reported heat stress,^154^ and one study reported a decrease in physiological strain index.^156^ Two studies examining KAP reported 6%–22.5% improvements in KAPs related to heat adaptations.^154 177^ Two studies reported an improvement in healthcare providers’ knowledge and skills on climate change and diagnosis of heat-related illnesses.^178 179^

### Modifications in clothing

10 studies assessed modifications in clothing, including light garments and cooling vests,^1718 164^,^170 180^ two of them being conducted in LMICs (India and Iran),^164 180^ with four case-crossover studies^165^,^167169^ and two RCTs.^17 170^ Seven studies, examining the impact of cooling vests on construction, agriculture and other outdoor workers and office employees, reported physical strain alleviation and decrease in body temperature.^17164^,^167 169 180^ Three studies, examining light/loose garments among construction workers, reported physical strain alleviation, decrease in body temperature and increased performance.^18 168 170^

### Water-rest-shade intervention

There were four studies examining the impact of the water-rest-shade strategy among agricultural workers, and all were conducted in LMICs.^171^,^174^ Two studies were conducted using pre–post design^171 172^ and the other two using quasi-experimental designs.^173 174^ Two studies reported an improvement in glomerular filtration rate,^172 173^ two studies reported 70%–75% reduction in the incidence of kidney injury^173 174^ and one study reported a self-reported increase in water consumption by 25% and decrease in heat stress.^171^

### Risk of bias analysis

Most RCTs had a low risk of bias based on randomisation and selective reporting. However, four of six RCTs had a high risk of bias due to lack of blinding. Non-randomised intervention studies had a high risk of bias due to potential for confounding as well as lack of blinding leading to bias in measurement of outcomes (domain-wise risk of bias assessment for all included studies is given in online supplemental annex 5). The overall high risk of bias in these studies underscores the challenges in establishing a clear causal relationship between the interventions targeted to reduce heat stress and the observed outcomes, suggesting that confounding factors may have a significant impact on the results.

### Sensitivity analysis

After excluding Sudimac 2018, the pooled effect size slightly decreased from −2.39 (95% CI: −4.03 to –0.74) to −3.22 (95% CI: −4.37 to –2.07) for green walls compared with bare walls. Similarly, for modified concrete, the pooled effect size slightly increased from −1.14 (95% CI: −2.91 to 0.63) to 0.40 (95% CI: −0.40 to 1.19) compared with conventional roof after removing Elqattan 2021. For modifications in building roofs, the pooled effect size slightly increased from −1.05 (95% CI: −2.20 to 0.10) to −1.00 (95% CI: −2.11 to 0.11) compared with conventional roof after removing Garg 2016. The overall direction and statistical significance of the results remained unchanged in all analyses.

The results of the sensitivity analyses indicate that the overall findings are robust, as the effect sizes remained largely consistent despite the exclusion of studies. While minor variations were observed, the overall conclusions remained unaffected, suggesting that the results are not driven by methodological or study quality differences. Onlinesupplemental annex 6  7 show a comparison of the pooled effect sizes without studies with high risk of bias and outliers, respectively.

## Discussion

This review summarises the findings from 122 studies investigating the impact of modifications in landscape and building structures on surface and indoor temperatures, along with 45 studies investigating the impact of heat adaptation interventions on human indicators (online supplemental panel 2).

The meta-analysis for landscape modifications suggests that green roofs, green walls and asphalt modifications are the most effective interventions to reduce temperatures; green roofs can significantly reduce surface temperatures by 1°C–11°C, and indoor temperature by 1°C–2.4°C under ambient temperature range of 12°C–35°C. Similarly, green walls can significantly decrease the surface temperatures by more than 2°C, and indoor temperature by 1°C–2°C under ambient temperature range of 14°C–35°C. Further, our review suggests that modified asphalt materials and variations in the shades of bricks painted black can significantly reduce surface temperatures by around 5°C under an ambient temperature range of 29°C–34°C.

A recent systematic review on green roofs reported a 1°C–3°C reduction in surface temperatures in various climatic conditions.^23^ Another review on urban greenery also reported similar findings, that is, an average reduction in surface temperature of 2°C for green roofs and 1.8°C for green walls.^14^ In contrast to our review, this systematic review also includes cross-sectional studies in their eligibility criteria. Other systematic reviews reporting the cooling impact of modifications in pavement materials report studies in the laboratory or controlled settings which do not compare to our findings.^15 181 182^

The meta-analysis for building modifications suggests that reflective paints are effective interventions and can significantly reduce the surface temperature by almost 5°C, and indoor temperature by more than 1.5°C under ambient temperature range of 27°C–42°C. A systematic review focusing on the reflective materials reported a 1°C–7°C reduction in indoor temperatures.^183^

Overall, the evidence targeting individual-level adaptation to heat stress suggests a decrease in morbidity and mortality, and improvement in physiological outcomes and knowledge and behaviours. HAPs are a common strategy that many HICs adopted in the early 2000s.^141^ Our findings suggest that HAPs and heat warning systems positively impact overall mortality and morbidity. Similar findings were reported by another systematic review, however, it only focused on older populations.^184^ Most of these studies included in this subgroup are ecological studies, so causality cannot be established. There is a potential of wide variation in implementation magnitude due to variations in heat thresholds, allocation of resources and response capacities in the various countries across the world. Further, care should be taken while inferring results as HAPs are often tailored to local needs and resources.

Similarly, our review reports an improvement in knowledge about heat-related health behaviours and decreased morbidity from heat awareness campaigns; similar findings were reported by a review summarising the heat adaptation strategies in South Asia.^21^ Our review also suggests that modifications in clothing, including light fabrics, ventilated clothes and cooling vests, lead to a reduction in heat morbidity, especially for heat-vulnerable occupations like construction and agriculture workers. Similarly to our findings, Jay *et al* also recommended ventilated, breathable and lightweight clothing to reduce the health effects of extreme heat.^13^ Lastly, our review suggests that the water-rest-shade strategy can improve kidney function among agricultural workers. A review targeting construction workers recommended limiting work hours to reduce the health impacts of heat stress.^185^ Most systematic reviews have summarised implementation and adoption of strategies only. Few reviews that have focused on the health impact have not synthesised the overall effect of strategies targeting individual-level adaptation.

This is one of the most comprehensive attempts to synthesise knowledge on interventions targeting heat adaptation. The study recommends the implementation of heat adaptation strategies including green roofs and walls at a large scale to reduce the harmful effects of extreme heat. The review only included studies conducted in real-life situations rather than studies conducted in climate-controlled environments or as simulated models.

There was heterogeneity indicating variation among studies included in the review, hence we applied a random effects model and conducted various subgroup and sensitivity analyses. The findings were interpreted, acknowledging the impact of heterogeneity and emphasising the need for further research with standardised methodologies to improve consistency across studies.

Most of the studies included in our review were conducted in HICs; however, a few were conducted in LMICs. Hence, there is a need to replicate the interventions in LMIC settings. All studies are conducted as small-scale experiments in natural settings, except one which examined shade sails using randomised design. There is a need to examine their effectiveness on larger scales and in community settings to confirm the magnitude of the impact. Further, none of the studies explored the impact of landscape and building modifications on human physiological outcomes.

Most of the landscape interventions, particularly green roofs and green walls, require long-term maintenance. Hence, cost can be a major factor in green landscaping in LMIC settings.^186^ There is a need to analyse the impact of green spaces on heat mitigation at the various stages of growth stages. Similarly, there is a need to analyse the impact of pavements at various stages, from initial newly developed structures to weathered conditions.

## Conclusions

The review underscores the effectiveness of green roofs, green walls, reflective paints and asphalt modifications in reducing surface and indoor temperatures, besides the promise of community education, early warning systems and HAPs in reducing morbidity and mortality. These research findings emphasise the complexity of factors influencing the effectiveness of heat adaptation interventions. While certain modifications show promise in reducing temperatures and health outcomes, the varied outcomes across different interventions highlight the need for contextual targeted approaches. To enhance their impact, urban planners, architects and policy-makers should incorporate climate-friendly designs which are favourable in the specific contexts. Furthermore, investing in long-term research and monitoring will provide valuable insights into acceptance, durability, cost-effectiveness and broader health benefits to the population at large.

## Supplementary material

10.1136/bmjph-2024-002332 online supplemental file 1

10.1136/bmjph-2024-002332 online supplemental file 2

10.1136/bmjph-2024-002332 online supplemental file 3

10.1136/bmjph-2024-002332 online supplemental file 4

10.1136/bmjph-2024-002332 online supplemental file 5

10.1136/bmjph-2024-002332 online supplemental file 6

10.1136/bmjph-2024-002332 online supplemental file 7

10.1136/bmjph-2024-002332 online supplemental file 8

10.1136/bmjph-2024-002332 online supplemental file 9

## Data Availability

Data sharing not applicable as no datasets generated and/or analysed for this study.
