# Supplementary material for: Evaluation of community-based heat adaptation interventions: a systematic review
Source: BMJ Public Health. 2025 Jul 15;3(2):e002332. doi: 10.1136/bmjph-2024-002332 (PMC12273142; doi:10.1136/bmjph-2024-002332)
Supplement: online supplemental file 1 [file bmjph-3-2-s001.pdf]

**Panel 1: Categorization and description of heat adaptation interventions included in the study.**

| S.No. | | Name | Description |
|---|---|---|---|
| | | **Landscape Modification** | |
| **Green walls** | | | |
| A | | **Green Facades** | Green facades can be categorized as direct or indirect. |
| | 1. | Direct Green Facades | Direct green facades utilize the evergreen or deciduous climbers that attach themselves directly to the building surface. This method represents the conventional approach to greening facades, where climbing plants are directly affixed to the building's façade, utilizing the building materials for support. The plants typically have their roots in the ground or planter boxes[88,190]. |
| | 2. | Indirect Green Facades | Indirect green facades include a vertical support structure for climbing plants development which is supported by trellis or steel cables. In this way, plants are guided to develop along the support structure[88,190]. |
| B | | **Living Walls** | Living walls are composed of modular pre-cultivated panels, each containing a growing medium and an irrigation system that supplies all the necessary nutrients for plant growth[190]. Living wall systems use planter boxes and support structures, often incorporating pre-vegetated and prefabricated arrangements. These are designed to facilitate the growth of plants that have specific nutritional requirements while affixing to walls[88]. They also incorporate a waterproof layer to safeguard the facade against moisture penetration[190]. |
| **Green Roofs** | | | A green roof comprises a series of distinct layers that collectively create a conducive environment for cultivating vegetation on a level or inclined rooftop. The layers of a green roof, arranged from the uppermost to the lowermost, consist of the vegetation layer, growing medium, filter, drainage (for moisture retention), root barrier, and ultimately a waterproofing membrane atop a structural deck[191]. |
| | 1. | Extensive | The extensive variety features a relatively thin layer of growing medium, typically ranging from 6 to 20 cm in thickness. It commonly supports the growth of moss, sedums, herbs, and grass, and demands minimal maintenance[190]. |
| | 2. | Semi-intensive | The semi-intensive green roof is an amalgamation of the extensive and intensive types, but with a condition that the extensive portion must account for 25% or less of the overall green roof area[190]. |
| | 3. | Intensive | The intensive green roof necessitates a greater depth of growing medium, typically ranging from 20 to 100 cm. It also calls for irrigation and continuous maintenance[190]. |
| **Pavements** | | | |
| | 1. | Cool pavement | Paving materials designed to reflect increased solar energy, boost water evaporation, or undergo modifications to maintain a cooler temperature compared to traditional pavements[192]. |
| | 2. | Reflective pavement | Seal treatments that resurface pavements with light-colored materials increase the solar reflectance of the pavements[192.] |
| | 3. | Porous pavers | Porous pavers incorporate a cellular grid system with internal openings filled with soil, sand, gravel, or grass to retain moisture that serve as channels for water permeation[193]. |
| | 4. | Permeable pavers | Permeable pavers are composed of a layer of concrete or fired-clay bricks, allowing rainwater passing around the paver rather than through it[193]. |
| | 5. | Water-retaining pavements | Water-retentive pavements are designed to primarily retain water at the top layer[193]. |
| | | **Modifications in Building Structure** | |
| **Building Construction** | | | |
| | 1. | Reflective coatings | Highly reflective coating reduces building's heat gain by reflecting incoming solar radiations[13]. |
| | 2. | Super-cool coatings | Super-cool coatings increase the longwave radiative heat loss from the buildings by focusing their radiative emissions within an 'atmospheric window' of certain wavelengths[13]. |

| | | | |
|---|---|---|---|
| **Building Ventilation** | | | |
| | 1. | Natural ventilation | Strategically oriented paired inlets and outlets in the building facade with prevailing winds during the hottest can boost evaporative heat losses directly from the building occupants [13]. |
| | | **Humans Adaptation** | |
| **Heat action plan** | | | Integrated interventions collaborated with multiple stakeholders and includes common components like warning systems, heat-related awareness, an emergency plan, healthcare capacity building and cooling facilities to the public[13]. |
| | 1. | Heat warning system | Heat wave warnings typically issued when the temperatures reach levels that pose a significant risk to the population[159], |
| | 2. | Heat education | Health education and awareness generation through the dissemination of information, education and communication (IEC) material on heat waves to bring behavioral changes so that people expose less to heat[150]. |
| **Cloth** | | | |
| | 1. | Cool garments | Incorporate a liquid cooling system or electric fan within the fabric layers, featuring a jacket-like enclosure that can help cool an individual against heat during work[164,166]. |
| **Water-rest-shade** | | | OSHA's guidelines to prevent dehydration and heat stress without decreasing productivity for people working in hot temperatures[194]. |

| Panel 2: Sub-group analysis for heat adaptation interventions included in the meta-analysis. | | | | | | |
|---|---|---|---|---|---|---|
| Interventions Groups | Interventions | Comparison Group | Surface Temperature | | Indoor Temperature | |
| | | | # of studies (estimates) | MD (95% CI) | # of studies (estimates) | MD (95% CI) |
| **Landscape interventions** | | | | | | |
| Green Roofs | Green roofs | Conventional Roof | 17 (23) | -10.88°C (-15.26, -6.50) | 12 (15) | -2.40°C (-3.54, -1.26) |
| | Green roofs | Bare Substrate | 2 (39) | -1.16°C (-1.38, -0.94) | - | - |
| | Green roof, soil substrate | Metal Roof | - | - | 1 (2) | -0.69°C (-1.71, 0.32) |
| | Green roofs, sand, pellet substrate | Wood Roof | 2 (3) | -0.59°C (-1.98, 0.79) | 2 (5) | -0.83°C (-1.32, -0.34) |
| | Green roofs, extensive green roofs | Painted Roof | 4 (5) | -3.28°C (-7.18, 0.62) | 2 (3) | -3.21°C (-7.50, 1.08) |
| | Modular green roofs | EPDM Membrane | 1 (9) | -1.77°C (-2.27, -1.28) | - | - |
| Green Walls | Green facades | Bare Wall | 15 (17) | -2.12°C (-3.10, -1.14) | 8 (10) | -1.85°C (-3.18, -0.52) |
| | Living walls | | 23 (26) | -2.29°C (-4.99, 0.40) | 12 (12) | -2.27°C (-3.76, -0.78) |
| | Green facades | Bricked Wall | 6 (8) | -1.87°C (-2.87, -0.87) | 1 (2) | -1.79°C (-9.06, 5.48) |
| | Living walls | | 2 (3) | -2.87°C (-3.63, -2.11) | - | - |
| | Green facades | Galvanized Plate | 1 (5) | -0.47°C (-1.03, 0.08) | - | - |
| | Living wall | Double Glazed Facade | - | - | 1 (4) | -0.75°C (-0.88, -0.62) |
| Modifications in Pavement structure | Modifications in concrete | Conventional Concrete | 4 (11) | 1.74°C (0.81, 2.68) | - | - |
| | Modifications in pavers | | 3 (8) | -1.68°C (-2.88, -0.48) | - | - |
| | Solar-reflective coatings | | 1 (2) | 0.46°C (-6.19, 7.11) | - | - |
| | Metals | | 2 (4) | -6.74°C (-19.34, 5.85) | - | - |
| | Natural materials | | 2 (3) | -3.39°C (-8.55, 1.77) | - | - |
| | Cystosepiment | | 1 (1) | 0.16°C (-10.97, 11.28) | - | - |
| | Modifications in asphalt | Conventional Asphalt | 2 (6) | -3.35°C (-7.34, 0.65) | - | - |
| | Modifications in pavers | | 1 (2) | -11.35°C (-19.82, -2.88) | - | - |
| | Coatings | | 4 (9) | -5.33°C (-7.55, -3.11) | - | - |
| | Gravel | | 1 (3) | -6.87°C (-8.69, -5.05) | - | - |
| | Natural materials | | 1 (2) | -7.13°C (-23.37, 9.11) | - | - |
| | Cement mixtures | | 1 (4) | -5.90°C (-9.12, -2.68) | - | - |
| | Variation in gravel thickness | White Gravel | 1 (3) | -4.46°C (-10.61, 1.69) | - | - |
| | Variation in shades of bricks | Black Brick | 1 (4) | -4.50°C (-8.37, -0.63) | - | - |

| | | | | | | |
|---|---|---|---|---|---|---|
| | Modifications in concrete | Grass | 1 (5) | 4.87°C (3.82, 5.93) | - | - |
| | Modifications in asphalt | | 1 (3) | 5.11°C (1.48, 8.74) | - | - |
| | Modifications in concrete | Soil | 1 (5) | 2.29°C (1.23, 3.35) | - | - |
| | Modifications in asphalt | | 1 (3) | 2.57°C (-1.12, 6.27) | - | - |
| Vegetated House | Green house (Roof & Walls) | Bare House | - | - | 2 (3) | -3.16°C (-11.19, 4.86) |
| Shade | Shades | Unshaded | 2 (2) | -0.64°C (-2.15, 0.86) | 7 (12) | -0.10°C (-0.43, 0.23) |
| Water Irrigation Systems | Irrigation systems | No Irrigation | 3 (3) | -2.79°C (-22.75, 17.15) | 2 (2) | -1.99°C (-26.75, 22.78) |
| **Building interventions** | | | | | | |
| Modifications in Building Roof | Reflective Roofs | Conventional Roof | 4 (9) | -4.55°C (-6.74, -2.36) | 6 (8) | -1.69°C (-3.35, -0.02) |
| | Aerated & Radiative cooling | | 2 (3) | -2.26°C (-11.78, 7.26) | - | - |
| | Solar-reflective | Galvanized Steel Panel | 1 (1) | -5.59°C (-6.06, -5.09) | - | - |
| | | Urethane-based Waterproofing | 1 (1) | -2.45°C (-2.97, -1.93) | 1 (1) | -0.56°C (-0.93, -0.19) |
| | | Tin | - | - | 1 (1) | -1.10°C (-1.81, -0.39) |
| | | Asbestos | - | - | 1 (1) | 1.00°C (0.33, 1.67) |
| | Supercool Roof | White Cool Roof | 1 (1) | -3.29°C (-9.51, 2.94) | - | - |
| | Painted Roofs | Bare Metal | - | - | 1 (1) | -0.78°C (-0.80, -0.76) |
| | | Green Paint | - | - | 1 (1) | -5.00°C (-10.90, 0.90) |
| | Recycled Materials | Tin | - | - | 1 (1) | -2.00°C (-2.73, -1.27) |
| | | Asbestos | - | - | 1 (1) | -3.03°C (-4.20, -1.86) |
| | Plastic | Tin | - | - | 1 (1) | -1.94°C (-2.46, -1.42) |
| | | Asbestos | - | - | 1 (1) | -2.38°C (-3.33, -1.43) |
| | Thermoshield Sheet | Conventional Steel Sheet | - | - | 1 (1) | -0.93°C (-4.65, 2.78) |
| Building Ventilation Systems | Ventilated Systems | Phase change material | 2 (2) | 0.24°C (-4.80, 5.27) | 2 (2) | -3.81°C (-47.25, 39.63) |
| | Airlite ventilation | Conventional Roof | - | - | 1 (1) | 4.74°C (3.59, 5.89) |
| | | Tin | - | - | 1 (1) | 2.66°C (1.58, 3.74) |
| | | Asbestos | - | - | 1 (1) | 2.50°C (1.35, 3.65) |
| | 24 hours ventilation, misting | Passive cooling, dripping ventilation | - | - | 1 (1) | 3.21°C (2.08, 4.34) |
| | Underground Heat Exchanger | Transpired solar cooling system | - | - | 1 (1) | -6.98°C (-7.72, -6.24) |

| | | | | | | |
|---|---|---|---|---|---|---|
| Modifications in Building Construction | Very heavy construction | Light Constructions | - | - | 1 (1) | -2.44°C (-3.19, -1.70) |
| | Heavy construction, night ventilation | | - | - | 1 (3) | -4.96°C (-10.57, 0.65) |
| | Misc construction | Micro-ventilated Facade | - | - | 1 (2) | 0.28°C (-4.40, 4.96) |
| | CSEB Walls/RCC Slab Roof | CMU Walls/Asbestos Cement Roof | - | - | 1 (1) | -0.40°C (-8.85, 8.05) |
| | EPS Insulation | Energy efficient homes, no upgrades | - | - | 1 (1) | 0.16°C (-0.20, 0.52) |

MD=Mean Difference
EPDM= EPDM: Ethylene Propylene Diene Monomer
