# Supplementary material for: Evaluation of community-based heat adaptation interventions: a systematic review
Source: BMJ Public Health. 2025 Jul 15;3(2):e002332. doi: 10.1136/bmjph-2024-002332 (PMC12273142; doi:10.1136/bmjph-2024-002332)
Supplement: online supplemental file 2 [file bmjph-3-2-s002.docx]

**ANNEX 1: Search Strategy**

**Search strategies for the “Heat Stroke Interventions” project**

Below are the search strategies along with the number of hits in each of the following databases:

- • PubMed
- • CINAHL
- • Cochrane
- • Lilac
- • Scopus
- • Clinicaltrials.gov

**Note:** The databases “Embase” and “Web of science” do not appear to have subscription for the university currently.

**1. PubMed NLM search strategy:**

("Heat Stroke"[Mesh] OR "Heat Stress Disorders"[Mesh] OR "Heat Exhaustion"[Mesh] OR “Climate Change"[Mesh] OR "Hot Temperature"[Mesh] OR “Microclimate”[Mesh] OR “Climate”[Mesh] OR “Urban heat” OR “heat stress” OR “Climatic change” OR “Heat radiation” OR “Temperature rise” OR “Heat waves” OR “Heat cramps” OR “Heat collapse” OR “Heat illness” OR “Heat injury” OR “Heat-related illness” OR “Heat stress syndrome” OR “Heat syncope” OR “Heat stroke” OR “Thermal strain” OR “Thermal stress” OR “Uncompensable heat stress” OR “Heat strain” OR “Extreme heat”) AND (“Meteorology"[Mesh] OR "Air Conditioning"[Mesh] OR "Ergonomics"[Mesh] OR "Facility Design and Construction"[Mesh] OR “Thermosensing”[Mesh] OR “Ventilation”[Mesh] OR “Gastric hypothermia”[Mesh] OR “Efficiency, Organizational”[Mesh] OR “Heat-health prevention" OR “misting” OR “active outreach” OR “healthcare prepar*” OR “Green planting” OR “renewable energ*” OR “fans” OR “refrigerators" OR ice OR "capacity building” OR “heat management” OR “Reflective paint*” OR “Albedo” OR “Artificial intelligence” OR “Trees” OR “Vegetation” OR “Passive cooling” OR “reflectivity” OR “Water spray” OR “Wet Sheets” OR “ice-water” OR “Cooling blanket” OR “Water baths” OR “Community mobilization” OR “Clothing” OR “Light clothing” OR “Cooling” OR “Heat (stress) management” OR “Multimodal interventions” OR “Community care programmes” OR “early warning systems” OR “community-led initiatives” OR “effective communication” OR “Cooling vests” OR “Cooling jackets” OR “Cold shower” OR “Ice sheet*” OR “cooling devices” OR “reflective blankets” OR “evaporative cooling” OR “ice packs” OR “cold water immersion” OR “intravenous fluids” OR “cooling vest” OR “Community-Based Interventions” OR “heat monitoring” OR “campaign*” OR “mobilization” OR “education campaign*” OR “awareness campaign*” OR “intranasal cooling” OR “Neighborhood

Interventions” OR “Cool coatings”)

**Filters applied:** Humans

**# of Results:** 8,840

9020 (12/23/2022)

535 (2/29/2023)

**2. EBSCO CINAHL Complete search strategy:**

(MH "Heat Stroke" OR MH "Heat Stress Disorders” OR MH "Heat Exhaustion” OR MH “Climate Change” OR MH "Hot Temperature” OR MH “Microclimate” OR MH “Climate” OR “Urban heat” OR “heat stress” OR “Climatic change” OR “Heat radiation” OR “Temperature rise” OR “Heat waves” OR “Heat cramps” OR “Heat collapse” OR “Heat illness” OR “Heat injury” OR “Heat-related illness” OR “Heat stress” OR “Heat stress disorder” OR “Heat stress syndrome” OR “Heat syncope” OR “Heat stroke” OR “Thermal strain” OR “Thermal stress” OR “Uncompensable heat stress” OR “Heat strain” OR “Extreme heat”) AND (MH “Meteorology” OR MH "Air Conditioning” OR MH "Ergonomics” OR MH "Facility Design and Construction” OR MH “Thermosensing” OR MH “Ventilation” OR MH “Gastric hypothermia” OR MH “Efficiency, Organizational” OR “Heat-health prevention" OR “misting” OR “active outreach” OR “healthcare prepar*” OR “Green planting” OR “renewable energ*” OR “fans” OR “refrigerators" OR ice OR "capacity building” OR “heat management” OR “Reflective paint*” OR “Albedo” OR “Artificial intelligence” OR “Trees” OR “Vegetation” OR “Clothing” OR “Light clothing” OR “Cooling” OR “Heat (stress) management” OR “Multimodal interventions” OR “Community care programmes” OR “early warning systems” OR “community-led initiatives” OR “effective communication” OR “Cooling vests” OR “Cooling jackets” OR “Cold shower” OR “Ice sheet*” OR “cooling devices” OR “reflective blankets” OR “evaporative cooling” OR “ice packs” OR “cold water immersion” OR “intravenous fluids” OR “cooling vest” OR “Community-Based Interventions” OR “heat monitoring” OR “campaign*” OR “mobilization” OR “education campaign*” OR “awareness campaign*” OR “intranasal cooling” OR “Neighbourhood Interventions” OR “Cool coatings” OR “Passive cooling” OR “reflectivity” OR “Cooling blanket” OR “Water baths” OR “Community mobilization”)

HITS: 246 (1/4/2023)

HITS: 19 (2/29/2024)

(MH "Heat Stroke" OR MH "Heat Stress Disorders” OR MH "Heat Exhaustion” OR MH “Climate Change” OR MH "Hot Temperature” OR MH “Microclimate” OR MH “Climate” OR “Urban heat” OR “heat stress” OR “Climatic change” OR “Heat radiation” OR “Temperature rise” OR “Heat waves” OR “Heat cramps” OR “Heat collapse” OR “Heat illness” OR “Heat injury” OR “Heat-related illness” OR “Heat stress” OR “Heat stress disorder” OR “Heat stress syndrome” OR “Heat syncope” OR “Heat stroke” OR “Thermal strain” OR “Thermal stress” OR “Uncompensable heat stress” OR “Heat strain” OR “Extreme heat”) AND (MH "Air Conditioning” OR MH "Ergonomics” OR MH "Facility Design and Construction” OR MH “Thermosensing” OR MH “Ventilation” OR MH “Gastric hypothermia” OR “Heat-health prevention" OR “misting” OR “active outreach” OR “healthcare prepar*” OR “fans” OR “refrigerators" OR ice OR "capacity building” OR “heat management” OR “Clothing” OR “Light clothing” OR “Cooling” OR “Heat (stress) management” OR “Multimodal interventions” OR “Community care programmes” OR “early warning systems” OR “community-led initiatives” OR “effective communication” OR “Cooling vests” OR “Cooling jackets” OR “Cold shower” OR “Ice sheet*” OR “cooling devices” OR “reflective blankets” OR “evaporative cooling” OR “ice packs” OR “cold water immersion” OR “intravenous fluids” OR “cooling vest” OR “Community-Based Interventions” OR “heat monitoring” OR “campaign*” OR “mobilization” OR “education campaign*” OR “awareness campaign*” OR “intranasal cooling” OR “Neighbourhood Interventions” OR “Cooling blanket” OR “Water baths” OR “Community mobilization”)

HITS: 214

**3. Wiley Cochrane Library search strategy:**

Search Name: Heat Stroke Interventions_vF

Date Run: 22/08/2022 12:04:33

| Comment: 22.08.2022_vF_ Final Cochrane search strategy ID# | Search | | Hits |
| --- | --- | --- | --- |
| #1 | | MeSH descriptor: [Heat Stroke] explode all trees | |
| #2 | MeSH descriptor: [Heat Stress Disorders] explode all trees | | 207 |
| #3 | MeSH descriptor: [Heat Exhaustion] explode all trees | | 17 |
| #4 | MeSH descriptor: [Climate Change] explode all trees | | 10 |
| #5 | MeSH descriptor: [Hot Temperature] explode all trees | | 1991 |
| #6 | MeSH descriptor: [Microclimate] explode all trees | | 8 |
| #7 | #1 OR #2 OR #3 OR #4 OR #5 OR #6 | | 2147 |
| #8 | MeSH descriptor: [Air Conditioning] explode all trees | | 36 |
| #9 | MeSH descriptor: [Ergonomics] explode all trees | | 3241 |
| #10 | MeSH descriptor: [Facility Design and Construction] explode all trees | | 207 |
| #11 | MeSH descriptor: [Thermosensing] explode all trees | | 163 |
| #12 | MeSH descriptor: [Ventilation] explode all trees | | 88 |
| #13 | MeSH descriptor: [Gastric Hypothermia] explode all trees | | 0 |
| #14 | MeSH descriptor: [Efficiency, Organizational] explode all trees | | 129 |
| #15 | (cooling*):ti,ab,kw OR (Vegetation*):ti,ab,kw OR (Trees):ti,ab,kw AND (ice*):ti,ab,kw | | 3029 |
| #16 | #8 OR #9 OR #10 OR #11 OR #12 OR #13 OR #14 OR #15 | | 6802 |
| #17 | #7 AND #16 | | 227 |

**4. Embase:** Subscription not available for the Aga Khan University

**5. Lilac search strategy:**

(mh: "Heat Stroke" OR mh: "Heat Stress Disorders” OR mh: "Heat Exhaustion” OR mh: “Climate Change” OR mh: “Microclimate” OR mh: “Climate” OR “Urban heat” OR “heat stress” OR “Climatic change” OR “Heat radiation” OR “Temperature rise” OR “Heat waves” OR “Heat cramps” OR “Heat collapse” OR “Heat illness” OR “Heat injury” OR “Heat-related illness” OR “Heat stress” OR “Heat stress disorder” OR “Heat stress syndrome” OR “Heat syncope” OR “Heat stroke” OR “Thermal strain” OR “Thermal stress” OR “Uncompensable heat stress” OR “Heat strain” OR “Extreme heat) AND (Cooling OR mh: “Gastric hypothermia” OR mh: “Ventilation” OR Relectiv* OR Communi*)

**# of Results:** 42 44 (12/23/2022)

4 (2/29/23)

(mh: "Heat Stroke" OR mh: "Heat Stress Disorders” OR mh: "Heat Exhaustion” OR mh: “Climate Change” OR mh: “Microclimate” OR mh: “Climate” OR “Urban heat” OR “heat stress” OR “Climatic change” OR “Heat radiation” OR “Temperature rise” OR “Heat waves” OR “Heat cramps” OR “Heat collapse” OR “Heat illness” OR “Heat injury” OR “Heat-related illness” OR “Heat stress” OR “Heat stress disorder” OR “Heat stress syndrome” OR “Heat syncope” OR “Heat stroke” OR “Thermal strain” OR “Thermal stress” OR “Uncompensable heat stress” OR “Heat strain” OR “Extreme heat) AND (Cooling OR mh: “Gastric hypothermia” OR mh: “Ventilation” OR Relectiv* OR Communi* OR

(mh: "Heat Stroke" OR mh: "Heat Stress Disorders” OR mh: "Heat Exhaustion” OR mh: “Climate Change” OR mh: “Microclimate” OR mh: “Climate” OR “Urban heat” OR “heat stress” OR “Climatic change” OR “Heat radiation” OR “Temperature rise” OR “Heat waves” OR “Heat cramps” OR “Heat collapse” OR “Heat illness” OR “Heat injury” OR “Heat-related illness” OR “Heat stress” OR “Heat stress disorder” OR “Heat stress syndrome” OR “Heat syncope” OR “Heat stroke” OR “Thermal strain” OR “Thermal stress” OR “Uncompensable heat stress” OR “Heat strain” OR “Extreme heat) AND (Cooling OR mh: “Gastric hypothermia” OR mh: “Ventilation” OR Relectiv* OR Communi* OR "meteorology" OR "air conditioning" OR "ergonomics" OR "Facility Design and Construction" OR "thermosensing" OR "organizational efficiency" OR "heat health prevention" OR "misting" OR "active outreach" OR "healthcare prepar*" OR "Green planting" OR "renewable energy" OR "fans" OR "refrigerators" OR "ice" OR "capacity building" OR "heat management" OR "Reflective paint*" OR "Albedo" OR "Artificial intelligence" OR "Trees" OR "Vegetation" OR "Passive cooling" OR "reflectivity" OR "Water spray" OR "Wet Sheets" OR "ice-water" OR "Cooling blanket" OR "Water baths" OR "Community mobilization" OR "Clothing" OR "Light clothing" OR "Cooling" OR "Heat (stress) management" OR "Multimodal interventions" OR "Community care programmes" OR "early warning systems" OR "community-led initiatives" OR "effective communication" OR "Cooling vests" OR "Cooling jackets" OR "Cold shower" OR "Ice sheet*" OR "cooling devices" OR "reflective blankets" OR "evaporative cooling" OR "ice packs" OR "cold water immersion" OR "intravenous fluids" OR "cooling vest" OR "Community-Based Interventions" OR "heat monitoring" OR "intranasal cooling" OR "Neighborhood Interventions" OR "Cool coatings")

HITS 59 (1/4/2023) English: 24

**6. Web of science:** Subscription not available for the Aga Khan University

**7. Scopus search strategy:**

("Heat Stroke" OR "*Heat Stress*" OR "Heat Exhaustion" OR "Hot Temperature" OR “Urban heat” OR “Heat radiation” OR “Temperature rise” OR “Heat waves” OR “Heat cramps” OR “Heat collapse” OR “Heat illness” OR “Heat injury” OR “Heat-related illness” OR “Heat syncope” OR “Heat strain” OR “Extreme heat”) AND (cooling* OR "air condition*" OR thermosens* OR ventilation OR "gastric hypothermia" OR misting OR outreach OR ergonomics OR fans OR refrigerat* OR ice* OR *campaign OR Trees OR management OR vegetation)

**Filters applied:** Medicine, Social Sciences **# of Results:** 6,523

6801 (12/23/2022)

English: 6394

833 (2/29/2024)

**8. Clinicaltrials.gov**

**Condition or disease:**

1. “Heat Stroke” OR “Climate Change” OR “Hot Temperature” OR “Heat strain” OR “Heat waves” OR “Heat illness” OR “Heat-related illness” OR “Heat stress” OR “Heat tolerance” OR “Heat syncope” OR “Extreme heat”
2. "Heat Exhaustion" OR “Microclimate” OR “Urban heat” OR “Climatic change” OR “Heat radiation” OR “Temperature rise” OR “Heat collapse” OR “Heat injury” OR “Thermal strain” OR “Thermal stress” OR “Uncompensable heat stress” OR “Extreme heat”

**Intervention/condition:**

1. cool OR ventilate OR mist OR Ergonomic OR Fans OR Ice OR “Cold environment” OR “Chemical cold pack” OR “Cold water immersion” OR “Green areas” OR “Food choice” OR “Food intake” OR “Intravascular volume administration”
2. “Community-based Home Heat bundle” OR “Clothing” OR “Wet-towel” OR Wetting OR Humid OR “Heat Education” OR “Early warning” OR "multimodal interventions" OR "artificial intelligence" OR "meteorology" OR "thermosensing" OR "vegetation"
3. "organizational efficiency" OR "facility design and construction" OR "reflective paint" OR "albedo" OR "passive cooling" OR "reflectivity" OR "cool coatings" OR "green planting" OR "trees" OR "renewable energy" OR "heat management" OR "heat monitor"

1. "heat health prevention" OR "heat stress management" OR "air conditioning" OR "refrigerators" OR "effective communication" OR "active outreach" OR "community care programs" OR "community led initiatives" OR "community based interventions"
2. "campaign" OR "mobilization" OR "education campaign" OR "awareness campaign" OR "neighborhood interventions" OR "cooling vests" OR "cooling jackets" OR "cold shower" OR "ice sheet" OR "cooling devices" OR "reflective blankets" OR "water sprays"
3. “Wet Sheets” OR “ice water” OR "cooling blankets" OR "water baths" OR "evaporative cooling" OR "ice packs" OR "capacity building" OR "gastric hypothermia" OR "cold water immersion" OR "intravenous fluids" "intranasal cooling"

HITS: (1/3/2023)

a1: 54

a2: 26

a3: 8

a4: 3

a5: 8

a6: 11

b1: 35

b2: 15

b3: 5

b4: 1

b5: 8

b6: 5

“Multimodal interventions” OR “Artificial intelligence” OR “Meteorology" OR “Efficiency, Organizational” OR "Ergonomics" OR "Facility Design and Construction" OR “Thermosensing” OR “Reflective paint*” OR “Albedo” OR “Passive cooling” OR “reflectivity” OR “Cool coatings” OR “Green planting” OR “Trees” OR “Vegetation” OR “renewable energ*” OR

“Heat-health prevention" OR “heat management” OR “Heat (stress) management” OR “heat monitoring” OR

“Cooling” OR "Air Conditioning"[Mesh] OR “fans” OR “refrigerators" OR ice OR “misting” OR “Ventilation”[Mesh] OR

“effective communication” OR “active outreach” OR OR “Community care programmes” OR “community-led initiatives” OR “Community-Based Interventions” OR “campaign*” OR “mobilization” OR “education campaign*” OR “awareness campaign*” OR “early warning systems” OR “Neighborhood

Interventions” OR

“Clothing” OR “Light clothing” OR “Cooling vests” OR “Cooling jackets” OR “Cold shower” OR “Ice sheet*” OR “cooling devices” OR “reflective blankets” OR “Water spray” OR “Wet Sheets” OR “ice-water” OR “Cooling blanket” OR “Water baths” OR “evaporative cooling” OR “ice packs” OR “cooling vest” OR

"capacity building” OR “healthcare prepar*” OR

“Gastric hypothermia”[Mesh] OR “cold water immersion” OR “intravenous fluids” OR “intranasal cooling” OR )

<https://pubmed.ncbi.nlm.nih.gov/?term=food+insecurity+experience+scale&filter=pubt.randomizedcontrolledtrial&filter=pubt.systematicreview&filter=lang.english>

<https://www.cochranelibrary.com/advanced-search/search-manager>

<https://www.scopus.com/results/results.uri?sort=plf-f&src=s&st1=%22food+insecurity+experience+scale%22+AND+validity&sid=e60c6d852048b48b6c345d5036d656a1&sot=b&sdt=b&sl=62&s=TITLE-ABS-KEY%28%22food+insecurity+experience+scale%22+AND+validity%29&origin=searchbasic&editSaveSearch=&yearFrom=Before+1960&yearTo=Present>

<https://clinicaltrials.gov/ct2/results?cond=%E2%80%9CHeat+Stroke%E2%80%9D+OR+%E2%80%9CClimate+Change%E2%80%9D+OR+%E2%80%9CHot+Temperature%E2%80%9D+OR+%E2%80%9CHeat+strain%E2%80%9D+OR+%E2%80%9CHeat+waves%E2%80%9D+OR+%E2%80%9CHeat+illness%E2%80%9D+OR+%E2%80%9CHeat-related+illness%E2%80%9D+OR+%E2%80%9CHeat+stress%E2%80%9D+OR+%E2%80%9CHeat+tolerance%E2%80%9D+OR+%E2%80%9CHeat+syncope%E2%80%9D+OR+%E2%80%9CExtreme+heat%E2%80%9D+&term=&intr=cool+OR+ventilate+OR+mist+OR+Ergonomic+OR+Fans+OR+Ice+OR+%E2%80%9CCold+environment%E2%80%9D+OR+%E2%80%9CChemical+cold+pack%E2%80%9D+OR+%E2%80%9CCold+water+immersion%E2%80%9D+OR+%E2%80%9CGreen+areas%E2%80%9D+OR+%E2%80%9CFood+choice%E2%80%9D+OR+%E2%80%9CFood+intake%E2%80%9D+OR+%E2%80%9CIntravascular+volume+administration%E2%80%9D+&cntry=&state=&city=&dist=&Search=Search>

13 (2/29/2024)

[https://pesquisa.bvsalud.org/portal/?u_filter%5B%5D=fulltext&u_filter%5B%5D=db&u_filter%5B%5D=mj_cluster&u_filter%5B%5D=type_of_study&u_filter%5B%5D=la&fb=&output=site&lang=en&from=1&sort=&format=summary&count=100&page=1&skfp=true&index=&q=%28mh%3A+%22Heat+Stroke%22+OR+mh%3A+%22Heat+Stress+Disorders%E2%80%9D+OR+mh%3A+%22Heat+Exhaustion%E2%80%9D+OR+mh%3A+%E2%80%9CClimate+Change%E2%80%9D+OR+mh%3A+%E2%80%9CMicroclimate%E2%80%9D+OR+mh%3A+%E2%80%9CClimate%E2%80%9D+OR+%E2%80%9CUrban+heat%E2%80%9D+OR+%E2%80%9Cheat+stress%E2%80%9D+OR+%E2%80%9CClimatic+change%E2%80%9D+OR+%E2%80%9CHeat+radiation%E2%80%9D+OR+%E2%80%9CTemperature+rise%E2%80%9D+OR+%E2%80%9CHeat+waves%E2%80%9D+OR+%E2%80%9CHeat+cramps%E2%80%9D+OR+%E2%80%9CHeat+collapse%E2%80%9D+OR+%E2%80%9CHeat+illness%E2%80%9D+OR+%E2%80%9CHeat+injury%E2%80%9D+OR+%E2%80%9CHeat-related+illness%E2%80%9D+OR+%E2%80%9CHeat+stress%E2%80%9D+OR+%E2%80%9CHeat+stress+disorder%E2%80%9D+OR+%E2%80%9CHeat+stress+syndrome%E2%80%9D+OR+%E2%80%9CHeat+syncope%E2%80%9D+OR+%E2%80%9CHeat+stroke%E2%80%9D+OR+%E2%80%9CThermal+strain%E2%80%9D+OR+%E2%80%9CThermal+stress%E2%80%9D+OR+%E2%80%9CUncompensable+heat+stress%E2%80%9D+OR+%E2%80%9CHeat+strain%E2%80%9D+OR+%E2%80%9CExtreme+heat%29+AND+%28Cooling+OR+mh%3A+%E2%80%9CGastric+hypothermia%E2%80%9D+OR+mh%3A+%E2%80%9CVentilation%E2%80%9D+OR+Relectiv*+OR+Communi*++OR+%22meteorology%22+OR+%22air+conditioning%22+OR+%22ergonomics%22+OR+%22Facility+Design+and+Construction%22+OR+%22thermosensing%22+OR+%22organizational+efficiency%22+OR+%22heat+health+prevention%22+OR+%22misting%22+OR+%22active+outreach%22+OR+%22healthcare+prepar*%22+OR+%22Green+planting%22+OR+%22renewable+energy%22+OR+%22fans%22+OR+%22refrigerators%22+OR+%22ice%22+OR+%22capacity+building%22+OR+%22heat+management%22+OR+%22Reflective+paint*%22+OR+%22Albedo%22+OR+%22Artificial+intelligence%22+OR+%22Trees%22+OR+%22Vegetation%22+OR+%22Passive+cooling%22+OR+%22reflectivity%22+OR+%22Water+spray%22+OR+%22Wet+Sheets%22+OR+%22ice-water%22+OR+%22Cooling+blanket%22+OR+%22Water+baths%22+OR+%22Community+mobilization%22+OR+%22Clothing%22+OR+%22Light+clothing%22+OR+%22Cooling%22+OR+%22Heat+%28stress%29+management%22+OR+%22Multimodal+interventions%22+OR+%22Community+care+programmes%22+OR+%22early+warning+systems%22+OR+%22community-led+initiatives%22+OR+%22effective+communication%22+OR+%22Cooling+vests%22+OR+%22Cooling+jackets%22+OR+%22Cold+shower%22+OR+%22Ice+sheet*%22+OR+%22cooling+devices%22+OR+%22reflective+blankets%22+OR+%22evaporative+cooling%22+OR+%22ice+packs%22+OR+%22cold+water+immersion%22+OR+%22intravenous+fluids%22+OR+%22cooling+vest%22+OR+%22Community-Based+Interventions%22+OR+%22heat+monitoring%22+OR+%22intranasal+cooling%22+OR+%22Neighborhood+Interventions%22+OR+%22Cool+coatings%22%29&where=&filter%5Bdb%5D%5B%5D=LILACS&range_year_start=&range_year_end=](https://pesquisa.bvsalud.org/portal/?u_filter%5B%5D=fulltext&u_filter%5B%5D=db&u_filter%5B%5D=mj_cluster&u_filter%5B%5D=type_of_study&u_filter%5B%5D=la&fb=&output=site&lang=en&from=1&sort=&format=summary&count=100&page=1&skfp=true&index=&q=%28mh%3A+%22Heat+Stroke%22+OR+mh%3A+%22Heat+Stress+Disorders%E2%80%9D+OR+mh%3A+%22Heat+Exhaustion%E2%80%9D+OR+mh%3A+%E2%80%9CClimate+Change%E2%80%9D+OR+mh%3A+%E2%80%9CMicroclimate%E2%80%9D+OR+mh%3A+%E2%80%9CClimate%E2%80%9D+OR+%E2%80%9CUrban+heat%E2%80%9D+OR+%E2%80%9Cheat+stress%E2%80%9D+OR+%E2%80%9CClimatic+change%E2%80%9D+OR+%E2%80%9CHeat+radiation%E2%80%9D+OR+%E2%80%9CTemperature+rise%E2%80%9D+OR+%E2%80%9CHeat+waves%E2%80%9D+OR+%E2%80%9CHeat+cramps%E2%80%9D+OR+%E2%80%9CHeat+collapse%E2%80%9D+OR+%E2%80%9CHeat+illness%E2%80%9D+OR+%E2%80%9CHeat+injury%E2%80%9D+OR+%E2%80%9CHeat-related+illness%E2%80%9D+OR+%E2%80%9CHeat+stress%E2%80%9D+OR+%E2%80%9CHeat+stress+disorder%E2%80%9D+OR+%E2%80%9CHeat+stress+syndrome%E2%80%9D+OR+%E2%80%9CHeat+syncope%E2%80%9D+OR+%E2%80%9CHeat+stroke%E2%80%9D+OR+%E2%80%9CThermal+strain%E2%80%9D+OR+%E2%80%9CThermal+stress%E2%80%9D+OR+%E2%80%9CUncompensable+heat+stress%E2%80%9D+OR+%E2%80%9CHeat+strain%E2%80%9D+OR+%E2%80%9CExtreme+heat%29+AND+%28Cooling+OR+mh%3A+%E2%80%9CGastric+hypothermia%E2%80%9D+OR+mh%3A+%E2%80%9CVentilation%E2%80%9D+OR+Relectiv*+OR+Communi*++OR+%22meteorology%22+OR+%22air+conditioning%22+OR+%22ergonomics%22+OR+%22Facility+Design+and+Construction%22+OR+%22thermosensing%22+OR+%22organizational+efficiency%22+OR+%22heat+health+prevention%22+OR+%22misting%22+OR+%22active+outreach%22+OR+%22healthcare+prepar*%22+OR+%22Green+planting%22+OR+%22renewable+energy%22+OR+%22fans%22+OR+%22refrigerators%22+OR+%22ice%22+OR+%22capacity+building%22+OR+%22heat+management%22+OR+%22Reflective+paint*%22+OR+%22Albedo%22+OR+%22Artificial+intelligence%22+OR+%22Trees%22+OR+%22Vegetation%22+OR+%22Passive+cooling%22+OR+%22reflectivity%22+OR+%22Water+spray%22+OR+%22Wet+Sheets%22+OR+%22ice-water%22+OR+%22Cooling+blanket%22+OR+%22Water+baths%22+OR+%22Community+mobilization%22+OR+%22Clothing%22+OR+%22Lig)

<https://web.s.ebscohost.com/ehost/search/basic?vid=7&sid=8586f60b-6238-4032-a56e-8bc381f624f4%40redis>
