# Supplementary material for: Evaluation of community-based heat adaptation interventions: a systematic review
Source: BMJ Public Health. 2025 Jul 15;3(2):e002332. doi: 10.1136/bmjph-2024-002332 (PMC12273142; doi:10.1136/bmjph-2024-002332)

ANNEX 3 - Meta-analysis for Surface Temperature

Interventions	Interventions Mean	SD	N	Control Mean	SD	N	Weight	MD	95% CI
**Green Roof vs Conventional Roof**									
Green Roof {Baryla 2019}	23.42	6.8441	6	43.83	15.1581	6	3.3%	-20.42	[-33.72; -7.11]
Green Roof {Jaffal 2012}	10.93	7.3433	15	19.40	18.8065	15	3.8%	-8.47	[-18.68; 1.75]
Green Roof {Kumar 2016}	21.44	0.6877	5	24.12	2.5801	5	4.8%	-2.68	[-5.02; -0.34]
Green Roof {Lee 2019}	29.49	1.2710	8	32.33	4.2176	8	4.7%	-2.84	[-5.89; 0.21]
Green Roof {Ouldboukhitine 2013}	13.91	2.8749	23	36.35	16.9347	23	4.3%	-22.43	[-29.45; -15.41]
Green Roof {Rahman 2022}	22.87	5.9214	3	46.70	2.4249	3	4.2%	-23.83	[-31.07; -16.59]
Green Roof {Tseng 2022}	32.67	6.5192	9	38.33	10.4403	9	4.1%	-5.67	[-13.71; 2.37]
Green Roof A {Saify 2023}	31.50	2.6339	9	48.50	7.7822	9	4.5%	-17.00	[-22.37; -11.63]
Green Roof B {Saify 2023}	30.56	2.3776	9	44.50	5.7337	9	4.6%	-13.94	[-18.00; -9.89]
Green Roof - Stone Crop {Seo 2023}	25.62	0.9161	8	26.88	1.1260	8	4.8%	-1.25	[-2.26; -0.24]
Green Roof - Grass {Seo 2023}	27.88	0.6409	8	26.88	1.1260	8	4.8%	1.00	[0.10; 1.90]
Green Roof - 21.7cm {Bevilacqua 2017}	44.13	3.9221	31	67.81	5.0492	31	4.8%	-23.68	[-25.93; -21.43]
Green Roof - 20.6cm {Bevilacqua 2017}	40.77	3.5657	31	67.81	5.0492	31	4.8%	-27.03	[-29.21; -24.86]
Green Roof - 24.5cm {Bevilacqua 2017}	38.98	1.9170	31	67.81	5.0492	31	4.8%	-28.82	[-30.72; -26.92]
Extensive Green Roof {Tang 2019}	27.90	1.3416	5	33.40	6.5803	5	4.4%	-5.50	[-11.39; 0.39]
Extensive Green Roof - Taipei {Lin 2013}	22.98	4.5417	5	39.75	9.2536	5	4.0%	-16.77	[-25.81; -7.73]
Extensive Green Roof - Chiayi {Lin 2013}	26.40	4.5144	5	45.04	9.0966	5	4.0%	-18.64	[-27.54; -9.74]
Non-irrigated Modular Green Roofs {Dvorak 2013}	35.00	12.5512	31	39.29	19.2409	31	4.1%	-4.29	[-12.38; 3.80]
Multilayer Green Roof {Pumo 2023}	19.00	11.5109	5	21.40	12.9954	5	3.0%	-2.40	[-17.62; 12.82]
Green Blue Roof {Shafique 2017}	22.19	8.2802	29	24.54	6.9122	29	4.6%	-2.35	[-6.27; 1.58]
Tree Canopy {Jim 2011}	30.22	3.4053	32	31.23	0.4576	32	4.8%	-1.02	[-2.21; 0.17]
Green Energy Roof {Tseng 2022}	33.11	6.7536	9	38.33	10.4403	9	4.1%	-5.22	[-13.35; 2.90]
Wetland {Song 2013}	23.17	2.4221	6	21.67	4.2308	6	4.6%	1.50	[-2.40; 5.40]
**Total (95% CI)**			**323**			**323**	**100.0%**	**-10.88**	**[-15.26; -6.50]**

Heterogeneity: $Tau^2 = 96.3337$; $Chi^2 = 1653.56$, df = 22 (P = 0); $I^2 = 99\%$

Mean Difference
IV, Random, 95% CI

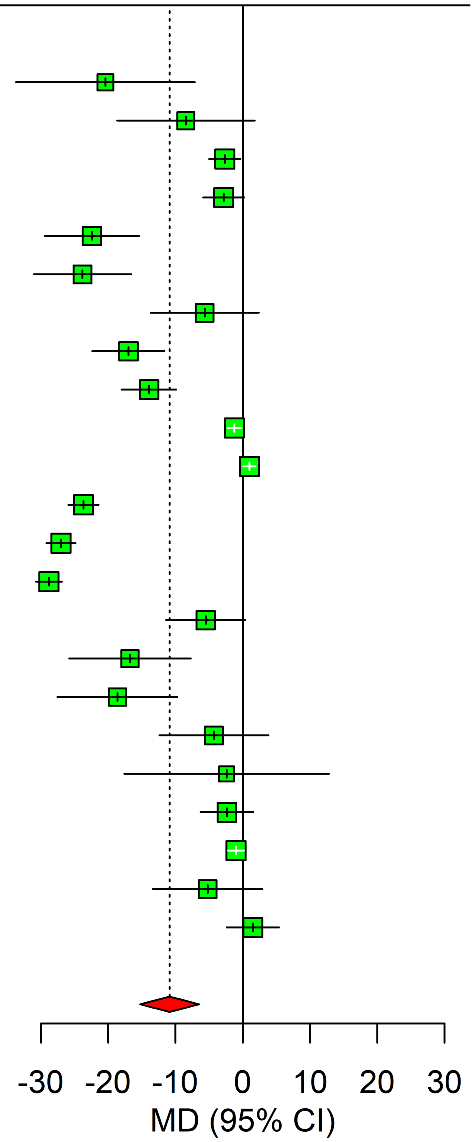

Interventions	Interventions Mean	Interventions SD	N	Control Mean	Control SD	N	Weight	MD	95% CI
**Green Roof vs Bare substrate**									
Modular Green Roof - Sibbaldiopsis tridentata {MacIvor 2011}	33.48	0.7400	5	34.08	0.4600	5	2.7%	-0.60	[-1.36; 0.16]
Modular Green Roof - Danthonia Spicata {MacIvor 2011}	32.16	0.7400	5	34.08	0.4600	5	2.7%	-1.92	[-2.68; -1.16]
Modular Green Roof - Empetrum Nigrum {MacIvor 2011}	32.54	0.8000	5	34.08	0.4600	5	2.6%	-1.54	[-2.35; -0.73]
Modular Green Roof - Kalmia Polifolia {MacIvor 2011}	34.14	0.7400	5	34.08	0.4600	5	2.7%	0.06	[-0.70; 0.82]
Modular Green Roof - Scirpus Cespitosus {MacIvor 2011}	33.54	0.7400	5	34.08	0.4600	5	2.7%	-0.54	[-1.30; 0.22]
Modular Green Roof - Vaccinium Macrocarpan {MacIvor 2011}	33.12	0.7400	5	34.08	0.4600	5	2.7%	-0.96	[-1.72; -0.20]
Modular Green Roof - Sibbaldiopsis tridentata, Danthonia Spicata {MacIvor 2011}	31.50	0.7400	5	34.08	0.4600	5	2.7%	-2.58	[-3.34; -1.82]
Modular Green Roof - Sibbaldiopsis tridentata, Empetrum Nigrum {MacIvor 2011}	31.86	0.7400	5	34.08	0.4600	5	2.7%	-2.22	[-2.98; -1.46]
Modular Green Roof - Danthonia Spicata, Empetrum Nigrum {MacIvor 2011}	32.06	0.7400	5	34.08	0.4600	5	2.7%	-2.02	[-2.78; -1.26]
Modular Green Roof - Sibbaldiopsis, Danthonia Spicata, Empetrum Nigrum {MacIvor 2011}	32.07	0.6000	10	34.08	0.4600	10	3.4%	-2.01	[-2.48; -1.54]
Modular Green Roof - Scirpus Cespitosus, Vaccinium Macrocarpan {MacIvor 2011}	32.41	0.8000	5	34.08	0.4600	5	2.6%	-1.67	[-2.48; -0.86]
Modular Green Roof - Kalmia Polifolia, Vaccinium Macrocarpan {MacIvor 2011}	32.84	0.7400	5	34.08	0.4600	5	2.7%	-1.24	[-2.00; -0.48]
Modular Green Roof - Kalmia Polifolia, Scirpus Cespitosus {MacIvor 2011}	33.48	0.7400	5	34.08	0.4600	5	2.7%	-0.60	[-1.36; 0.16]
Modular Green Roof - Kalmia Polifolia, Scirpus Cespitosus, Vaccinium Macrocarpan {MacIvor 2011}	34.03	0.6000	10	34.08	0.4600	10	3.4%	-0.05	[-0.52; 0.42]
Modular Green Roof - Sibbaldiopsis Tridentata, Kalmia Polifolia {MacIvor 2011}	32.26	0.7400	5	34.08	0.4600	5	2.7%	-1.82	[-2.58; -1.06]
Modular Green Roof - Danthonia Spicata, Kalmia Polifolia {MacIvor 2011}	32.94	0.7400	5	34.08	0.4600	5	2.7%	-1.14	[-1.90; -0.38]
Modular Green Roof - Empetrum Nigrum, Kalmia Polifolia {MacIvor 2011}	34.32	0.8000	5	34.08	0.4600	5	2.6%	0.24	[-0.57; 1.05]
Modular Green Roof - Sibbaldiopsis Tridentata, Scirpus Cespitosus {MacIvor 2011}	33.16	0.7400	5	34.08	0.4600	5	2.7%	-0.92	[-1.68; -0.16]
Modular Green Roof - Danthonia Spicata, Scirpus Cespitosus {MacIvor 2011}	32.71	0.8000	5	34.08	0.4600	5	2.6%	-1.37	[-2.18; -0.56]
Modular Green Roof - Empetrum Nigrum, Scirpus Cespitosus {MacIvor 2011}	32.52	0.7400	5	34.08	0.4600	5	2.7%	-1.56	[-2.32; -0.80]
Modular Green Roof - Sibbaldiopsis Tridentata, Vaccinium Macrocarpan {MacIvor 2011}	33.66	0.7400	5	34.08	0.4600	5	2.7%	-0.42	[-1.18; 0.34]
Modular Green Roof - Danthonia Spicata, Vaccinium Macrocarpan {MacIvor 2011}	33.24	0.7400	5	34.08	0.4600	5	2.7%	-0.84	[-1.60; -0.08]
Modular Green Roof - Empetrum Nigrum, Vaccinium Macrocarpan {MacIvor 2011}	33.04	0.7400	5	34.08	0.4600	5	2.7%	-1.04	[-1.80; -0.28]
Modular Green Roof - Danthonia Spicata, Empertrum Nigrum, Scirpus Cespitosus, Vaccinium Macrocarpan {MacIvor 2011}	32.88	0.7400	5	34.08	0.4600	5	2.7%	-1.20	[-1.96; -0.44]
Modular Green Roof - Danthonia Spicata, Sibbaldiopsis Tridentata, Kalmia Polifolia, Scirpus Cespitosus {MacIvor 2011}	32.30	0.7400	5	34.08	0.4600	5	2.7%	-1.78	[-2.54; -1.02]
Modular Green Roof - Danthonia Spicata, Sibbaldiopsis Tridentata, Scirpus Cespitosus, Vaccinium Macrocarpan {MacIvor 2011}	32.79	0.8000	5	34.08	0.4600	5	2.6%	-1.29	[-2.10; -0.48]
Modular Green Roof - Danthonia Spicata, Sibbaldiopsis Tridentata, Vaccinium Macrocarpan, Kalmia Polifolia {MacIvor 2011}	32.60	0.7400	5	34.08	0.4600	5	2.7%	-1.48	[-2.24; -0.72]
Modular Green Roof - Danthonia Spicata, Empertrum Nigrum, Kalmia Polifolia, Vaccinium Macrocarpan {MacIvor 2011}	32.16	0.7400	5	34.08	0.4600	5	2.7%	-1.92	[-2.68; -1.16]
Modular Green Roof - Sibbaldiopsis Tridentata, Empertrum Nigrum, Kalmia Polifolia, Scirpus Cespitosus {MacIvor 2011}	33.62	0.7400	5	34.08	0.4600	5	2.7%	-0.46	[-1.22; 0.30]
Modular Green Roof - Sibbaldiopsis Tridentata, Empertrum Nigrum, Kalmia Polifolia, Scirpus Cespitosus {MacIvor 2011}	33.04	0.7400	5	34.08	0.4600	5	2.7%	-1.04	[-1.80; -0.28]
Modular Green Roof - Danthonia Spicata, Empertrum Nigrum, Kalmia Polifolia, Scirpus Cespitosus {MacIvor 2011}	34.20	0.7400	5	34.08	0.4600	5	2.7%	0.12	[-0.64; 0.88]
Modular Green Roof - Sibbaldiopsis Tridentata, Empertrum Nigrum, Kalmia Polifolia, Scirpus Cespitosus {MacIvor 2011}	33.38	0.7400	5	34.08	0.4600	5	2.7%	-0.70	[-1.46; 0.06]
Modular Green Roof - Sibbaldiopsis Tridentata, Danthonia Spicata, Empertrum Nigrum, Kalmia Polifolia, Scirpus Cespitosus, Vaccinium Macrocarpan {MacIvor 2011}	32.76	0.6000	10	34.08	0.4600	10	3.4%	-1.32	[-1.79; -0.85]
Green Roof - Heuchera purple {Monteiro 2017}	14.80	5.1672	17	15.00	2.2361	17	0.6%	-0.20	[-2.88; 2.48]
Green Roof - Heuchera yellow {Monteiro 2017}	13.20	1.0954	17	15.00	2.2361	17	1.9%	-1.80	[-2.98; -0.62]
Green Roof - Salvia {Monteiro 2017}	13.80	0.8367	17	15.00	2.2361	17	2.0%	-1.20	[-2.33; -0.07]
Green Roof - Stachys {Monteiro 2017}	13.40	2.0736	17	15.00	2.2361	17	1.5%	-1.60	[-3.05; -0.15]
Green Roof - Sedum {Monteiro 2017}	13.80	1.7889	17	15.00	2.2361	17	1.6%	-1.20	[-2.56; 0.16]
Green Roof - Sempervivum {Monteiro 2017}	14.00	2.2361	17	15.00	2.2361	17	1.4%	-1.00	[-2.50; 0.50]
**Total (95% CI)**			**282**			**282**	**100.0%**	**-1.16**	**[-1.38; -0.94]**

Heterogeneity: $Tau^2 = 0.3322$; $Chi^2 = 128.22$, df = 38 ($P < 0.01$); $I^2 = 70\%$

Mean Difference
IV, Random, 95% CI

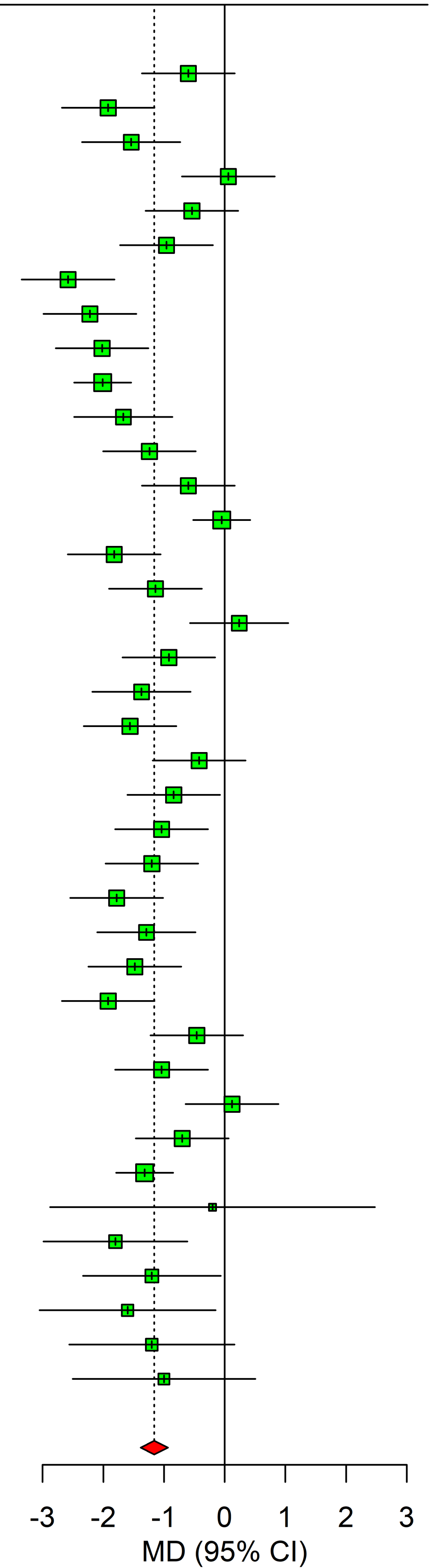

Interventions	Interventions Mean	SD	N	Control Mean	SD	N	Weight	MD	95% CI
**Green Roof vs Wood Roof**									
Green Roof {Zhang 2021}	23.82	4.1418	17	25.35	8.5801	17	7.5%	-1.53	[-6.06; 3.00]
Sand substrate {Sisco 2017}	32.43	1.8866	15	33.30	2.9020	15	50.1%	-0.87	[-2.62; 0.88]
Pellet substrate {Sisco 2017}	33.20	2.3964	15	33.30	2.9020	15	42.4%	-0.10	[-2.00; 1.80]
**Total (95% CI)**			**47**			**47**	**100.0%**	**-0.59**	**[-1.98; 0.79]**

Heterogeneity: $Tau^2 = 0$; $Chi^2 = 0.52$, df = 2 (P = 0.77); $I^2 = 0\%$

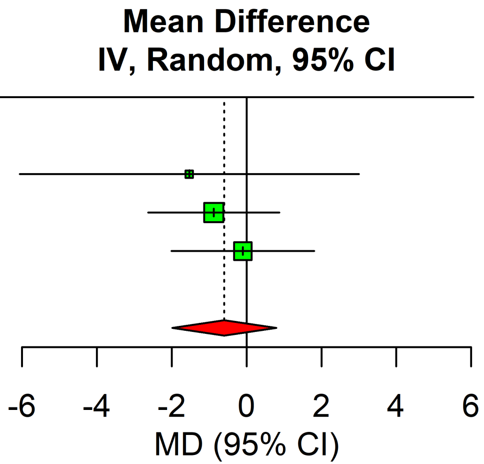

Interventions	Interventions Mean	SD	N	Control Mean	SD	N	Weight	MD	95% CI
**Green Roof vs Black Roof**									
Green Roof {Pompeii II 2010}	20.83	3.6584	24	21.17	3.7290	24	26.6%	-0.33	[-2.42; 1.76]
**Green Roof vs White Gravel Roof**									
Green Roof {Solcerova 2017}	23.44	5.5817	18	24.92	10.4322	18	16.3%	-1.48	[-6.94; 3.99]
**Green Roof vs White Roof**									
Extensive Vegetated Roof {Robbiati 2022}	27.68	3.3000	120	35.11	4.7400	120	29.0%	-7.43	[-8.46; -6.40]
**Green Roof vs Green Paint**									
Green Roof - Poa Pratensis {Lee 2015}	29.08	5.0042	6	32.00	6.3797	6	13.7%	-2.92	[-9.40; 3.57]
Green Roof - Sedum Sarmentosum {Lee 2015}	29.25	4.4357	6	32.00	6.3797	6	14.3%	-2.75	[-8.97; 3.47]
**Total (95% CI)**			**12**			**12**	**28.1%**	**-2.83**	**[-3.89; -1.77]**
Heterogeneity: $Tau^2 = 0$; $Chi^2 = 0$, df = 1 (P = 0.97); $I^2 = 0\%$									
**Total (95% CI)**			**174**			**174**	**100.0%**	**-3.28**	**[-7.18; 0.62]**
Heterogeneity: $Tau^2 = 9.2822$; $Chi^2 = 39.92$, df = 4 (P < 0.01); $I^2 = 90\%$									
Test for subgroup differences: $Chi^2 = 80.49$, df = 3 (P < 0.01)									

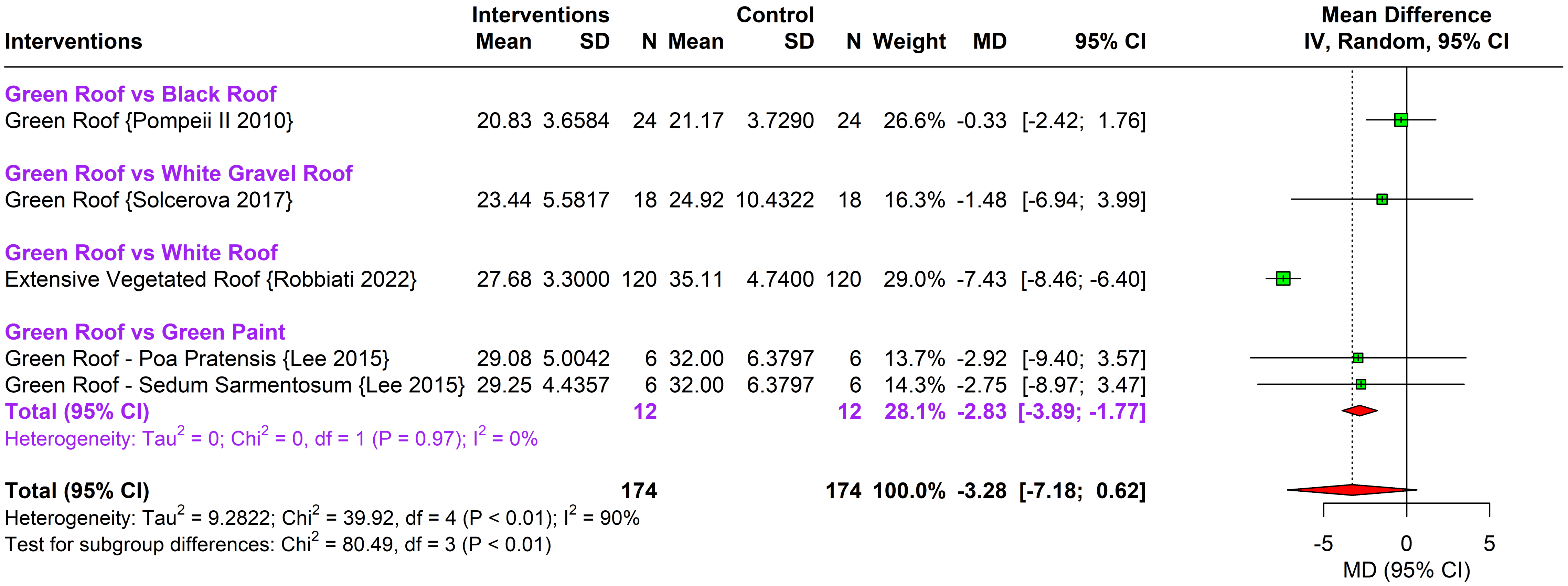

Interventions	Interventions Mean	Interventions SD	Interventions N	Control Mean	Control SD	Control N	Weight	MD	95% CI	Mean Difference IV, Random, 95% CI
**Green Roof vs Non-reflective (EPDM) roof membrane**										
Modular Green Roof - Spurium-Arkalyte {Celik 2010}	32.41	6.2483	11	34.55	9.9435	11	10.9%	-2.14	[-9.08; 4.80]	
Modular Green Roof - Spurium-Lava {Celik 2010}	31.55	5.3407	11	34.55	9.9435	11	11.8%	-3.00	[-9.67; 3.67]	
Modular Green Roof - Spurium-Hadite {Celik 2010}	33.77	6.4706	11	34.55	9.9435	11	10.7%	-0.77	[-7.78; 6.24]	
Modular Green Roof - Kamtchaticum-Arkalyte {Celik 2010}	32.82	6.2581	11	34.55	9.9435	11	10.9%	-1.73	[-8.67; 5.22]	
Modular Green Roof - Kamtchaticum-Lava {Celik 2010}	32.55	5.9810	11	34.55	9.9435	11	11.1%	-2.00	[-8.86; 4.86]	
Modular Green Roof - Kamtchaticum-Hadite {Celik 2010}	33.45	6.4593	11	34.55	9.9435	11	10.7%	-1.09	[-8.10; 5.92]	
Modular Green Roof - Sexangulare-Lava {Celik 2010}	33.00	6.7157	11	34.55	9.9435	11	10.4%	-1.55	[-8.64; 5.55]	
Modular Green Roof - Sexangulare-Arkalyte {Celik 2010}	32.64	5.4868	11	34.55	9.9435	11	11.6%	-1.91	[-8.62; 4.80]	
Modular Green Roof - Sexangulare-Hadite {Celik 2010}	32.91	5.0932	11	34.55	9.9435	11	12.0%	-1.64	[-8.24; 4.97]	
**Total (95% CI)**			**99**			**99**	**100.0%**	**-1.77**	**[-2.27; -1.28]**	

Heterogeneity: $Tau^2 = 0$; $Chi^2 = 0.27$, df = 8 (P = 1.00); $I^2 = 0\%$

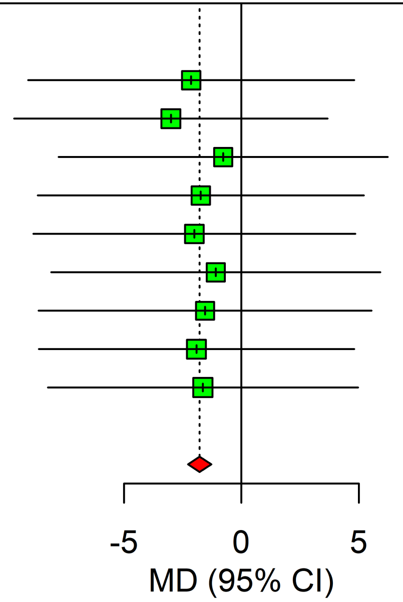

Interventions	Interventions Mean	Interventions SD	Interventions N	Control Mean	Control SD	Control N	Weight	MD	95% CI
**Green Facade vs Bare wall**									
Green Facade {Safikhani 2014}	29.27	4.3900	2	29.52	7.3200	2	1.1%	-0.25	[-12.08; 11.58]
Green Facade {Widiastuti 2018}	28.25	3.8500	14	31.96	1.8600	14	2.6%	-3.71	[-5.95; -1.47]
Green Facade {Susorova 2013}	25.04	1.0300	12	25.33	1.1500	12	2.7%	-0.29	[-1.16; 0.58]
Green Facade {Susorova 2014}	28.30	3.5000	12481	29.50	5.9000	12481	2.7%	-1.20	[-1.32; -1.08]
Green Facade {Yin 2017}	36.54	2.4500	24	39.60	4.3300	24	2.6%	-3.06	[-5.05; -1.07]
Green Facade {Zhen 2022}	29.70	1.4700	10	33.60	2.1800	10	2.7%	-3.90	[-5.53; -2.27]
Green Facade {Jaafar 2015}	30.01	2.7930	240	30.63	5.9480	240	2.7%	-0.61	[-1.45; 0.22]
Green Facade {Widiastuti 2018}	29.00	2.1500	14	31.96	1.8600	14	2.7%	-2.96	[-4.45; -1.47]
Green Facade {Saify 2023}	37.33	3.8649	9	44.44	5.3470	9	2.3%	-7.11	[-11.42; -2.80]
Green Facade {Pichlhöfer 2023}	26.30	0.8000	21	27.50	0.9000	21	2.7%	-1.20	[-1.72; -0.68]
Direct Green Facade {Perini 2011}	10.39	2.2100	10	11.59	2.4100	10	2.6%	-1.20	[-3.23; 0.83]
Indirect Green Facade {Suklje 2016}	29.00	4.7200	8	41.38	9.8600	8	1.7%	-12.38	[-19.96; -4.80]
Indirect Green Facade {Lin 2019}	31.80	1.7200	10	32.25	1.8300	10	2.7%	-0.45	[-2.01; 1.11]
Indirect Green Facade {Perini 2011}	10.50	2.0500	10	13.23	2.2800	10	2.6%	-2.73	[-4.63; -0.83]
Double-skin Green Facade {Perez 2017}	18.72	5.2800	39	21.69	11.4500	39	2.3%	-2.97	[-6.93; 0.99]
Double-skin Green Facade {Coma 2017}	10.62	14.9500	13	10.04	16.6600	13	1.0%	0.58	[-11.59; 12.75]
Plant-shaded facades {Haggag 2017}	44.53	2.2900	15	48.07	6.0600	15	2.4%	-3.54	[-6.82; -0.26]
**Total (95% CI)**			**12932**			**12932**	**40.2%**	**-2.12**	**[-3.10; -1.14]**
Heterogeneity: $Tau^2 = 1.6093$; $Chi^2 = 51.84$, df = 16 ($P < 0.01$); $I^2 = 69\%$									
**Living Wall vs Bare wall**									
Living Wall {Chen 2013}	32.88	2.0000	17	36.65	7.4500	17	2.4%	-3.77	[-7.44; -0.10]
Living Wall {He 2017}	29.96	1.7100	13	37.08	9.6400	13	2.1%	-7.12	[-12.44; -1.80]
Living Wall {Safikhani 2014}	28.96	4.0100	2	29.52	7.3200	2	1.1%	-0.56	[-12.13; 11.01]
Living Wall {Tudiwer 2017}	16.14	3.5800	7	25.93	4.5400	7	2.3%	-9.79	[-14.07; -5.51]
Living Wall {Wang 2011}	30.70	1.8500	22	32.69	5.0700	22	2.6%	-1.99	[-4.25; 0.27]
Living Wall {Wong 2010}	28.59	0.7000	11	30.50	8.7000	11	2.1%	-1.91	[-7.07; 3.25]
Living Wall {Haggag 2014}	49.39	0.4300	7	55.96	0.4700	7	2.7%	-6.57	[-7.04; -6.10]
Living Wall {Olivieri 2014}	21.34	2.4100	53	34.43	3.4700	53	2.7%	-13.09	[-14.23; -11.95]
Living Wall {Coma 2017}	0.85	2.3700	13	10.04	16.6600	13	1.4%	-9.19	[-18.34; -0.04]
Living Wall {Djedjig 2015}	23.33	1.9200	9	23.61	2.7000	9	2.6%	-0.28	[-2.44; 1.88]
Living Wall {Shah 2023}	32.75	2.2546	4	35.38	1.2500	4	2.6%	-2.62	[-5.15; -0.10]
Living Wall - Festuca Glauca {Cortes 2022}	24.23	7.5000	26	23.75	6.0200	26	2.4%	0.48	[-3.22; 4.18]
Living Wall - Thymus Pulegioides {Cortes 2022}	24.76	8.2700	26	25.69	8.8000	26	2.2%	-0.93	[-5.57; 3.71]
Living Wall - Festuca Glauca {Cortes 2022}	24.23	7.5000	26	25.69	8.8000	26	2.2%	-1.46	[-5.90; 2.98]
Vertical Greenery Module System {Bianco 2016}	24.92	4.0800	13	37.42	14.4100	13	1.6%	-12.50	[-20.64; -4.36]
Green Module Wall {Gouws 2022}	22.76	2.4800	31	26.98	3.6500	31	2.7%	-4.22	[-5.77; -2.67]
Modular System {Jaafar 2015}	29.84	2.1830	240	30.63	5.9480	240	2.7%	-0.79	[-1.59; 0.02]
Geogreen module {Manso 2016}	18.72	1.7200	82	19.29	2.1000	82	2.7%	-0.57	[-1.16; 0.02]
Modular System - Nitida Lemon {Sudimac 2018}	16.28	3.4100	14	8.07	3.8300	14	2.5%	8.21	[5.52; 10.90]
Modular System - Geranium Macrorrhiuum {Sudimac 2018}	23.28	4.4800	14	8.07	3.8300	14	2.5%	15.21	[12.12; 18.30]
Modular System - Cordifolia Stock {Sudimac 2018}	17.00	6.2300	14	8.07	3.8300	14	2.4%	8.93	[5.10; 12.76]
Hanging potted pothos {Huang 2018}	30.20	3.7900	51	31.04	5.3800	51	2.6%	-0.84	[-2.65; 0.97]
Vertical Garden {Nugroho 2020}	29.64	3.5900	5	46.04	9.8000	5	1.4%	-16.40	[-25.55; -7.25]
Cork-based Living Wall {Cortes 2022}	24.76	8.2700	26	23.75	6.0200	26	2.3%	1.01	[-2.92; 4.94]
Continuous Living Wall {Cruciol-Barbosa 2023}	30.12	3.1100	56	32.50	2.9000	56	2.7%	-2.38	[-3.49; -1.27]
Felt layer {Nori 2013}	20.56	2.4000	24	25.60	10.9500	24	2.2%	-5.04	[-9.52; -0.56]
**Total (95% CI)**			**806**			**806**	**59.8%**	**-2.29**	**[-4.99; 0.40]**
Heterogeneity: $Tau^2 = 38.8103$; $Chi^2 = 889.22$, df = 25 ($P < 0.01$); $I^2 = 97\%$									
**Total (95% CI)**			**13738**			**13738**	**100.0%**	**-2.39**	**[-4.03; -0.74]**
Heterogeneity: $Tau^2 = 23.5085$; $Chi^2 = 1182.20$, df = 42 ($P < 0.01$); $I^2 = 96\%$									
Test for subgroup differences: $Chi^2 = 0.02$, df = 1 ($P = 0.90$)									

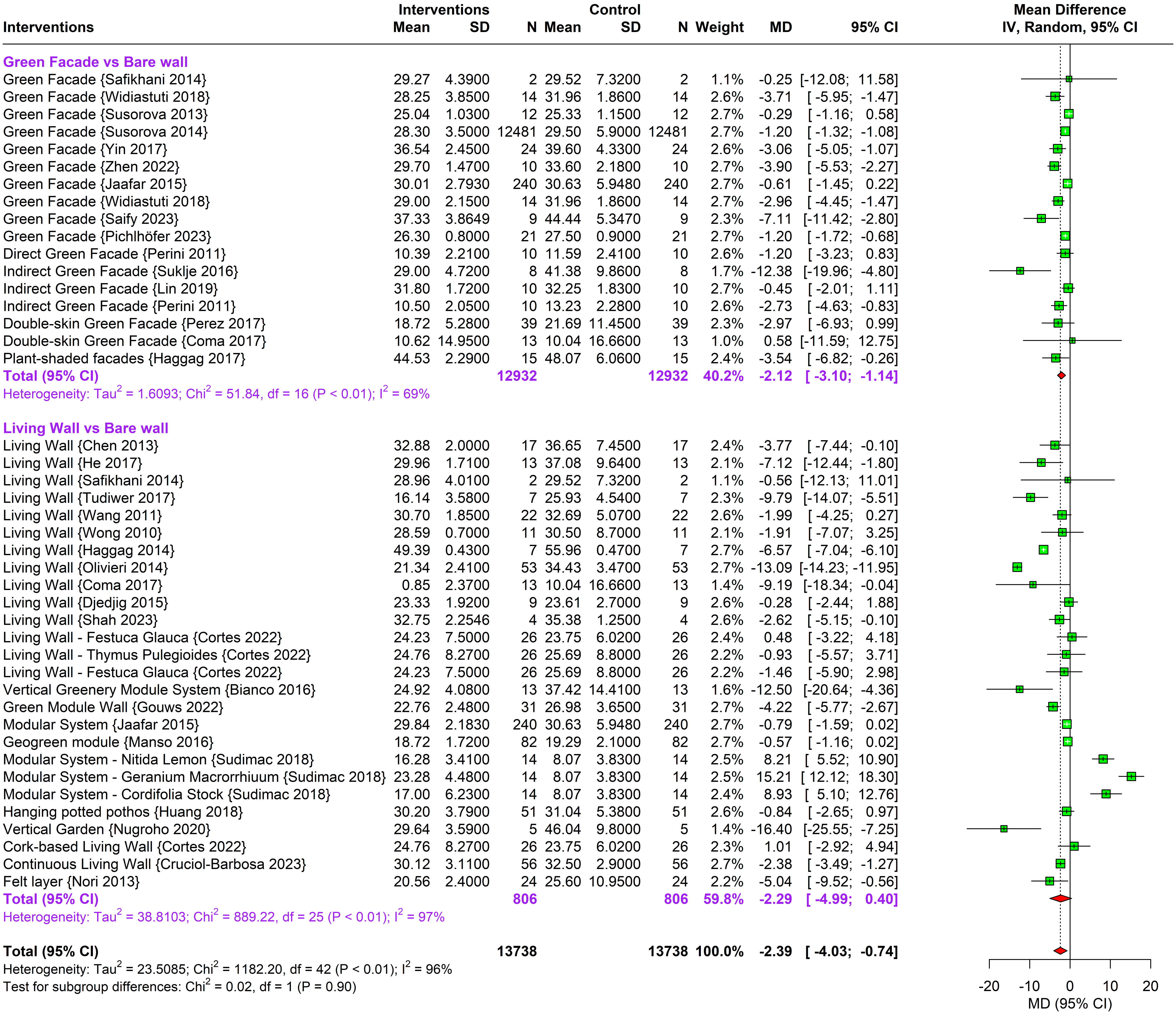

Interventions	Interventions Mean	SD	N	Control Mean	SD	N	Weight	MD	95% CI
**Green Facade vs Bricked wall**									
Green Facade {Bakhshoodeh 2022}	25.95	2.5159	10	27.63	4.4056	10	3.6%	-1.68	[-4.82; 1.47]
Green Facade {Benhalilou 2020}	27.23	1.6600	11	31.59	5.5100	11	3.2%	-4.36	[-7.76; -0.96]
Green Facade {Cuce 2017}	6.80	2.8500	102	10.86	3.0800	102	10.8%	-4.06	[-4.87; -3.25]
Direct Green Facade {Kokogiannakis 2019}	29.12	1.7700	3050	30.62	2.4800	3050	12.6%	-1.50	[-1.61; -1.39]
Indirect Green Facade {Kokogiannakis 2019}	29.50	1.9800	3050	30.62	2.4800	3050	12.6%	-1.12	[-1.23; -1.01]
Plant-shaded facades {Cameron 2014}	17.88	2.4800	17	18.74	3.1100	17	6.6%	-0.86	[-2.75; 1.03]
Green Facade {Pichlhöfer 2023}	25.00	0.5000	17	26.30	0.2000	17	12.5%	-1.30	[-1.56; -1.04]
Green Facade {Pichlhöfer 2023}	24.70	0.6000	17	26.30	0.2000	17	12.4%	-1.60	[-1.90; -1.30]
**Total (95% CI)**			**6274**			**6274**	**74.2%**	**-1.87**	**[-2.87; -0.87]**
Heterogeneity: $Tau^2 = 1.0594$; $Chi^2 = 72.05$, df = 7 ($P < 0.01$); $I^2 = 90\%$									
**Living Wall vs Bricked wall**									
Living Wall {Razzaghmanesh 2017}	23.47	4.9500	3	26.00	7.0900	3	0.5%	-2.53	[-12.31; 7.25]
Planter boxes {Kokogiannakis 2019}	27.50	0.9900	3050	30.62	2.4800	3050	12.6%	-3.12	[-3.21; -3.03]
Felt layer {Kokogiannakis 2019}	28.00	1.1300	3050	30.62	2.4800	3050	12.6%	-2.62	[-2.72; -2.52]
**Total (95% CI)**			**6103**			**6103**	**25.8%**	**-2.87**	**[-3.63; -2.11]**
Heterogeneity: $Tau^2 = 0.1217$; $Chi^2 = 52.38$, df = 2 ($P < 0.01$); $I^2 = 96\%$									
**Total (95% CI)**			**12377**			**12377**	**100.0%**	**-2.12**	**[-2.87; -1.37]**
Heterogeneity: $Tau^2 = 1.0040$; $Chi^2 = 1032.89$, df = 10 ($P < 0.01$); $I^2 = 99\%$									
Test for subgroup differences: $Chi^2 = 4.76$, df = 1 ($P = 0.03$)									

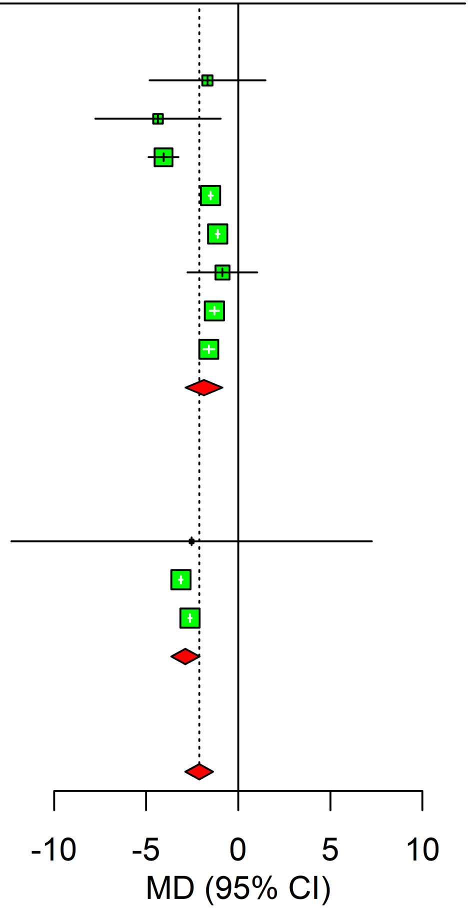

Interventions	Interventions Mean	SD	N	Control Mean	SD	N	Weight	MD	95% CI
**Green Facade vs Galvanized plate**									
Green Facade - Canavalia Gladiata {Koyama 2013}	20.28	2.4900	7	20.86	1.8600	7	16.1%	-0.58	[-2.88; 1.72]
Green Facade - Momordica Charantia {Koyama 2013}	20.71	2.0600	7	20.86	1.8600	7	20.1%	-0.15	[-2.21; 1.91]
Green Facade - Lpomoea Tricolor {Koyama 2013}	19.64	2.0600	7	20.86	1.8600	7	20.1%	-1.22	[-3.28; 0.84]
Green Facade - Pueraria Lobata {Koyama 2013}	20.61	1.9100	7	20.86	1.8600	7	21.8%	-0.25	[-2.22; 1.72]
Green Facade - Apios American {Koyama 2013}	20.64	1.9100	7	20.86	1.8600	7	21.8%	-0.22	[-2.19; 1.75]
**Total (95% CI)**			**35**			**35**	**100.0%**	**-0.47**	**[-1.03; 0.08]**

Heterogeneity: $Tau^2 = 0$; $Chi^2 = 0.72$, df = 4 (P = 0.95); $I^2 = 0\%$

Mean Difference
IV, Random, 95% CI

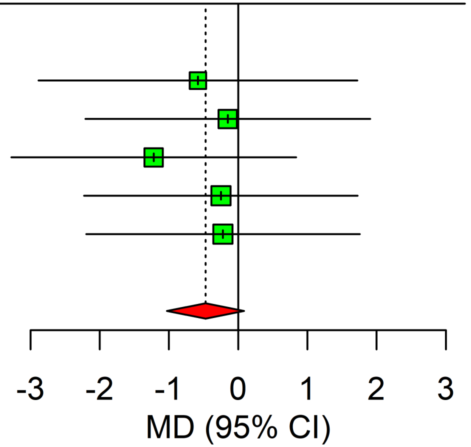

Interventions	Interventions Mean	SD	N	Control Mean	SD	N	Weight	MD	95% CI
**Modified Concrete vs Concrete**									
Concrete paver (PC) on concrete {Barišic´ 2022}	33.44	11.5401	18	30.61	9.1419	18	3.4%	2.83	[-3.97; 9.63]
Concrete paver (PC) on grass {Barišic´ 2022}	33.38	11.8764	20	30.20	9.1643	20	3.5%	3.18	[-3.40; 9.75]
Pervious/Porous concrete (P) on concrete {Barišic´ 2022}	33.22	10.9507	18	30.61	9.1419	18	3.5%	2.61	[-3.98; 9.20]
Pervious/Porous concrete (P) on grass {Barišic´ 2022}	33.55	11.1590	20	30.20	9.1643	20	3.6%	3.35	[-2.98; 9.68]
Pervious/Porous concrete (P)1 {Kevern 2012}	37.90	6.1290	24	35.50	4.7388	24	4.8%	2.40	[-0.70; 5.50]
Pervious/Porous concrete (P)2 {Takebayashi 2012}	35.22	8.3745	9	34.47	6.5715	9	3.3%	0.76	[-6.20; 7.71]
Water-retaining concrete, A {Takebayashi 2012}	34.61	8.7385	9	34.47	6.5715	9	3.3%	0.14	[-7.00; 7.29]
Water-retaining concrete, B {Takebayashi 2012}	35.78	8.8534	9	34.47	6.5715	9	3.2%	1.31	[-5.89; 8.51]
Interlocking block-porous {Takebayashi 2012}	33.61	7.5984	11	34.47	6.5715	11	3.7%	-0.86	[-6.79; 5.08]
Interlocking block water retaining {Takebayashi 2012}	34.17	7.4498	11	34.47	6.5715	11	3.7%	-0.30	[-6.17; 5.57]
Permeable concrete {Wang 2018}	33.00	9.7047	12	30.83	7.8721	12	3.3%	2.17	[-4.90; 9.24]
**Total (95% CI)**			**161**			**161**	**39.3%**	**1.74**	**[0.81; 2.68]**
Heterogeneity: $Tau^2 = 0$; $Chi^2 = 2.26$, df = 10 (P = 0.99); $I^2 = 0\%$									
**Bricks/Tiles/Pavers vs Concrete**									
Porous pavement 10% (PA1) {Ferrari 2020}	27.78	10.7823	9	27.94	10.1194	9	2.4%	-0.17	[-9.83; 9.49]
Porous pavement 20% (PA2) {Ferrari 2020}	27.67	11.4182	9	27.94	10.1194	9	2.4%	-0.28	[-10.25; 9.69]
Porous pavement 30% (PA3) {Ferrari 2020}	27.44	12.0687	9	27.94	10.1194	9	2.3%	-0.50	[-10.79; 9.79]
PA1-PA2 {Ferrari 2020}	26.06	10.1318	9	26.31	11.3639	9	2.4%	-0.26	[-10.20; 9.69]
PA1-PA3 {Ferrari 2020}	23.89	9.6946	9	26.31	11.3639	9	2.4%	-2.42	[-12.18; 7.34]
PA2-PA1 {Ferrari 2020}	26.31	11.3639	9	26.31	11.3639	9	2.2%	0.00	[-10.50; 10.50]
Cool pavement (Lime cement plaster) {Kolokotsa 2018}	33.49	2.8997	12	36.05	3.5482	12	5.0%	-2.56	[-5.15; 0.04]
Ceramic porous brick {Wang 2018}	29.33	9.1486	12	28.08	7.2796	12	3.5%	1.25	[-5.36; 7.86]
**Total (95% CI)**			**78**			**78**	**22.6%**	**-1.68**	**[-2.88; -0.48]**
Heterogeneity: $Tau^2 = 0$; $Chi^2 = 1.61$, df = 7 (P = 0.98); $I^2 = 0\%$									
**Coatings vs Concrete**									
Solar-reflective Cool Pavement1 {Middel 2020}	43.74	6.0457	9	43.71	5.8736	9	3.9%	0.03	[-5.47; 5.54]
Solar-reflective Cool Pavement2 {Middel 2020}	45.66	6.5787	7	44.56	6.2016	7	3.4%	1.10	[-5.60; 7.80]
**Total (95% CI)**			**16**			**16**	**7.3%**	**0.46**	**[-6.19; 7.11]**
Heterogeneity: $Tau^2 = 0$; $Chi^2 = 0.06$, df = 1 (P = 0.81); $I^2 = 0\%$									
**Metals vs Concrete**									
Conventional aluminum tile {Elqattan 2021}	43.11	1.0425	14	41.89	0.7931	14	5.4%	1.22	[0.54; 1.91]
Cool aluminum tile {Elqattan 2021}	29.63	0.2920	14	41.89	0.7931	14	5.4%	-12.26	[-12.70; -11.81]
Cool aluminum box {Elqattan 2021}	27.91	0.3302	14	41.89	0.7931	14	5.4%	-13.97	[-14.42; -13.52]
Iron {Liang 2019}	30.28	9.0799	9	30.68	9.6687	9	2.7%	-0.40	[-9.07; 8.27]
**Total (95% CI)**			**51**			**51**	**19.0%**	**-6.74**	**[-19.34; 5.85]**
Heterogeneity: $Tau^2 = 60.1892$; $Chi^2 = 1414.96$, df = 3 (P < 0.01); $I^2 = 100\%$									
**Miscellaneous vs Concrete**									
Cystosepiment {Liang 2019}	30.83	14.0178	9	30.68	9.6687	9	2.1%	0.16	[-10.97; 11.28]
**Natural Materials vs Concrete**									
Wood {Liang 2019}	30.33	11.6539	9	30.68	9.6687	9	2.4%	-0.34	[-10.24; 9.55]
Grass {Takebayashi 2012}	29.67	3.9686	9	34.47	6.5715	9	4.1%	-4.80	[-9.82; 0.22]
Soil {Takebayashi 2012}	32.28	8.2957	9	34.47	6.5715	9	3.3%	-2.19	[-9.10; 4.73]
**Total (95% CI)**			**27**			**27**	**9.8%**	**-3.39**	**[-8.55; 1.77]**
Heterogeneity: $Tau^2 = 0$; $Chi^2 = 0.78$, df = 2 (P = 0.68); $I^2 = 0\%$									
**Total (95% CI)**			**342**			**342**	**100.0%**	**-1.14**	**[-2.91; 0.63]**
Heterogeneity: $Tau^2 = 20.0097$; $Chi^2 = 1712.61$, df = 28 (P = 0); $I^2 = 98\%$									
Test for subgroup differences: $Chi^2 = 39.40$, df = 5 (P < 0.01)									

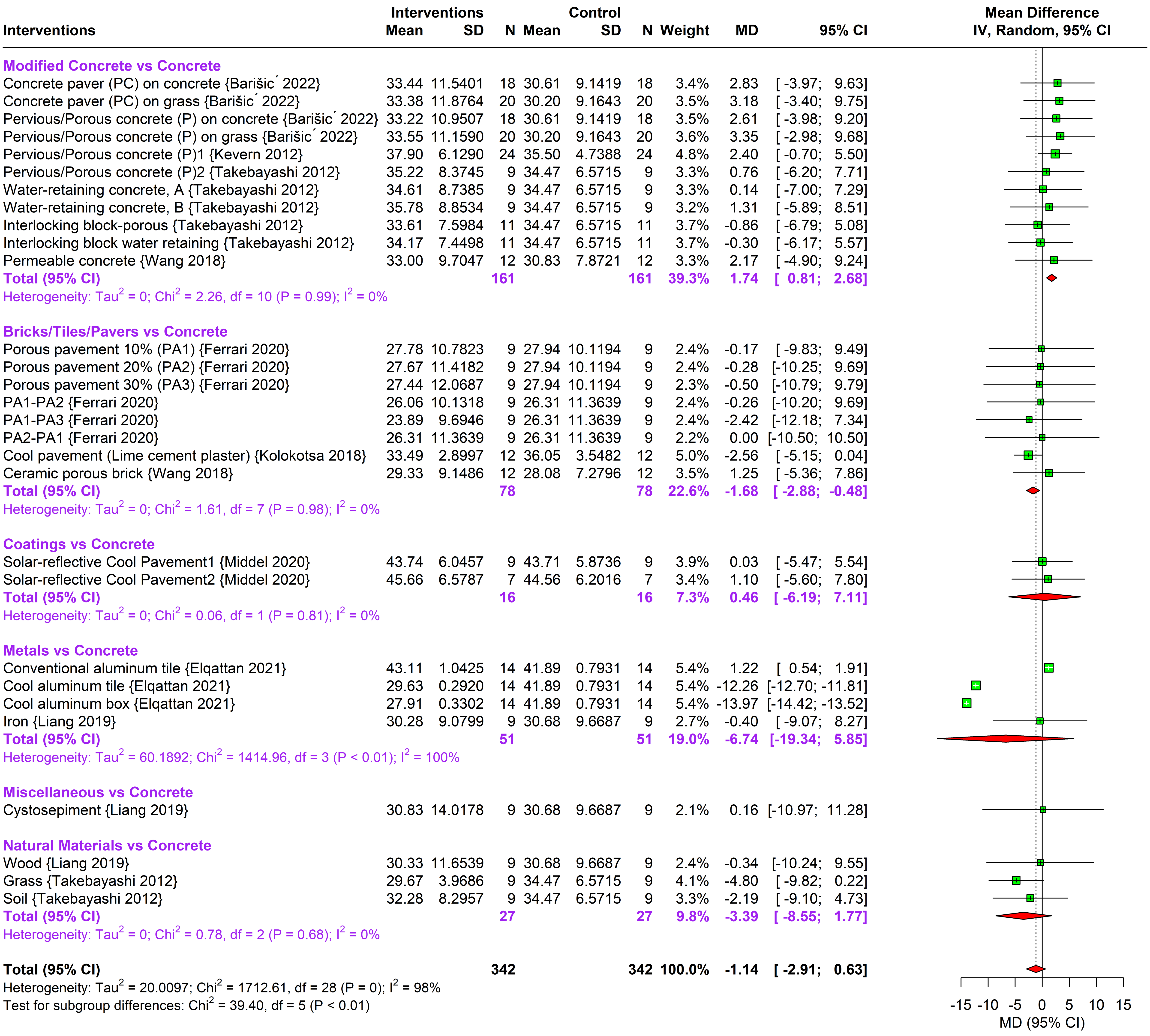

Interventions	Interventions Mean	Interventions SD	N	Control Mean	Control SD	N	Weight	MD	95% CI
**Modified Asphalt vs Asphalt**									
Conventional faint asphalt (S2) {Kyriakodis 2018}	61.41	3.8976	11	64.23	4.4686	11	9.5%	-2.82	[-6.32; 0.69]
Light yellow cool asphalt (S3) {Kyriakodis 2018}	55.82	4.1004	11	64.23	4.4686	11	9.2%	-8.41	[-11.99; -4.83]
Light yellow cool contaminated asphalt (S4) {Kyriakodis 2018}	58.82	4.2206	11	64.23	4.4686	11	9.0%	-5.41	[-9.04; -1.78]
Porous asphalt {Takebayashi 2012}	36.61	8.9435	9	34.47	6.5715	9	2.7%	2.14	[-5.11; 9.40]
Water-retaining asphalt, A {Takebayashi 2012}	34.67	7.5664	9	34.47	6.5715	9	3.3%	0.20	[-6.35; 6.75]
Water-retaining asphalt, B {Takebayashi 2012}	33.61	7.1273	9	34.47	6.5715	9	3.5%	-0.86	[-7.19; 5.48]
**Total (95% CI)**			**60**			**60**	**37.1%**	**-3.35**	**[-7.34; 0.65]**
Heterogeneity: $Tau^2 = 8.2960$; $Chi^2 = 12.07$, df = 5 (P = 0.03); $I^2 = 59\%$									
**Coatings vs Asphalt**									
Coated Asphalt {Guntor 2014}	43.67	6.9652	8	43.95	5.6949	8	3.6%	-0.28	[-6.51; 5.96]
Green {Carnielo 2013}	30.67	15.1668	21	38.10	16.4679	21	1.6%	-7.43	[-17.00; 2.15]
Blue {Carnielo 2013}	35.57	14.9183	21	39.38	17.5883	21	1.5%	-3.81	[-13.67; 6.05]
Red {Carnielo 2013}	35.62	14.9013	21	38.10	16.4679	21	1.6%	-2.48	[-11.97; 7.02]
Off-white {Carnielo 2013}	26.48	9.4848	21	39.38	17.5883	21	2.0%	-12.90	[-21.45; -4.36]
Grey {Carnielo 2013}	29.14	13.7379	21	39.38	17.5883	21	1.6%	-10.24	[-19.78; -0.69]
Cool Photocatalytic pavement (colored white) Spl {Kyriakodis 2018}	49.94	3.5920	11	55.44	3.0254	11	13.3%	-5.50	[-8.28; -2.72]
Solar-reflective Cool Pavement1 {Middel 2020}	43.74	6.0457	9	48.58	7.4481	9	3.5%	-4.83	[-11.10; 1.43]
Solar-reflective Cool Pavement2 {Middel 2020}	45.66	6.5787	7	50.23	7.8245	7	2.5%	-4.57	[-12.14; 3.00]
**Total (95% CI)**			**140**			**140**	**31.2%**	**-5.33**	**[-7.55; -3.11]**
Heterogeneity: $Tau^2 = 0$; $Chi^2 = 7.26$, df = 8 (P = 0.51); $I^2 = 0\%$									
**Pavers vs Asphalt**									
Water-retaining pavement 13% {Shimazaki 2022}	37.50	0.5000	3	49.50	6.7639	3	2.4%	-12.00	[-19.67; -4.33]
Water-retaining pavement 25% {Shimazaki 2022}	38.83	1.4434	3	49.50	6.7639	3	2.3%	-10.67	[-18.49; -2.84]
**Total (95% CI)**			**6**			**6**	**4.8%**	**-11.35**	**[-19.82; -2.88]**
Heterogeneity: $Tau^2 = 0$; $Chi^2 = 0.06$, df = 1 (P = 0.81); $I^2 = 0\%$									
**Gravel vs Asphalt**									
D1=PouzzolanC,PouzzolanM,RubberC,Resin,Gravel {Seifeddine 2022}	43.76	11.5768	25	49.79	11.3769	25	3.4%	-6.03	[-12.39; 0.34]
D2=PouzzolanC,PouzzolanM,RubberM,Resin,Gravel {Seifeddine 2022}	42.71	11.8879	28	49.79	11.3769	28	3.7%	-7.07	[-13.17; -0.98]
D3=PouzzolanC,PouzzolanM,PouzzolanF,RubberM,Resin,Gravel {Seifeddine 2022}	42.32	12.2567	28	49.79	11.3769	28	3.6%	-7.46	[-13.66; -1.27]
**Total (95% CI)**			**81**			**81**	**10.8%**	**-6.87**	**[-8.69; -5.05]**
Heterogeneity: $Tau^2 = 0$; $Chi^2 = 0.11$, df = 2 (P = 0.95); $I^2 = 0\%$									
**Natural Materials vs Asphalt**									
Grass {Takebayashi 2012}	29.67	3.9686	9	37.83	9.3775	9	3.2%	-8.17	[-14.82; -1.51]
Soil {Takebayashi 2012}	32.28	8.2957	9	37.83	9.3775	9	2.2%	-5.56	[-13.74; 2.62]
**Total (95% CI)**			**18**			**18**	**5.3%**	**-7.13**	**[-23.37; 9.11]**
Heterogeneity: $Tau^2 = 0$; $Chi^2 = 0.24$, df = 1 (P = 0.63); $I^2 = 0\%$									
**Mixture Cement/Asphalt vs Asphalt**									
UCZ=URHC+CWP+NZ {Higashiyama 2016}	32.00	4.6188	13	39.92	11.1987	13	3.2%	-7.92	[-14.51; -1.34]
UCF=URHC+CWP+FA {Higashiyama 2016}	35.69	7.1108	13	39.92	11.1987	13	2.7%	-4.23	[-11.44; 2.98]
NCZ=NPC+CWP+NZ {Higashiyama 2016}	34.77	7.0964	13	41.69	11.6718	13	2.6%	-6.92	[-14.35; 0.50]
NCF=NPC+CWP+FA {Higashiyama 2016}	37.85	8.5230	13	41.69	11.6718	13	2.3%	-3.85	[-11.70; 4.01]
**Total (95% CI)**			**52**			**52**	**10.9%**	**-5.90**	**[-9.12; -2.68]**
Heterogeneity: $Tau^2 = 0$; $Chi^2 = 0.9$, df = 3 (P = 0.82); $I^2 = 0\%$									
**Total (95% CI)**			**357**			**357**	**100.0%**	**-5.45**	**[-6.75; -4.15]**
Heterogeneity: $Tau^2 = 0.9619$; $Chi^2 = 27.97$, df = 25 (P = 0.31); $I^2 = 11\%$									
Test for subgroup differences: $Chi^2 = 50.29$, df = 5 (P < 0.01)									

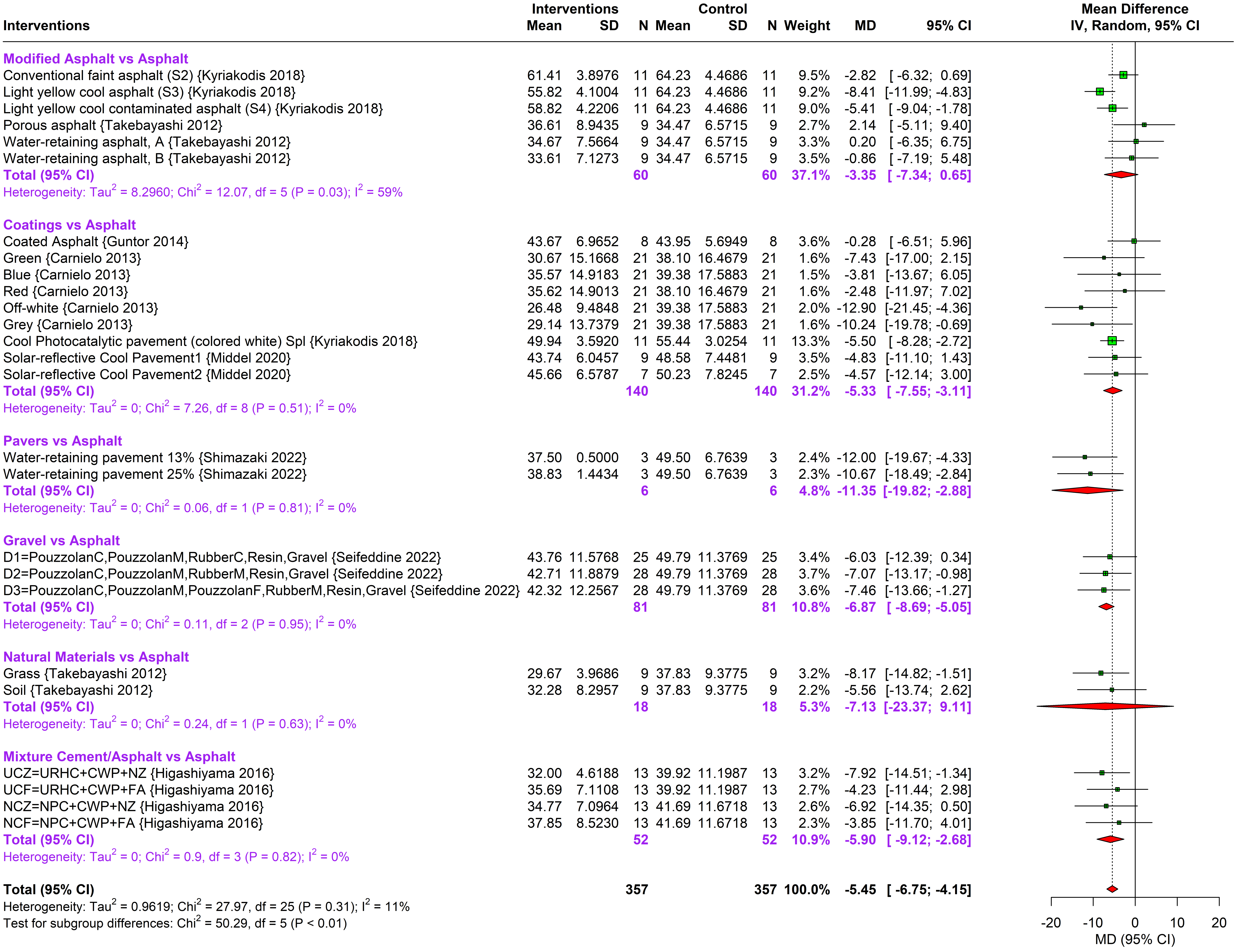

Interventions	Interventions Mean	SD	N	Control Mean	SD	N	Weight	MD	95% CI
**White Gravel vs White Gravel 8-22.4mm**									
White Gravel 0-4 mm {Pisello 2014}	23.50	5.6398	14	30.64	8.0442	14	35.4%	-7.14	[-12.29; -2.00]
White Gravel 2-5.6mm {Pisello 2014}	27.21	6.0151	14	30.64	8.0442	14	33.9%	-3.43	[-8.69; 1.83]
White Gravel 4-12.5mm {Pisello 2014}	28.14	6.8372	14	30.64	8.0442	14	30.7%	-2.50	[-8.03; 3.03]
**Total (95% CI)**			**42**			**42**	**100.0%**	**-4.46**	**[-10.61; 1.69]**

Heterogeneity: $Tau^2 = 0$; $Chi^2 = 1.67$, df = 2 (P = 0.43); $I^2 = 0\%$

Mean Difference
IV, Random, 95% CI

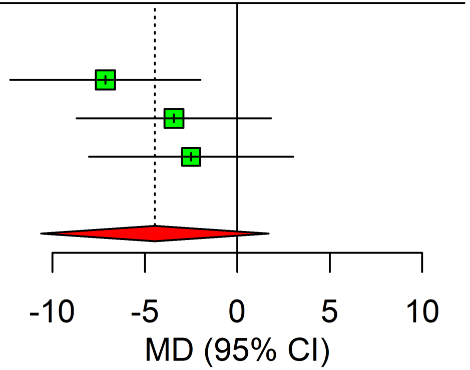

Interventions	Interventions Mean	SD	N	Control Mean	SD	N	Weight	MD	95% CI
**Lighter Bricks vs Black Bricks**									
Black Bricks 80% {Hall 2005}	14.62	4.6980	8	16.25	4.3281	8	27.7%	-1.62	[-6.05; 2.80]
Black Bricks 60% {Hall 2005}	12.19	4.8306	8	16.25	4.3281	8	26.9%	-4.06	[-8.56; 0.43]
Black Bricks 20% {Hall 2005}	10.50	5.5517	8	16.25	4.3281	8	23.0%	-5.75	[-10.63; -0.87]
White Bricks {Hall 2005}	8.97	5.6717	8	16.25	4.3281	8	22.4%	-7.28	[-12.23; -2.34]
**Total (95% CI)**			**32**			**32**	**100.0%**	**-4.50**	**[-8.37; -0.63]**

Heterogeneity: $Tau^2 = 0.3047$; $Chi^2 = 3.12$, df = 3 (P = 0.37); $I^2 = 4\%$

Mean Difference
IV, Random, 95% CI

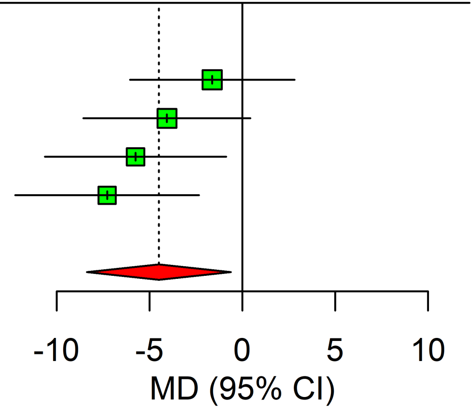

Interventions	Interventions Mean	Interventions SD	N	Control Mean	Control SD	N	Weight	MD	95% CI
**Modified Concrete vs Grass**									
Porous concrete {Takebayashi 2012}	35.22	8.3745	9	29.67	3.9686	9	11.0%	5.56	[-0.50; 11.61]
Water-retaining concrete, A {Takebayashi 2012}	34.61	8.7385	9	29.67	3.9686	9	10.2%	4.94	[-1.33; 11.21]
Water-retaining concrete, B {Takebayashi 2012}	35.78	8.8534	9	29.67	3.9686	9	10.0%	6.11	[-0.23; 12.45]
Interlocking block-porous {Takebayashi 2012}	33.61	7.5984	11	29.67	3.9686	11	15.7%	3.94	[-1.12; 9.01]
Interlocking block water retaining {Takebayashi 2012}	34.17	7.4498	11	29.67	3.9686	11	16.2%	4.50	[-0.49; 9.49]
**Total (95% CI)**			**49**			**49**	**63.1%**	**4.87**	**[3.82; 5.93]**
Heterogeneity: $Tau^2 = 0$; $Chi^2 = 0.35$, df = 4 (P = 0.99); $I^2 = 0\%$									
**Modified Asphalt vs Grass**									
Porous asphalt {Takebayashi 2012}	36.61	8.9435	9	29.67	3.9686	9	9.8%	6.94	[0.55; 13.34]
Water-retaining asphalt, A {Takebayashi 2012}	34.67	7.5664	9	29.67	3.9686	9	12.9%	5.00	[-0.58; 10.58]
Water-retaining asphalt, B {Takebayashi 2012}	33.61	7.1273	9	29.67	3.9686	9	14.2%	3.94	[-1.39; 9.27]
**Total (95% CI)**			**27**			**27**	**36.9%**	**5.11**	**[1.48; 8.74]**
Heterogeneity: $Tau^2 = 0$; $Chi^2 = 0.5$, df = 2 (P = 0.78); $I^2 = 0\%$									
**Total (95% CI)**			**76**			**76**	**100.0%**	**4.96**	**[4.11; 5.81]**
Heterogeneity: $Tau^2 = 0$; $Chi^2 = 0.86$, df = 7 (P = 1.00); $I^2 = 0\%$									
Test for subgroup differences: $Chi^2 = 0.07$, df = 1 (P = 0.80)									

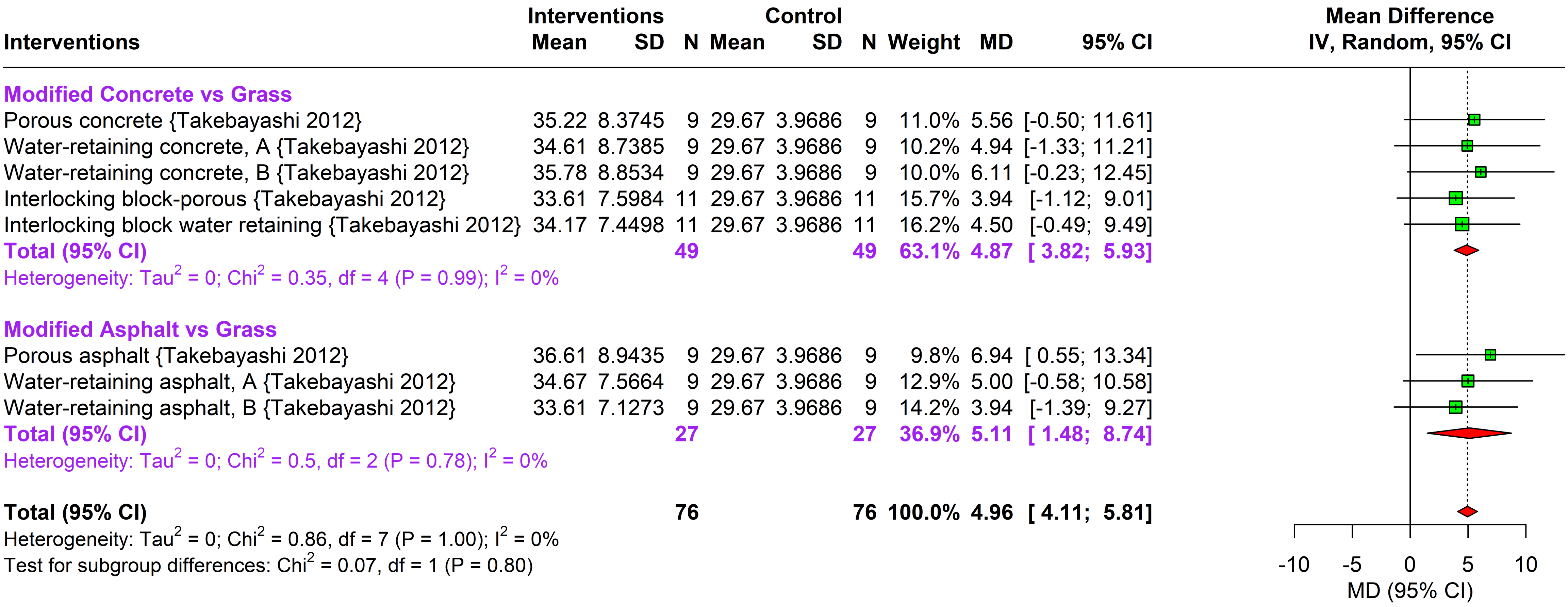

Interventions	Interventions Mean	SD	N	Control Mean	SD	N	Weight	MD	95% CI
**Modified Concrete vs Soil**									
Porous concrete {Takebayashi 2012}	35.22	8.3745	9	32.28	8.2957	9	11.4%	2.94	[-4.76; 10.65]
Water-retaining concrete, A {Takebayashi 2012}	34.61	8.7385	9	32.28	8.2957	9	10.9%	2.33	[-5.54; 10.21]
Water-retaining concrete, B {Takebayashi 2012}	35.78	8.8534	9	32.28	8.2957	9	10.7%	3.50	[-4.43; 11.43]
Interlocking block-porous {Takebayashi 2012}	33.61	7.5984	11	32.28	8.2957	11	15.2%	1.33	[-5.31; 7.98]
Interlocking block water retaining {Takebayashi 2012}	34.17	7.4498	11	32.28	8.2957	11	15.5%	1.89	[-4.70; 8.48]
**Total (95% CI)**			**49**			**49**	**63.7%**	**2.29**	**[1.23; 3.35]**
Heterogeneity: $Tau^2 = 0$; $Chi^2 = 0.21$, df = 4 (P = 0.99); $I^2 = 0\%$									
**Modified Asphalt vs Soil**									
Porous asphalt {Takebayashi 2012}	36.61	8.9435	9	32.28	8.2957	9	10.6%	4.33	[-3.64; 12.30]
Water-retaining asphalt, A {Takebayashi 2012}	34.67	7.5664	9	32.28	8.2957	9	12.5%	2.39	[-4.95; 9.72]
Water-retaining asphalt, B {Takebayashi 2012}	33.61	7.1273	9	32.28	8.2957	9	13.2%	1.33	[-5.81; 8.48]
**Total (95% CI)**			**27**			**27**	**36.3%**	**2.57**	**[-1.12; 6.27]**
Heterogeneity: $Tau^2 = 0$; $Chi^2 = 0.31$, df = 2 (P = 0.86); $I^2 = 0\%$									
**Total (95% CI)**			**76**			**76**	**100.0%**	**2.39**	**[1.53; 3.25]**
Heterogeneity: $Tau^2 = 0$; $Chi^2 = 0.53$, df = 7 (P = 1.00); $I^2 = 0\%$									
Test for subgroup differences: $Chi^2 = 0.09$, df = 1 (P = 0.76)									

Mean Difference
IV, Random, 95% CI

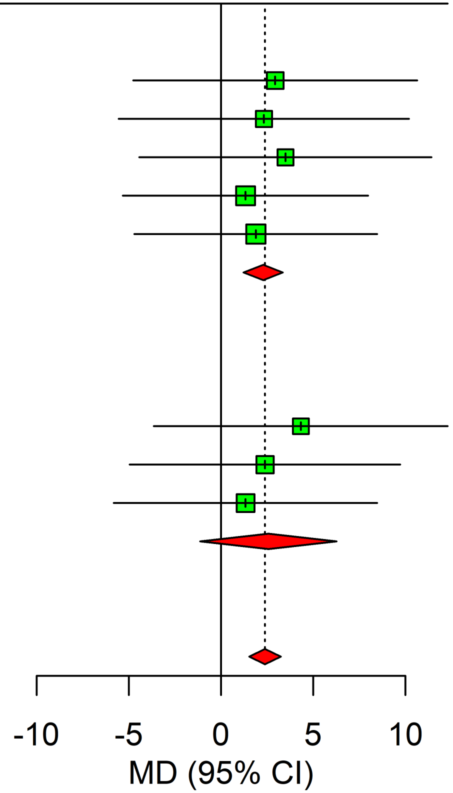

Interventions	Interventions Mean	Interventions SD	Interventions N	Control Mean	Control SD	Control N	Weight	MD	95% CI
**Shade vs Bricked Wall**									
Shade Sail (vertical) {Bakhshoodeh 2022}	26.90	3.1316	10	27.63	4.4056	10	68.3%	-0.73	[-4.08; 2.63]
**Shade vs Wooden Roof**									
Shaded Roof {Zhang 2021}	24.88	5.7650	17	25.35	8.5801	17	31.7%	-0.47	[-5.38; 4.44]
**Total (95% CI)**			**27**			**27**	**100.0%**	**-0.64**	**[-2.15; 0.86]**

Heterogeneity: $Tau^2 = 0$; $Chi^2 = 0.01$, df = 1 (P = 0.93); $I^2 = 0\%$

Test for subgroup differences: $Chi^2 = 0.01$, df = 1 (P = 0.93)

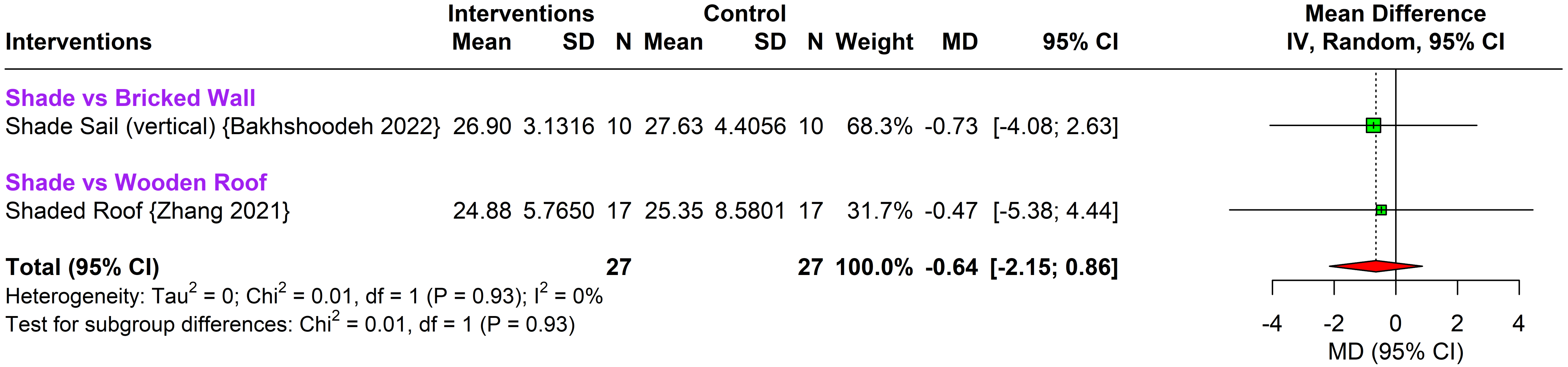

Interventions	Interventions Mean	Interventions SD	N	Control Mean	Control SD	N	Weight	MD	95% CI
**Watering vs Non-watered**									
Irrigated grass {Qian 2020}	13.33	3.0551	3	23.33	3.0551	3	32.4%	-10.00	[-14.89; -5.11]
Watering Pavement {Hendel}	30.85	5.7097	23	35.41	8.6170	23	33.2%	-4.56	[-8.79; -0.34]
**Irrigation vs Common Irrigation Timer**									
MediWater Safe irrigation system {Tomasella 2022}	44.25	4.5000	9	38.50	1.6667	9	34.3%	5.75	[2.61; 8.89]
**Total (95% CI)**			**35**			**35**	**100.0%**	**-2.79**	**[-22.72; 17.15]**

Heterogeneity: $Tau^2 = 60.4324$; $Chi^2 = 33.32$, df = 2 ($P < 0.01$); $I^2 = 94\%$

Test for subgroup differences: $Chi^2 = 16.72$, df = 1 ($P < 0.01$)

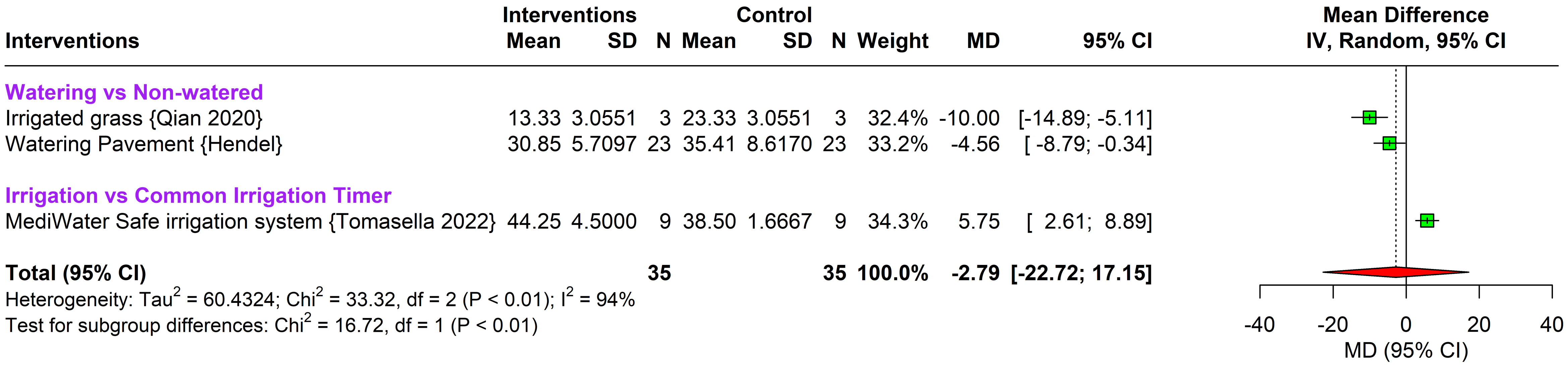

Interventions	Interventions Mean	Interventions SD	N	Control Mean	Control SD	N	Weight	MD	95% CI
**Reflective Roof vs Conventional Roof**									
Reflective Cooling {Lei 2024}	36.97	7.8691	17	39.03	10.8260	17	10.3%	-2.07	[-8.43; 4.30]
Exterior Weather Shield Ultra Clean Coating {Rawat 2022}	39.52	9.1347	24	43.26	10.3142	24	13.7%	-3.74	[-9.25; 1.77]
Exterior TR E2000 {Rawat 2022}	38.59	10.1137	24	43.26	10.3142	24	12.4%	-4.67	[-10.45; 1.11]
Exterior Weather Shield E1000 {Rawat 2022}	39.15	10.0651	24	43.26	10.3142	24	12.5%	-4.11	[-9.87; 1.66]
Exterior Weather Shield Flex {Rawat 2022}	39.61	9.0941	24	43.26	10.3142	24	13.7%	-3.65	[-9.15; 1.85]
Reflective White Paint {Akbari 2003}	25.85	8.1321	28	33.67	18.3240	28	7.5%	-7.81	[-15.24; -0.39]
Both-sided reflective coating {Joudi 2011}	43.38	9.6676	5	50.02	12.9090	5	2.1%	-6.65	[-20.79; 7.49]
Interior reflective coating {Joudi 2011}	50.40	13.2174	5	50.02	12.9090	5	1.6%	0.38	[-15.82; 16.57]
**Total (95% CI)**			**151**			**151**	**73.8%**	**-4.12**	**[-5.59; -2.65]**
Heterogeneity: $Tau^2 = 0$; $Chi^2 = 1.85$, df = 7 (P = 0.97); $I^2 = 0\%$									
**Misc vs Conventional Roof**									
Solar Energy Roof (PVRF) {Tseng 2022}	34.22	7.6449	9	38.33	10.4403	9	5.8%	-4.11	[-12.57; 4.34]
Radiative cooling {Lei 2024}	34.80	4.3486	17	39.03	10.8260	17	13.5%	-4.23	[-9.78; 1.31]
Aerated concrete {Lei 2024}	41.83	12.1821	17	39.03	10.8260	17	6.9%	2.80	[-4.95; 10.55]
**Total (95% CI)**			**43**			**43**	**26.2%**	**-2.26**	**[-11.78; 7.26]**
Heterogeneity: $Tau^2 = 2.0028$; $Chi^2 = 2.31$, df = 2 (P = 0.32); $I^2 = 13\%$									
**Total (95% CI)**			**194**			**194**	**100.0%**	**-3.66**	**[-5.25; -2.06]**
Heterogeneity: $Tau^2 = 0$; $Chi^2 = 4.72$, df = 10 (P = 0.91); $I^2 = 0\%$									
Test for subgroup differences: $Chi^2 = 0.66$, df = 1 (P = 0.42)									

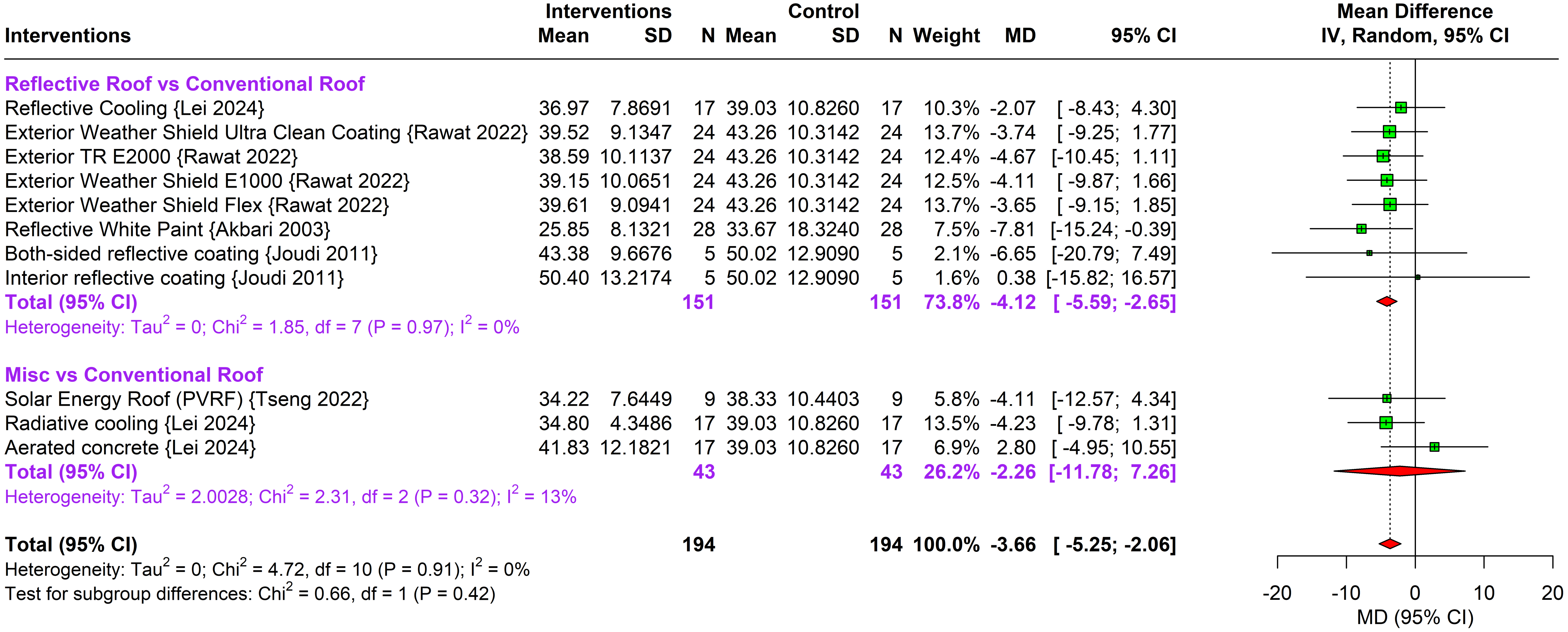

Interventions	Interventions Mean	Interventions SD	N	Control Mean	Control SD	N	Weight	MD	95% CI
**Supercool Roof vs Corrugated metal, white cool roof/commercial cool roof**									
Super-cool polymer multilayer on silver {Gentle 2015}	19.71	3.1472	7	23.00	7.7889	7	11.8%	-3.29	[-9.51; 2.94]
**Solar-reflective Roof vs Galvanized Steel Panel**									
CHMA silicon acrylic cool roof waterproofing material with VOCs {Park 2022}	11.85	14.2800	8766	17.43	18.8080	8766	44.1%	-5.59	[-6.08; -5.09]
**Solar-reflective Roof vs Urethane-based waterproofing material**									
CHMA silicon acrylic cool roof waterproofing material with VOCs {Park 2022}	13.22	16.0040	8766	15.67	19.0940	8766	44.0%	-2.45	[-2.97; -1.93]
**Total (95% CI)**			**17539**			**17539**	**100.0%**	**-3.94**	**[-8.47; 0.60]**

Heterogeneity: $Tau^2 = 3.5993$; $Chi^2 = 73.27$, df = 2 ($P < 0.01$); $I^2 = 97\%$
Test for subgroup differences: $Chi^2 = 73.27$, df = 2 ($P < 0.01$)

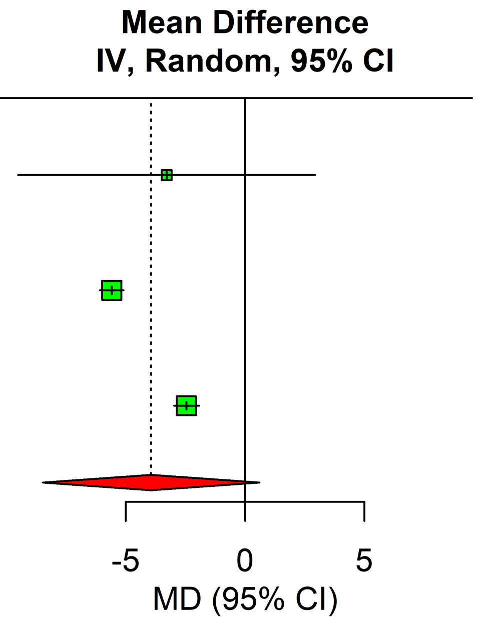

Interventions	Interventions Mean	SD	N	Control Mean	SD	N	Weight	MD	95% CI
**Ventilation vs PCM**									
PCM+Earth-to-air heat exchanger {Guo 2022}	38.00	17.3494	7	35.00	10.5357	7	2.0%	3.00	[-12.04; 18.04]
PCM Ventilated Window {Hu 2020}	17.59	4.8107	39	17.41	4.9028	39	98.0%	0.18	[-1.98; 2.34]
**Total (95% CI)**			**46**			**46**	**100.0%**	**0.24**	**[-4.80; 5.27]**

Heterogeneity: $Tau^2 = 0$; $Chi^2 = 0.13$, df = 1 (P = 0.72); $I^2 = 0\%$

Test for subgroup differences: $Chi^2 = 0.00$, df = 0 (P = NA)

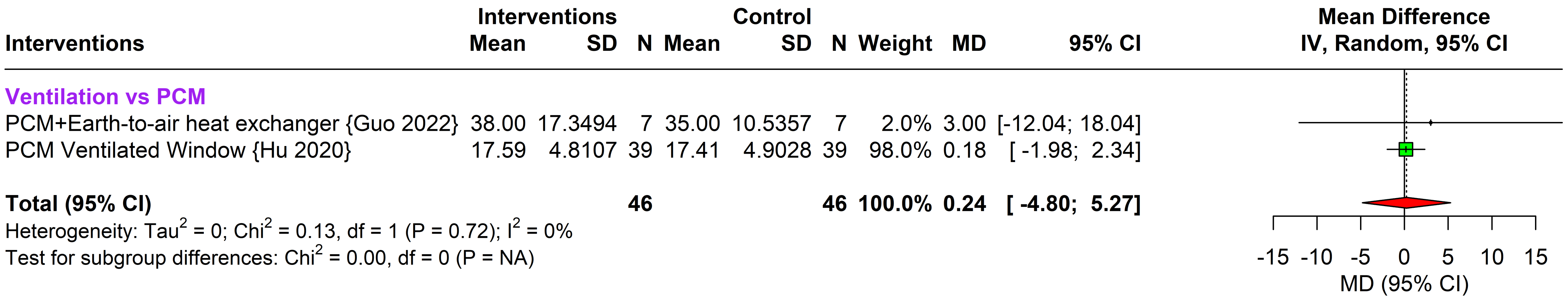

Supplement: online supplemental file 4 [file bmjph-3-2-s004.pdf]
