# Supplementary material for: Evaluation of community-based heat adaptation interventions: a systematic review
Source: BMJ Public Health. 2025 Jul 15;3(2):e002332. doi: 10.1136/bmjph-2024-002332 (PMC12273142; doi:10.1136/bmjph-2024-002332)

ANNEX 4 - Meta-analysis for Indoor Temperature

Interventions	Interventions Mean	Interventions SD	N	Control Mean	Control SD	N	Weight	MD	95% CI
**Green Roof vs Conventional Roof**									
Green Roof {Cirrincione 2020}	21.43	4.5015	14	21.50	5.7211	14	4.9%	-0.07	[-3.88; 3.74]
Green Roof {Jaffal 2012}	27.04	1.8003	14	29.55	1.4538	14	9.5%	-2.51	[-3.73; -1.30]
Green Roof {Kumar 2016}	22.50	3.7081	5	25.60	2.4341	5	4.8%	-3.10	[-6.99; 0.79]
Green Roof {Muharam 2016}	35.29	3.1565	26	37.65	4.3630	26	8.0%	-2.37	[-4.44; -0.30]
Green Roof {Ouldboukhitine 2013}	22.20	3.9316	15	22.53	4.3894	15	6.2%	-0.33	[-3.32; 2.65]
Green Roof {Pandey 2012}	26.11	4.4429	24	30.10	4.6164	24	7.0%	-3.99	[-6.55; -1.43]
Green Roof {Saify 2023}	38.81	3.4115	8	39.38	3.2705	8	5.8%	-0.56	[-3.84; 2.71]
Green Roof {Tseng 2022}	32.50	2.0000	10	31.95	2.5868	10	8.0%	0.55	[-1.48; 2.58]
Green Roof - Rio de Janeiro {Wilkinson 2015}	33.00	3.5824	13	33.77	4.2652	13	6.2%	-0.77	[-3.80; 2.26]
Green Roof - Sydney {Wilkinson 2015}	25.11	3.6209	9	27.11	7.7208	9	3.1%	-2.00	[-7.57; 3.57]
Extensive Green Roof {Tang 2019}	29.90	1.1402	5	33.00	1.4142	5	8.9%	-3.10	[-4.69; -1.51]
Green Roof - Stone Crop {Seo 2023}	30.21	0.3934	7	35.79	1.5774	7	9.6%	-5.57	[-6.78; -4.37]
Green Roof - Grass {Seo 2023}	30.21	0.3934	7	35.79	1.5774	7	9.6%	-5.57	[-6.78; -4.37]
Multilayer Green Roof {Pumo 2023}	20.10	9.9900	5	21.40	11.1770	5	0.7%	-1.30	[-14.44; 11.84]
Green Energy Roof {Tseng 2022}	31.05	2.3740	10	31.95	2.5868	10	7.7%	-0.90	[-3.08; 1.28]
**Total (95% CI)**			**172**			**172**	**100.0%**	**-2.40**	**[-3.54; -1.26]**

Heterogeneity: $Tau^2 = 3.2723$; $Chi^2 = 60.80$, df = 14 ($P < 0.01$); $I^2 = 77\%$

Mean Difference
IV, Random, 95% CI

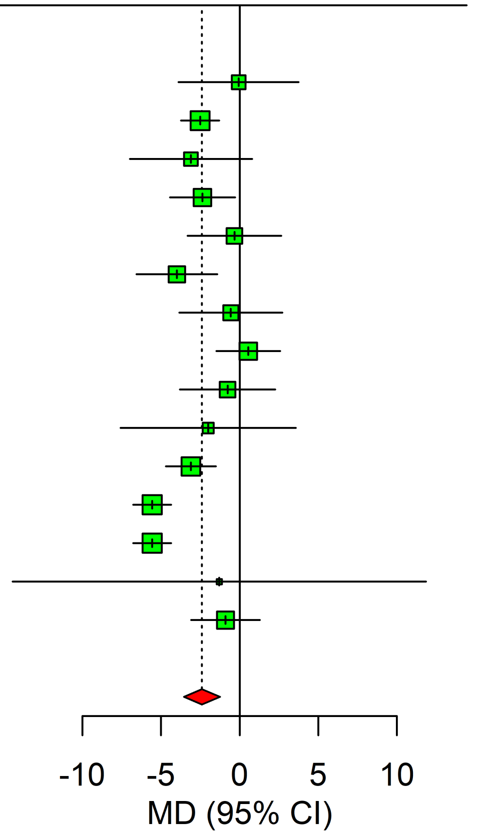

Interventions	Interventions Mean	SD	N	Control Mean	SD	N	Weight	MD	95% CI
**Green Roof vs Conventional Steel Sheet**									
Extensive Green Roof {Coutts 2013}	22.91	3.0497	22	23.68	6.7833	22	50.8%	-0.77	[-3.88; 2.34]
**Soil vs Conventional Steel Sheet**									
Soil substrate {Coutts 2013}	23.07	3.3213	22	23.68	6.7833	22	49.2%	-0.61	[-3.77; 2.54]
**Total (95% CI)**			**44**			**44**	**100.0%**	**-0.69**	**[-1.71; 0.32]**

Heterogeneity: $Tau^2 = 0$; $Chi^2 = 0.00$, df = 1 (P = 0.94); $I^2 = 0\%$

Test for subgroup differences: $Chi^2 = 0.00$, df = 1 (P = 0.94)

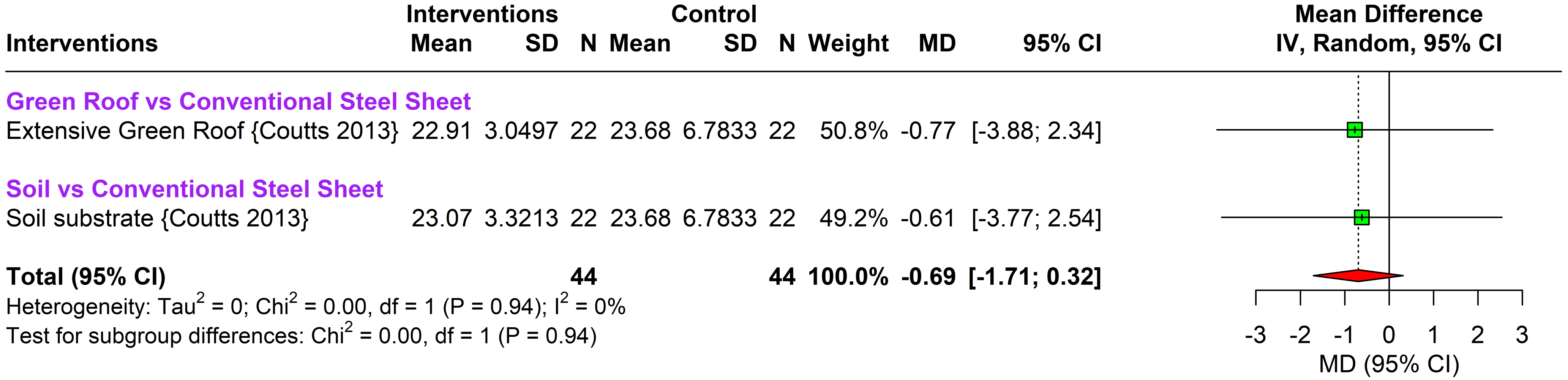

Interventions	Interventions Mean	SD	N	Control Mean	SD	N	Weight	MD	95% CI
**Green Roof vs Wood Roof**									
Sand substrate {Sisco 2017}	32.25	1.9685	14	33.21	2.7012	14	5.1%	-0.96	[-2.72; 0.79]
Pellet substrate {Sisco 2017}	33.14	2.6343	14	33.21	2.7012	14	4.1%	-0.07	[-2.05; 1.91]
Extensive Green Roof - Ground Cover Plants {Yildirim 2023}	31.24	8.5200	1584	31.62	10.1050	1584	29.2%	-0.38	[-1.03; 0.27]
Extensive Green Roof - Mix Vegetation {Yildirim 2023}	30.57	7.8340	1584	31.62	10.1050	1584	30.7%	-1.05	[-1.68; -0.42]
Extensive Green Roof - Shrubs {Yildirim 2023}	30.50	7.7480	1584	31.62	10.1050	1584	30.9%	-1.12	[-1.75; -0.49]
**Total (95% CI)**			**4780**			**4780**	**100.0%**	**-0.83**	**[-1.32; -0.34]**

Heterogeneity: $Tau^2 = 0.0366$; $Chi^2 = 3.72$, df = 4 (P = 0.44); $I^2 = 0\%$

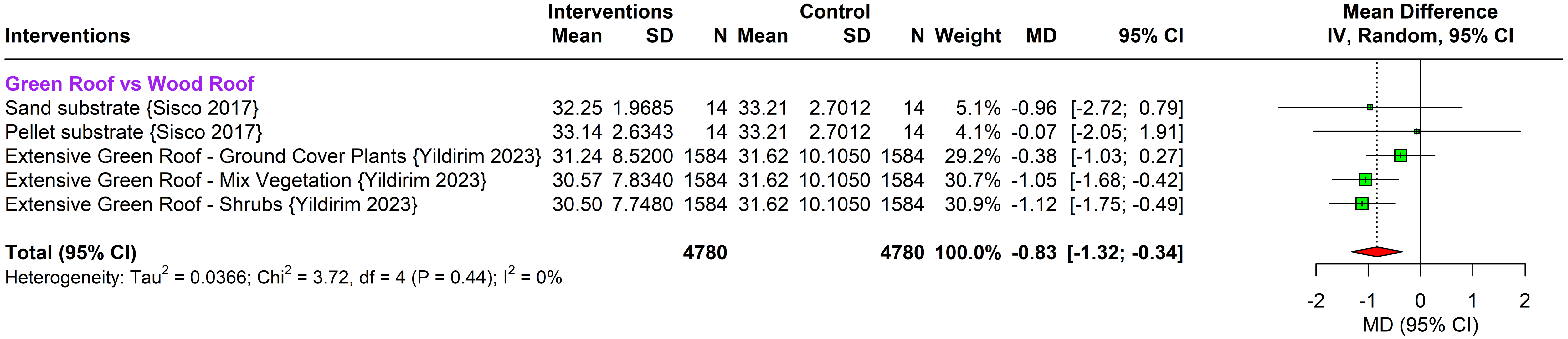

Interventions	Interventions Mean	SD	N	Control Mean	SD	N	Weight	MD	95% CI
**Green Roof vs Black Roof**									
Green Roof {Pompeii II 2010}	22.61	3.8588	24	24.78	6.0724	24	64.4%	-2.17	[-5.05; 0.71]
**Green Roof vs Green Paint**									
Green Roof - Poa Pratensis {Lee 2015}	27.67	1.2111	6	32.42	6.7559	6	17.7%	-4.75	[-10.24; 0.74]
Green Roof - Sedum Sarmentosum {Lee 2015}	27.00	0.9487	6	32.42	6.7559	6	17.9%	-5.42	[-10.88; 0.04]
**Total (95% CI)**			**36**			**36**	**100.0%**	**-3.21**	**[-7.50; 1.08]**

Heterogeneity: $Tau^2 = 0$; $Chi^2 = 1.43$, df = 2 (P = 0.49); $I^2 = 0\%$

Test for subgroup differences: $Chi^2 = 3.75$, df = 1 (P = 0.05)

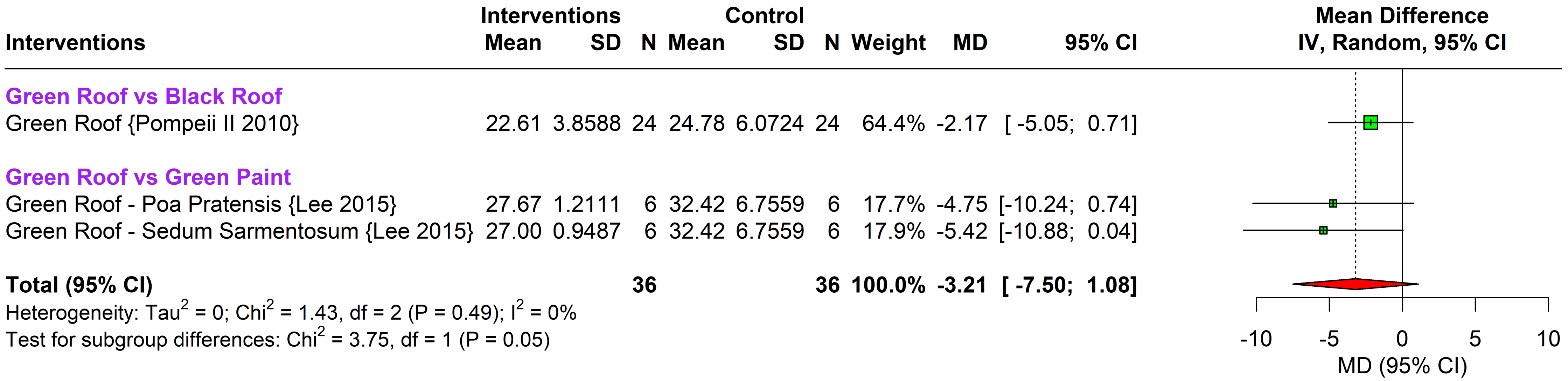

Interventions	Interventions Mean	Interventions SD	N	Control Mean	Control SD	N	Weight	MD	95% CI
**Green Facade vs Bare wall**									
Green Facade {Safikhani 2014}	28.96	4.2064	24	30.46	6.3724	24	3.7%	-1.50	[-4.55; 1.55]
Green Facade {Widiastuti 2018}	28.14	2.3487	14	31.64	3.0536	14	4.8%	-3.50	[-5.52; -1.48]
Green Facade {Haggag 2017}	36.20	2.6780	15	37.60	6.6526	15	3.2%	-1.40	[-5.03; 2.23]
Green Facade {Susorova 2013}	23.41	2.0931	8	24.24	2.2557	8	4.7%	-0.82	[-2.96; 1.31]
Green Facade {Susorova 2014}	24.80	1.1000	12481	25.30	0.7000	12481	6.3%	-0.50	[-0.52; -0.48]
Green Facade {Widiastuti 2018}	24.21	1.8051	14	29.64	2.5603	14	5.2%	-5.43	[-7.07; -3.79]
Green Facade A {Saify 2023}	35.19	2.5062	8	35.97	2.8361	8	4.1%	-0.78	[-3.40; 1.84]
Green Façade B {Saify 2023}	29.50	4.4159	8	31.81	5.0351	8	2.4%	-2.31	[-6.95; 2.33]
Double-skin Green Façade {Perez 2017}	27.76	0.8794	25	30.72	1.2754	25	6.1%	-2.96	[-3.57; -2.35]
Double-skin Green Façade {Coma 2017}	4.85	1.2810	13	4.38	0.7679	13	6.0%	0.46	[-0.35; 1.27]
**Total (95% CI)**			**12610**			**12610**	**46.5%**	**-1.85**	**[-3.18; -0.52]**
Heterogeneity: $Tau^2 = 2.9287$; $Chi^2 = 112.8$, df = 9 ($P < 0.01$); $I^2 = 92\%$									
**Living Wall vs Bare wall**									
Geogreen module {Manso 2016}	18.70	3.1641	76	19.27	7.0571	76	5.1%	-0.57	[-2.31; 1.17]
Living Wall {Chen 2013}	33.59	1.9059	17	36.12	4.5261	17	4.5%	-2.53	[-4.86; -0.19]
Living Wall {He 2017}	33.33	3.2004	12	33.58	3.3967	12	4.1%	-0.25	[-2.89; 2.39]
Living Wall {Safikhani 2014}	28.62	3.8427	24	30.46	6.3724	24	3.8%	-1.83	[-4.81; 1.14]
Living Wall {Haggag 2014}	49.14	4.5251	7	54.14	1.2150	7	3.3%	-5.00	[-8.47; -1.53]
Living Wall {Olivieri 2014}	24.55	2.0245	53	31.22	2.2861	53	6.0%	-6.67	[-7.49; -5.85]
Living Wall {Coma 2017}	2.85	0.6887	13	4.38	0.7679	13	6.1%	-1.54	[-2.10; -0.98]
Living Wall {Djedjig 2015}	26.62	0.8561	12	26.71	1.3561	12	5.9%	-0.08	[-0.99; 0.82]
Living Wall {Shah 2023}	24.12	1.5478	4	28.50	1.2247	4	4.9%	-4.38	[-6.31; -2.44]
Continuous Living Wall {Cruciol-Barbosa 2023}	25.14	1.0336	58	26.81	2.6054	58	6.0%	-1.67	[-2.39; -0.95]
Felt layer {Nori 2013}	15.79	0.4149	24	17.00	0.0000	24	0.0%	-1.21	
Vertical greening systems {Yang 2023}	25.33	3.2787	9	25.78	3.1929	9	3.8%	-0.44	[-3.43; 2.55]
**Total (95% CI)**			**309**			**309**	**53.5%**	**-2.27**	**[-3.76; -0.78]**
Heterogeneity: $Tau^2 = 4.2448$; $Chi^2 = 155.92$, df = 10 ($P < 0.01$); $I^2 = 94\%$									
**Total (95% CI)**			**12919**			**12919**	**100.0%**	**-2.08**	**[-3.00; -1.16]**

Heterogeneity: $Tau^2 = 3.4753$; $Chi^2 = 377.85$, df = 20 ($P < 0.01$); $I^2 = 95\%$

Test for subgroup differences: $Chi^2 = 0.22$, df = 1 ($P = 0.64$)

Mean Difference
IV, Random, 95% CI

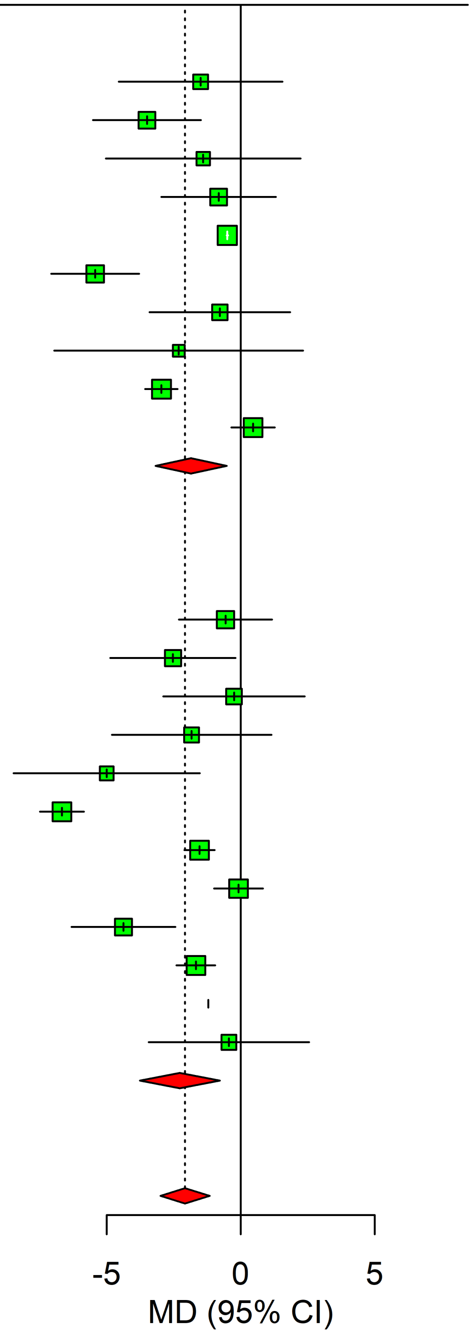

Interventions	Interventions Mean	SD	N	Control Mean	SD	N	Weight	MD	95% CI
**Green Facade vs Bricked wall**									
Green Façade A {Benhalilou 2020}	28.28	0.9358	11	30.62	1.4811	11	52.5%	-2.34	[-3.37; -1.30]
Green Facade C {Benhalilou 2020}	29.43	1.2681	11	30.62	1.4811	11	47.5%	-1.19	[-2.34; -0.04]
**Total (95% CI)**			**22**			**22**	**100.0%**	**-1.79**	**[-9.06; 5.48]**

Heterogeneity: $Tau^2 = 0.3437$; $Chi^2 = 2.10$, df = 1 (P = 0.15); $I^2 = 52\%$

Mean Difference
IV, Random, 95% CI

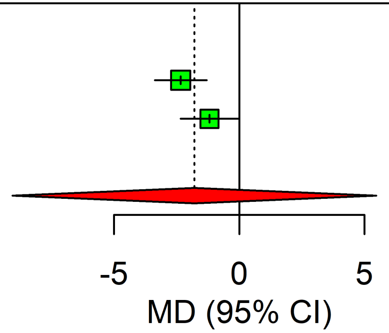

Interventions	Interventions Mean	Interventions SD	Interventions N	Control Mean	Control SD	Control N	Weight	MD	95% CI
**Living Wall vs Double Glazed Façade**									
Double Green Glazed Facade {Bao 2022b}	32.44	3.3050	25	33.44	4.4915	25	0.6%	-1.00	[-3.19; 1.19]
**Living Wall vs Double Glazed Façade, air conditioning**									
Double Green Glazed Facade, air conditioning {Bao 2022b}	23.81	0.1809	25	24.56	0.4012	25	98.2%	-0.75	[-0.93; -0.58]
**Living Wall vs Double Glazed Façade, with louvers**									
Double Green Glazed Facade {Bao 2022b}	31.41	2.5995	17	31.41	3.5542	17	0.7%	0.00	[-2.09; 2.09]
**Living Wall vs Double Glazed Façade, no ventilation**									
Double Green Glazed Facade, with ventilation {Bao 2022b}	28.41	3.5366	17	29.59	3.6922	17	0.5%	-1.18	[-3.61; 1.25]
**Total (95% CI)**			**84**			**84**	**100.0%**	**-0.75**	**[-0.88; -0.62]**

Heterogeneity: $Tau^2 = 0$; $Chi^2 = 0.66$, df = 3 (P = 0.88); $I^2 = 0\%$
Test for subgroup differences: $Chi^2 = 0.66$, df = 3 (P = 0.88)

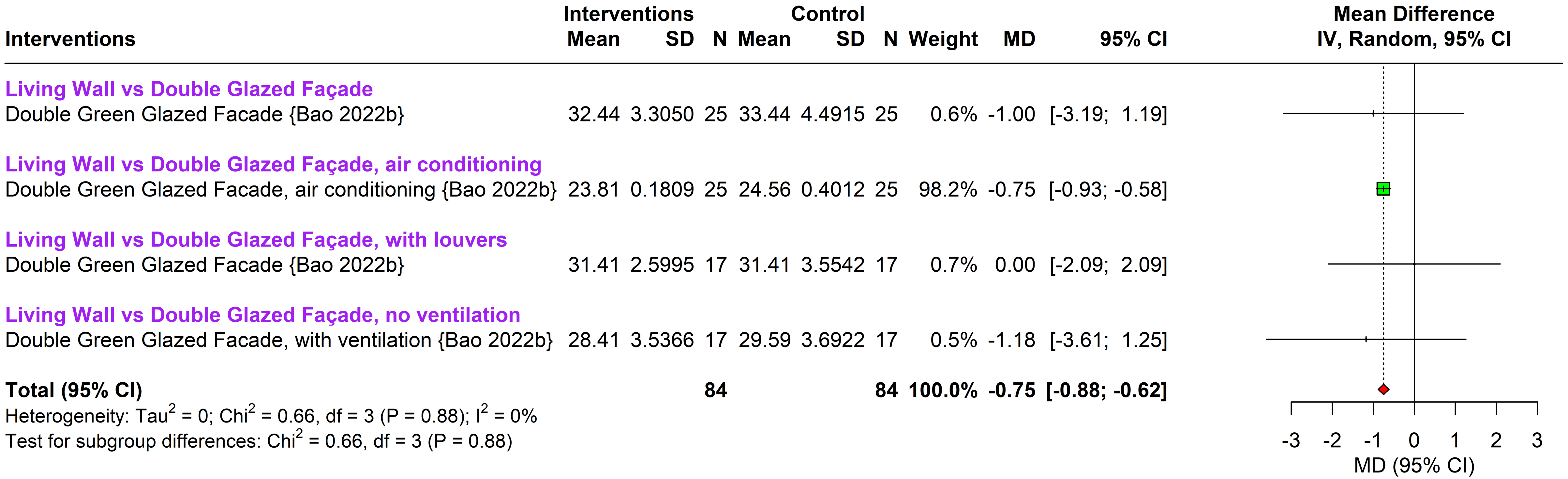

Interventions	Interventions Mean	Interventions SD	Interventions N	Control Mean	Control SD	Control N	Weight	MD	95% CI
**Living Wall vs External VGS**									
Indoor Vertical greening systems {Yang 2023}	23.22	2.9907	9	23.78	3.5978	9	21.7%	-0.56	[-3.61; 2.50]
**Living Wall vs Unshaded Window**									
Modular Vertical Greening Shading Device {Bao 2022a}	32.60	2.9861	25	34.56	4.2237	25	49.3%	-1.96	[-3.99; 0.07]
**Living Wall vs Window with Louver**									
Modular Vertical Greening Shading Device {Bao 2022a}	31.65	3.5345	17	32.24	4.2943	17	29.0%	-0.59	[-3.23; 2.06]
**Total (95% CI)**			**51**			**51**	**100.0%**	**-1.26**	**[-3.37; 0.85]**

Heterogeneity: $Tau^2 = 0$; $Chi^2 = 0.91$, df = 2 (P = 0.63); $I^2 = 0\%$

Test for subgroup differences: $Chi^2 = 0.91$, df = 2 (P = 0.63)

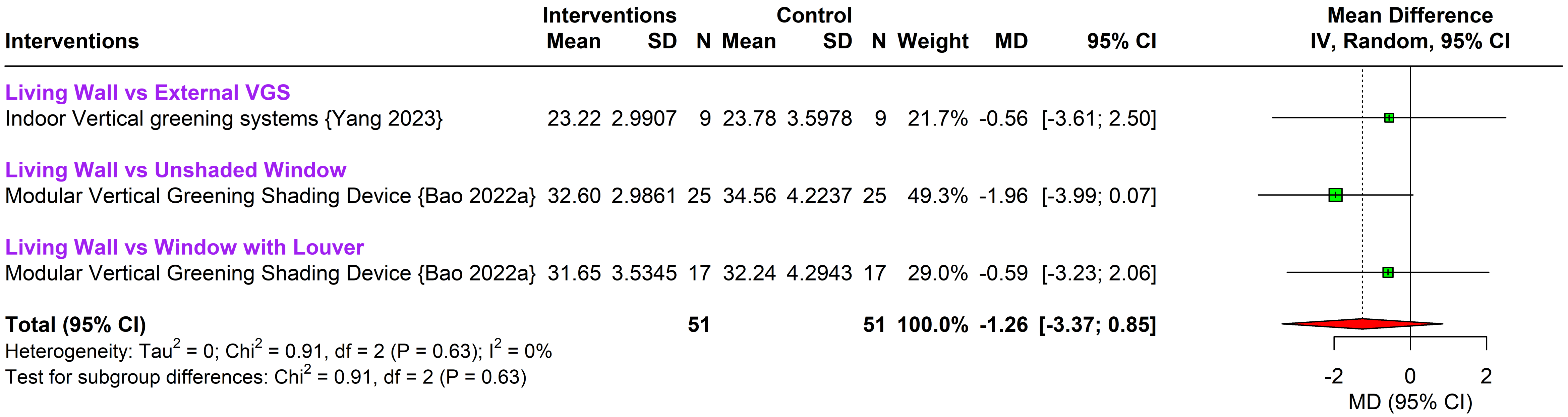

Interventions	Interventions Mean	SD	N	Control Mean	SD	N	Weight	MD	95% CI
**Vegetated House vs Non-vegetated house**									
Vegetated House {Saify 2023}	40.83	2.2776	9	42.00	2.4109	9	32.4%	-1.17	[-3.33; 1.00]
Vegetated House - Brazil {Wilkinson 2016}	27.28	4.0741	57	28.63	5.8206	57	33.4%	-1.35	[-3.20; 0.49]
Vegetated House - Australia {Wilkinson 2016}	25.96	2.9713	54	32.78	4.9704	54	34.3%	-6.81	[-8.36; -5.27]
**Total (95% CI)**			**120**			**120**	**100.0%**	**-3.16**	**[-11.19; 4.86]**

Heterogeneity: $Tau^2 = 9.6642$; $Chi^2 = 27.08$, df = 2 ($P < 0.01$); $I^2 = 93\%$

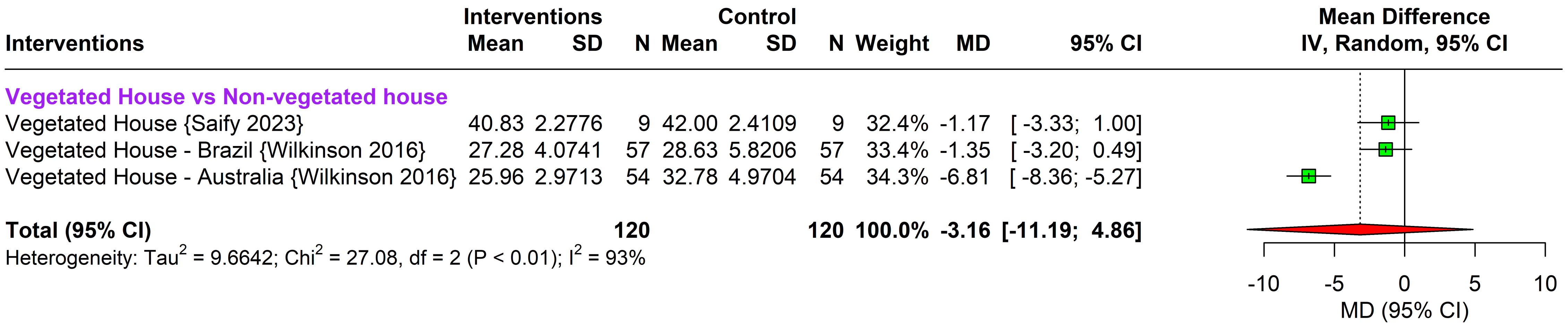

Interventions	Interventions Mean	Interventions SD	Interventions N	Control Mean	Control SD	Control N	Weight	MD	95% CI
**Shade Sail vs Unshaded**									
Shade Sail {Buller 2017}	25.60	4.8000	144	25.40	4.4000	432	50.2%	0.20	[-0.69; 1.09]
HDPE Plastic Sheet {Jareemit 2022}	31.67	3.7739	12	32.38	4.3543	12	3.7%	-0.71	[-3.97; 2.55]
Mylar Rescue Blanket {Karanja 2023}	39.70	5.7600	865	39.50	6.4600	.	0.0%		
White Cotton Bedsheet {Karanja 2023}	40.60	7.0400	865	39.50	6.4600	.	0.0%		
Blue Tarp {Karanja 2023}	41.90	8.8500	865	39.50	6.4600	.	0.0%		
Sunbrella Fabric {Karanja 2023}	41.80	8.2800	865	39.50	6.4600	.	0.0%		
Aluminium Foil {Karanja 2023}	39.50	5.2200	865	39.50	6.4600	.	0.0%		
**Shade Structure vs Unshaded**									
Egg-crate Shading device {Calama-Gonzalez 2019}	0.79	21.1333	90	0.85	0.7756	21	2.1%	-0.06	[-4.43; 4.32]
Shaded {Lee 2020}	32.43	1.5549	14	32.79	1.4769	14	31.3%	-0.36	[-1.48; 0.77]
Shaded Roof {Zhang 2021}	23.35	2.7600	17	23.53	5.1005	17	5.2%	-0.18	[-2.93; 2.58]
**Misc vs Unshaded**									
Galvanized Steel Sheet {Jareemit 2022}	31.58	3.8009	12	32.38	4.3543	12	3.7%	-0.79	[-4.06; 2.48]
Polycarbonate Sheet {Jareemit 2022}	31.75	3.7203	12	32.38	4.3543	12	3.8%	-0.62	[-3.87; 2.62]
**Total (95% CI)**			**4626**			**520**	**100.0%**	**-0.10**	**[-0.43; 0.23]**

Heterogeneity: $Tau^2 = 0$; $Chi^2 = 1.05$, df = 6 ($P = 0.98$); $I^2 = 0\%$

Test for subgroup differences: $Chi^2 = 20.54$, df = 2 ($P < 0.01$)

Mean Difference
IV, Random, 95% CI

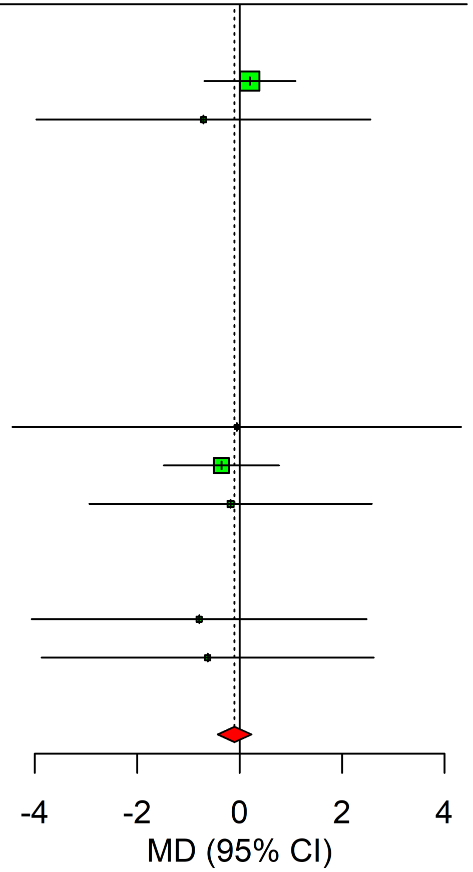

Interventions	Interventions Mean	SD	N	Control Mean	SD	N	Weight	MD	95% CI
**Trees vs Unshaded**									
Pterocarpus macrocarpus tree (LAI: 4.2m2) {Jareemit 2022}	30.83	3.9274	12	32.38	4.3543	12	52.0%	-1.54	[-4.86; 1.78]
**Tress vs Unshaded**									
Pterocarpus macrocarpus tree (LAI: 3.8m2) {Jareemit 2022}	31.83	4.2817	12	32.38	4.3543	12	48.0%	-0.54	[-4.00; 2.91]
**Total (95% CI)**			**24**			**24**	**100.0%**	**-1.06**	**[-7.41; 5.29]**

Heterogeneity: $Tau^2 = 0$; $Chi^2 = 0.17$, df = 1 (P = 0.68); $I^2 = 0\%$
Test for subgroup differences: $Chi^2 = 0.17$, df = 1 (P = 0.68)

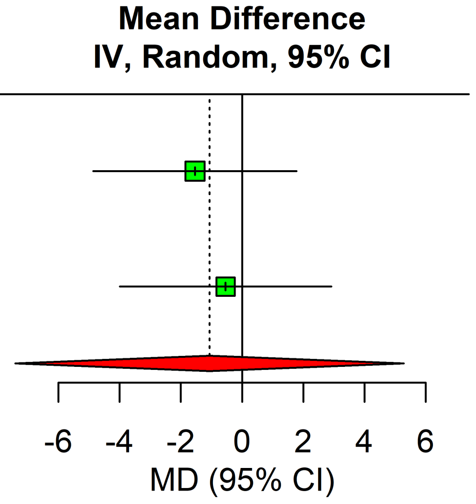

Interventions	Interventions Mean	SD	N	Control Mean	SD	N	Weight	MD	95% CI
**Irrigation vs Non-irrigation**									
Irrigation {Ghafarian 2022}	23.66	3.8190	35	27.66	6.4532	35	48.4%	-4.00	[-6.48; -1.52]
Irrigation {Qian 2020}	23.60	2.2400	8	23.70	1.9600	8	51.6%	-0.10	[-2.16; 1.96]
**Total (95% CI)**			**43**			**43**	**100.0%**	**-1.99**	**[-26.75; 22.78]**

Heterogeneity: $Tau^2 = 6.2480$; $Chi^2 = 5.60$, df = 1 (P = 0.02); $I^2 = 82\%$

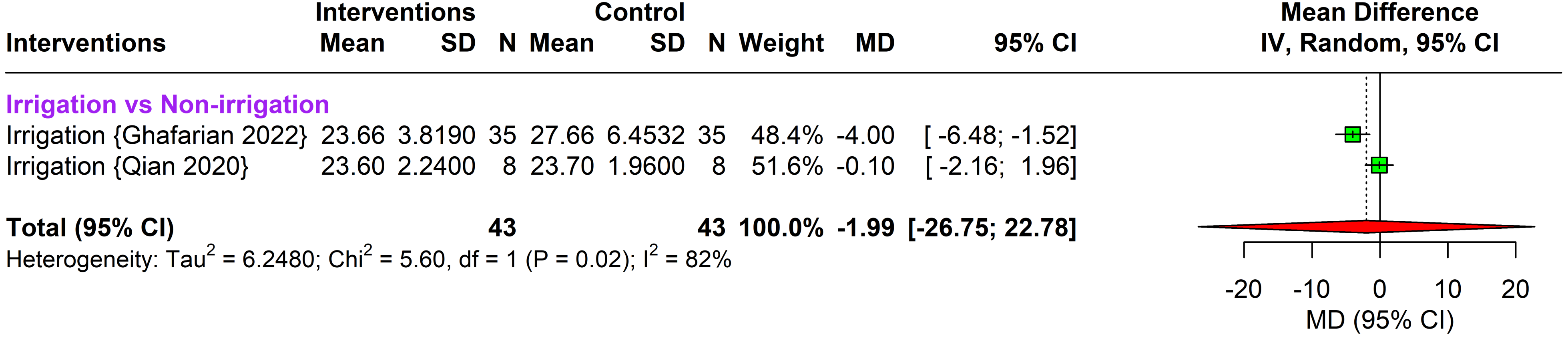

Interventions	Interventions Mean	Interventions SD	N	Control Mean	Control SD	N	Weight	MD	95% CI
**Reflective Roof vs Conventional Roof**									
Solar reflective White Paint {Vellingiri 2020}	33.50	1.1200	15	31.60	0.2100	15	11.6%	1.90	[1.32; 2.48]
High Reflective Paint Roof {Seo 2023}	32.43	1.0965	7	35.79	1.5774	7	9.9%	-3.36	[-4.78; -1.93]
Reflective Cooling {Lei 2024}	39.07	4.3684	17	39.33	4.8398	17	6.0%	-0.27	[-3.37; 2.83]
Exterior Weather Shield Ultra Clean Coating {Rawat 2022}	31.35	3.1277	24	34.00	3.8258	24	8.5%	-2.65	[-4.63; -0.68]
Exterior TR E2000 {Rawat 2022}	31.48	3.6540	24	34.00	3.8258	24	8.2%	-2.52	[-4.64; -0.41]
Exterior Weather Shield E1000 {Rawat 2022}	32.61	3.3404	24	34.00	3.8258	24	8.4%	-1.39	[-3.42; 0.64]
Exterior Weather Shield Flex {Rawat 2022}	31.57	3.5577	24	34.00	3.8258	24	8.2%	-2.43	[-4.52; -0.34]
Reflective White Paint {Akbari 2003}	25.93	6.2075	27	29.42	9.9967	27	3.9%	-3.50	[-7.94; 0.94]
**Total (95% CI)**			**162**			**162**	**64.6%**	**-1.61**	**[-3.22; 0.01]**
Heterogeneity: $Tau^2 = 3.2923$; $Chi^2 = 82.39$, df = 7 ($P < 0.01$); $I^2 = 92\%$									
**Recycled materials vs Conventional Roof**									
Modroof {Vellingiri 2020}	33.80	1.0300	15	33.75	0.7600	15	11.5%	0.05	[-0.60; 0.70]
**Plastic vs Conventional Roof**									
Thermocol Sheet {Vellingiri 2020}	33.50	0.7300	15	33.25	1.0900	15	11.5%	0.25	[-0.41; 0.91]
**Misc vs Conventional Roof**									
Radiative cooling {Lei 2024}	38.80	3.9655	17	39.33	4.8398	17	6.2%	-0.54	[-3.51; 2.44]
Aerated concrete {Lei 2024}	39.47	4.0054	17	39.33	4.8398	17	6.2%	0.13	[-2.85; 3.12]
**Total (95% CI)**			**34**			**34**	**12.4%**	**-0.20**	**[-4.46; 4.05]**
Heterogeneity: $Tau^2 = 0$; $Chi^2 = 0.1$, df = 1 ($P = 0.76$); $I^2 = 0\%$									
**Total (95% CI)**			**226**			**226**	**100.0%**	**-1.00**	**[-2.11; 0.11]**
Heterogeneity: $Tau^2 = 2.4714$; $Chi^2 = 83.30$, df = 11 ($P < 0.01$); $I^2 = 87\%$									
Test for subgroup differences: $Chi^2 = 6.20$, df = 3 ($P = 0.10$)									

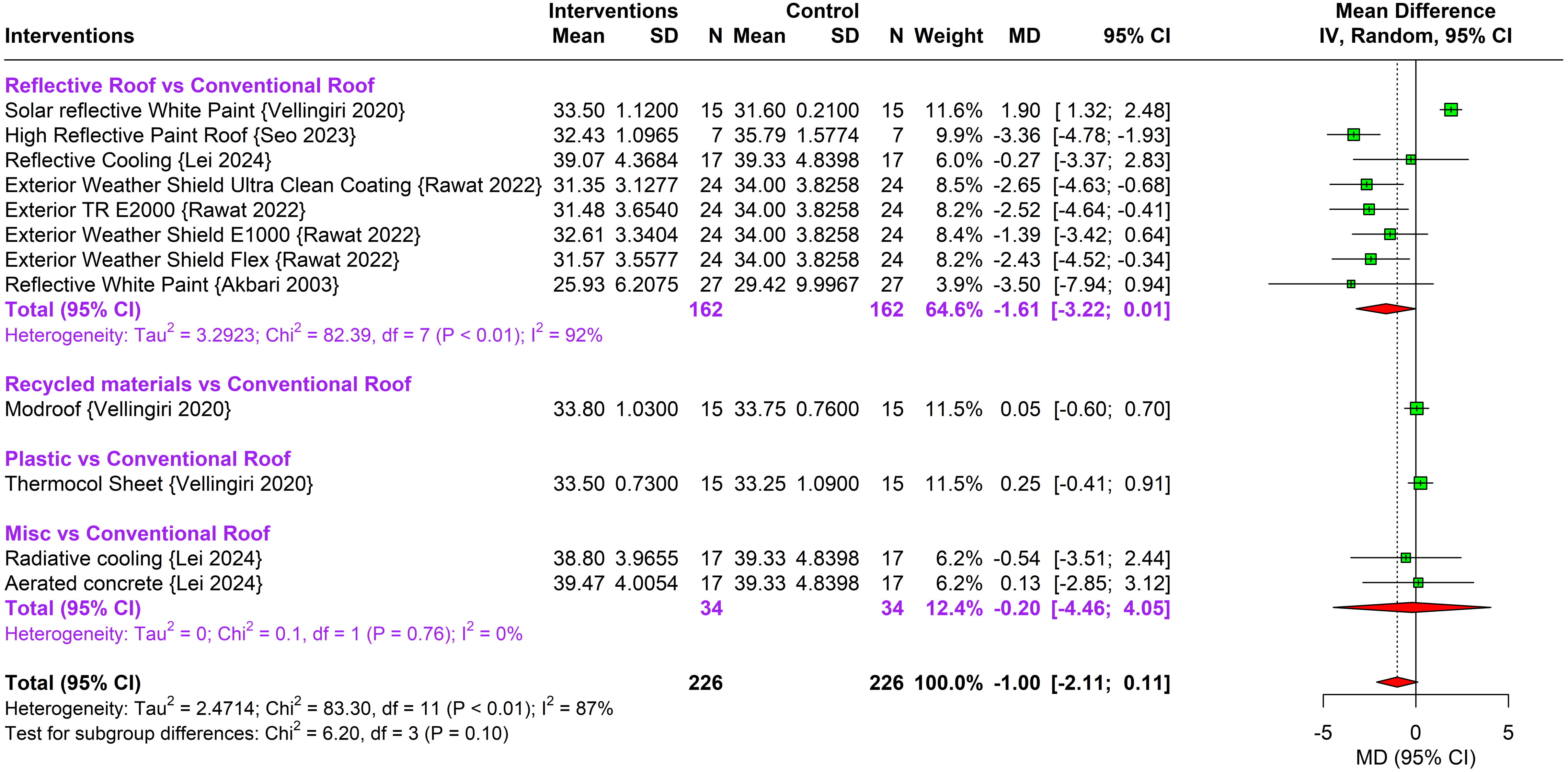

Interventions	Interventions Mean	Interventions SD	Interventions N	Control Mean	Control SD	Control N	Weight	MD	95% CI
**Reflective Roof vs Tin**									
Solar reflective White Paint {Vellingiri 2020}	33.50	1.1200	15	34.60	0.8400	15	12.0%	-1.10	[-1.81; -0.39]
**Recycled materials vs Tin**									
Modroof {Vellingiri 2020}	33.80	1.0300	15	35.80	1.0200	15	11.9%	-2.00	[-2.73; -1.27]
**Plastic vs Tin**									
Thermocol Sheet {Vellingiri 2020}	33.50	0.7300	15	35.44	0.7100	15	12.6%	-1.94	[-2.46; -1.42]
**Reflective Roof vs Asbestos**									
Solar reflective White Paint {Vellingiri 2020}	33.50	1.1200	15	32.50	0.7000	15	12.1%	1.00	[0.33; 1.67]
**Recycled materials vs Asbestos**									
Modroof {Vellingiri 2020}	33.80	1.0300	15	36.83	2.0800	15	10.3%	-3.03	[-4.20; -1.86]
**Plastic vs Asbestos**									
Thermocol Sheet {Vellingiri 2020}	33.50	0.7300	15	35.88	1.7200	15	11.2%	-2.38	[-3.33; -1.43]
**Reflective Roof vs Bare Metal**									
White gloss painted {Carrasco-Tenezaca 2021}	29.15	0.0358	120	30.69	0.0625	120	13.3%	-1.54	[-1.55; -1.53]
**Painted Roofs vs Bare Metal**									
Red oxide primer painted (rust) {Carrasco-Tenezaca 2021}	29.91	0.0708	120	30.69	0.0625	120	13.3%	-0.78	[-0.80; -0.76]
**Metal Roofs vs Conventional Steel Sheet**									
White Steel Sheet (Thermoshield coat) {Coutts 2013}	22.75	5.7523	22	23.68	6.7833	22	3.4%	-0.93	[-4.65; 2.78]
**Total (95% CI)**			**352**			**352**	**100.0%**	**-1.41**	**[-2.31; -0.51]**

Heterogeneity: $Tau^2 = 1.2230$; $Chi^2 = 4979.76$, df = 8 (P = 0); $I^2 = 100\%$

Test for subgroup differences: $Chi^2 = 4979.76$, df = 8 (P = 0)

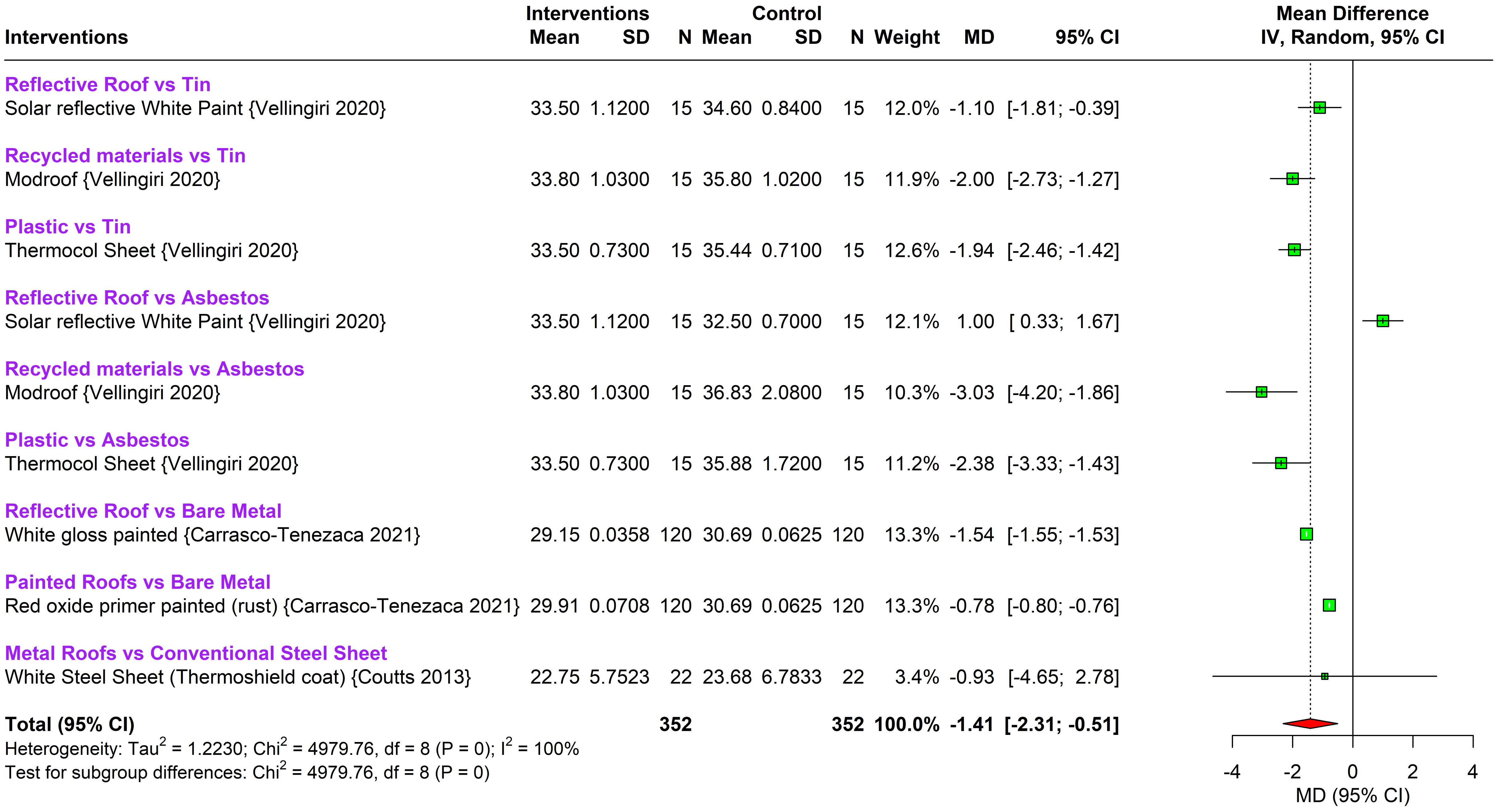

Interventions	Interventions Mean	SD	N	Control Mean	SD	N	Weight	MD	95% CI
**Reflective Roof vs Green Paint**									
White painted Roof {Lee 2015}	27.42	2.9397	6	32.42	6.7559	6	27.2%	-5.00	[-10.90; 0.90]
**Reflective Roof vs Urethane-based waterproofing material**									
CHMA silicon acrylic cool roof waterproofing material with VOCs {Park 2022}	14.71	12.1250	8766	15.27	12.7650	8766	72.8%	-0.56	[-0.93; -0.19]
**Total (95% CI)**			**8772**			**8772**	**100.0%**	**-1.76**	**[-26.87; 23.34]**

Heterogeneity: $Tau^2 = 5.3244$; $Chi^2 = 2.17$, df = 1 (P = 0.14); $I^2 = 54\%$
Test for subgroup differences: $Chi^2 = 2.17$, df = 1 (P = 0.14)

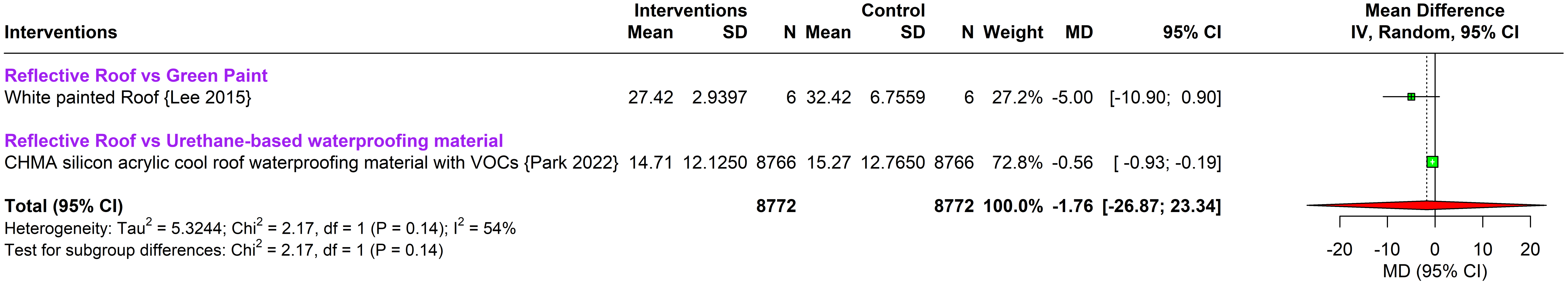

Interventions	Interventions Mean	SD	N	Control Mean	SD	N	Weight	MD	95% CI
**Various Construction vs Micro-ventilated Façade**									
ETICS {Salvalai 2019}	27.32	1.3616	45	26.66	1.0227	45	24.6%	0.66	[0.16; 1.16]
Rock wool thermal coat {Salvalai 2019}	27.62	0.8861	34	27.69	0.9974	34	24.8%	-0.08	[-0.53; 0.37]
**Total (95% CI)**			**79**			**79**	**49.4%**	**0.28**	**[-4.40; 4.96]**
Heterogeneity: $Tau^2 = 0.2129$; $Chi^2 = 4.65$, df = 1 (P = 0.03); $I^2 = 78\%$									
**Various Construction vs Light Construction**									
Very heavy construction {Kuczynski 2021}	30.59	1.2514	35	33.03	1.8706	35	23.5%	-2.44	[-3.19; -1.70]
**Various Construction vs CMU Walls, Asbestos Cement Corrugated Roof**									
CSEB Walls, RCC slab Roof {Glicksman 2016}	34.27	9.5653	15	34.67	13.6939	15	2.1%	-0.40	[-8.85; 8.05]
**Various Construction vs Energy efficient homes, but no upgrades**									
EPS Insulation {Davis 2018}	29.62	0.6260	24	29.46	0.6413	24	25.0%	0.16	[-0.20; 0.52]
**Total (95% CI)**			**153**			**153**	**100.0%**	**-0.40**	**[-2.02; 1.22]**
Heterogeneity: $Tau^2 = 1.6699$; $Chi^2 = 48.66$, df = 4 (P < 0.01); $I^2 = 92\%$									
Test for subgroup differences: $Chi^2 = 40.27$, df = 3 (P < 0.01)									

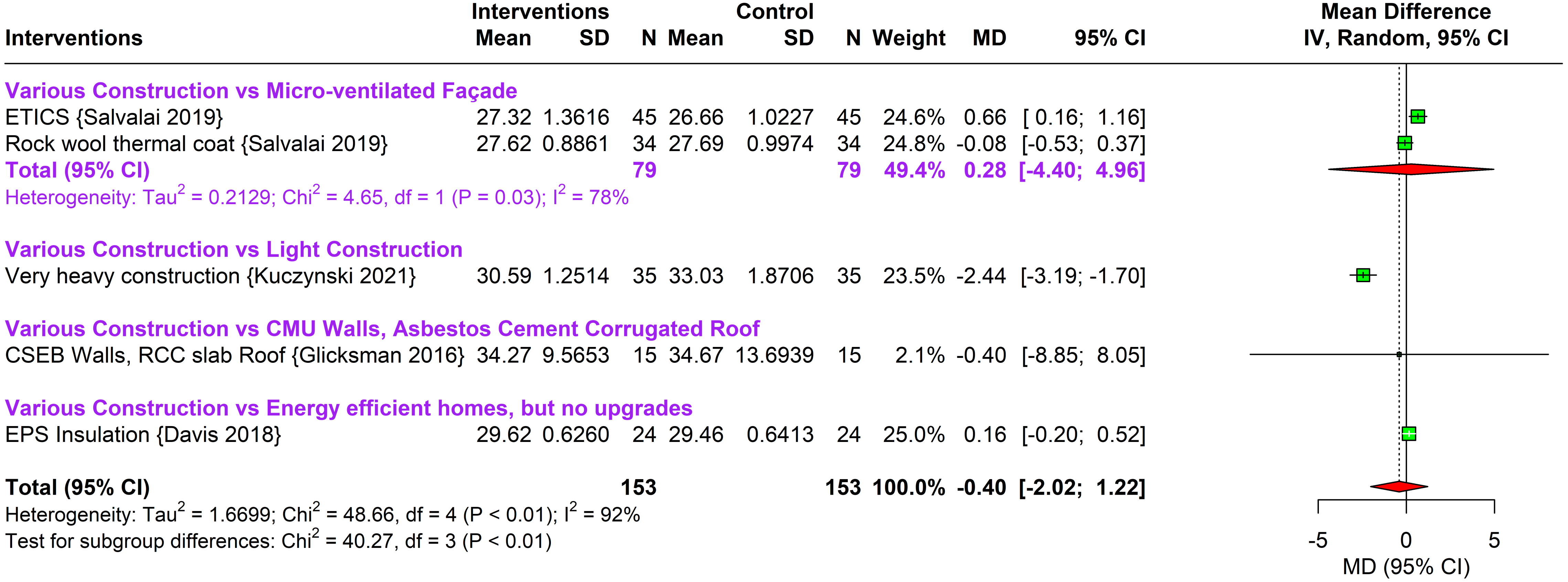

Interventions	Interventions Mean	SD	N	Control Mean	SD	N	Weight	MD	95% CI
**Ventilation vs PCM**									
Earth-to-air heat exchanger (EAHE) + Phase change material (PCM) {Guo 2022}	26.29	3.1997	7	34.14	7.1514	7	41.7%	-7.86	[-13.66; -2.05]
PCM Ventilated Window {Hu 2020}	24.49	3.8992	39	25.41	3.8982	39	58.3%	-0.92	[-2.65; 0.81]
**Total (95% CI)**			**46**			**46**	**100.0%**	**-3.81**	**[-47.25; 39.63]**

Heterogeneity: $Tau^2 = 19.2665$; $Chi^2 = 5.04$, df = 1 (P = 0.02); $I^2 = 80\%$

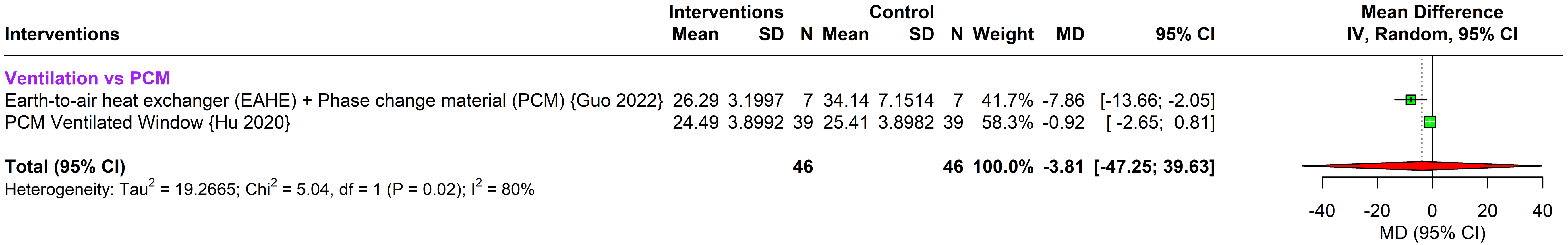

Interventions	Interventions Mean	SD	N	Control Mean	SD	N	Weight	MD	95% CI
**Ventilation vs Passive cooling, dripping ventilation, floor-level window, additional plants**									
24h ventilation, misting ventilation {Qin 2022}	31.90	2.1828	24	28.70	1.7904	24	20.0%	3.21	[2.08; 4.34]
**Ventilation vs Transpired solar cooling system**									
Transpired solar cooling system with Underground Heat Exchanger {Abed 2021}	27.08	1.0647	11	34.06	0.6516	11	20.1%	-6.98	[-7.72; -6.24]
**Ventilation vs Conventional Roof**									
Airlite Ventilation {Vellingiri 2020}	37.80	2.0300	15	33.06	1.0100	15	20.0%	4.74	[3.59; 5.89]
**Ventilation vs Tin**									
Airlite Ventilation {Vellingiri 2020}	37.80	2.0300	15	35.14	0.6400	15	20.0%	2.66	[1.58; 3.74]
**Ventilation vs Asbestos**									
Airlite Ventilation {Vellingiri 2020}	37.80	2.0300	15	35.30	1.0100	15	20.0%	2.50	[1.35; 3.65]
**Total (95% CI)**			**80**			**80**	**100.0%**	**1.21**	**[-4.60; 7.03]**

Heterogeneity: $Tau^2 = 21.7507$; $Chi^2 = 477.44$, df = 4 ($P < 0.01$); $I^2 = 99\%$
Test for subgroup differences: $Chi^2 = 477.44$, df = 4 ($P < 0.01$)

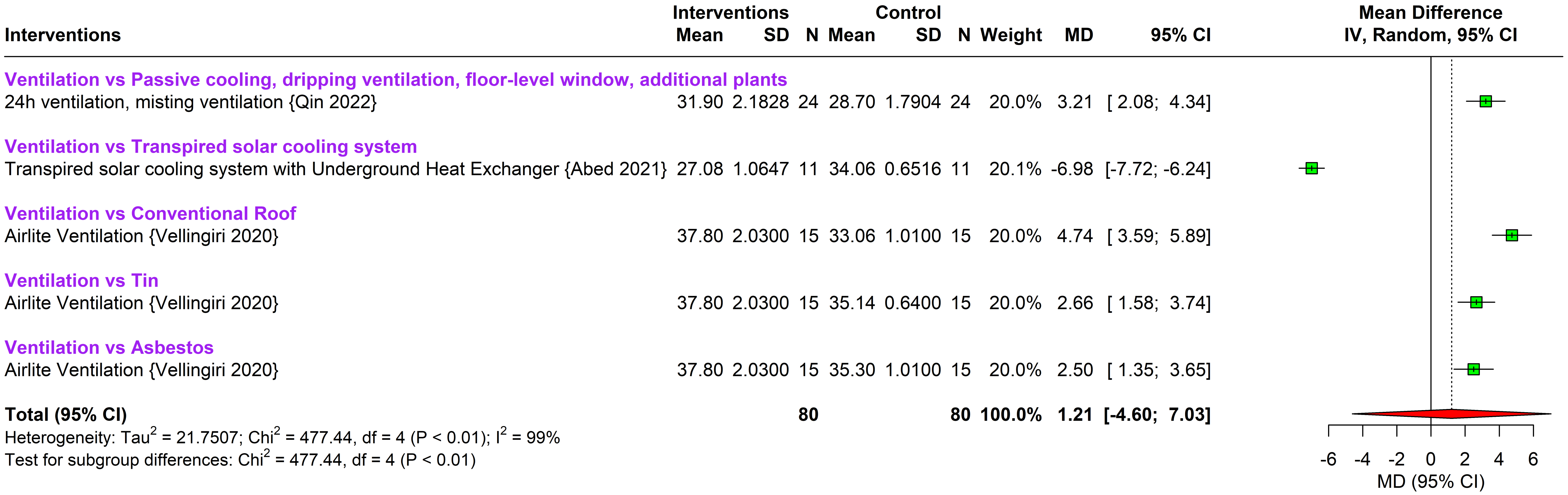

Interventions	Interventions Mean	SD	N	Control Mean	SD	N	Weight	MD	95% CI
**Various Construction & Ventilation vs Light Construction**									
Light Construction, Night Ventilation {Kuczynski 2021}	30.13	3.5838	35	33.03	1.8706	35	32.1%	-2.90	[-4.24; -1.56]
Heavy Construction, Night Ventilation {Kuczynski 2021}	28.53	1.9813	35	33.03	1.8706	35	33.7%	-4.50	[-5.40; -3.60]
Heavy Construction, Night Ventilation, Exterior Blinds {Kuczynski 2021}	25.67	1.2888	35	33.03	1.8706	35	34.2%	-7.36	[-8.11; -6.60]
**Total (95% CI)**			**105**			**105**	**100.0%**	**-4.96**	**[-10.57; 0.65]**

Heterogeneity: $Tau^2 = 4.8249$; $Chi^2 = 42.19$, df = 2 ($P < 0.01$); $I^2 = 95\%$

Mean Difference
IV, Random, 95% CI

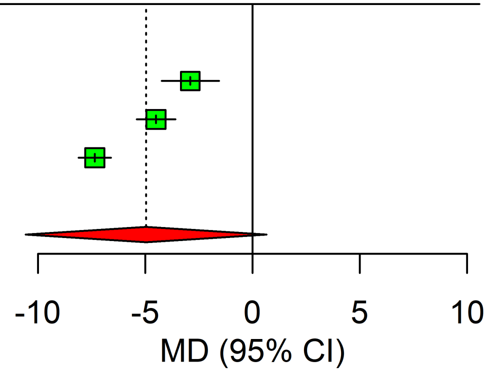

Supplement: online supplemental file 5 [file bmjph-3-2-s005.pdf]
