# Supplementary material for: Evaluation of community-based heat adaptation interventions: a systematic review
Source: BMJ Public Health. 2025 Jul 15;3(2):e002332. doi: 10.1136/bmjph-2024-002332 (PMC12273142; doi:10.1136/bmjph-2024-002332)

Annex 6 - Analysis without High RoB

Green Façade vs Barewall (removing high RoB studies) – Surface Temperature

Interventions	Interventions Mean	Interventions SD	N	Control Mean	Control SD	N	Weight	MD	95% CI
**Green Facade vs Bare wall**									
Green Facade {Safikhani 2014}	29.27	4.3900	2	29.52	7.3200	2	1.1%	-0.25	[-12.08; 11.58]
Green Facade {Widiastuti 2018}	28.25	3.8500	14	31.96	1.8600	14	2.7%	-3.71	[-5.95; -1.47]
Green Facade {Susorova 2013}	25.04	1.0300	12	25.33	1.1500	12	2.8%	-0.29	[-1.16; 0.58]
Green Facade {Susorova 2014}	28.30	3.5000	12481	29.50	5.9000	12481	2.8%	-1.20	[-1.32; -1.08]
Green Facade {Yin 2017}	36.54	2.4500	24	39.60	4.3300	24	2.7%	-3.06	[-5.05; -1.07]
Green Facade {Zhen 2022}	29.70	1.4700	10	33.60	2.1800	10	2.7%	-3.90	[-5.53; -2.27]
Green Facade {Jaafar 2015}	30.01	2.7930	240	30.63	5.9480	240	2.8%	-0.61	[-1.45; 0.22]
Green Facade {Widiastuti 2018}	29.00	2.1500	14	31.96	1.8600	14	2.7%	-2.96	[-4.45; -1.47]
Green Facade {Saify 2023}	37.33	3.8649	9	44.44	5.3470	9	2.3%	-7.11	[-11.42; -2.80]
Green Facade {Pichlhöfer 2023}	26.30	0.8000	21	27.50	0.9000	21	2.8%	-1.20	[-1.72; -0.68]
Direct Green Facade {Perini 2011}	10.39	2.2100	10	11.59	2.4100	10	2.7%	-1.20	[-3.23; 0.83]
Indirect Green Facade {Suklje 2016}	29.00	4.7200	8	41.38	9.8600	8	1.7%	-12.38	[-19.96; -4.80]
Indirect Green Facade {Lin 2019}	31.80	1.7200	10	32.25	1.8300	10	2.7%	-0.45	[-2.01; 1.11]
Indirect Green Facade {Perini 2011}	10.50	2.0500	10	13.23	2.2800	10	2.7%	-2.73	[-4.63; -0.83]
Double-skin Green Facade {Perez 2017}	18.72	5.2800	39	21.69	11.4500	39	2.4%	-2.97	[-6.93; 0.99]
Double-skin Green Facade {Coma 2017}	10.62	14.9500	13	10.04	16.6600	13	1.1%	0.58	[-11.59; 12.75]
Plant-shaded facades {Haggag 2017}	44.53	2.2900	15	48.07	6.0600	15	2.5%	-3.54	[-6.82; -0.26]
**Total (95% CI)**			**12932**			**12932**	**41.3%**	**-2.12**	**[-3.10; -1.14]**
Heterogeneity: $Tau^2 = 1.6093$; $Chi^2 = 51.84$, df = 16 ($P < 0.0001$); $I^2 = 69.1\%$									
**Living Wall vs Bare wall**									
Living Wall {Chen 2013}	32.88	2.0000	17	36.65	7.4500	17	2.5%	-3.77	[-7.44; -0.10]
Living Wall {He 2017}	29.96	1.7100	13	37.08	9.6400	13	2.1%	-7.12	[-12.44; -1.80]
Living Wall {Safikhani 2014}	28.96	4.0100	2	29.52	7.3200	2	1.1%	-0.56	[-12.13; 11.01]
Living Wall {Tudiwer 2017}	16.14	3.5800	7	25.93	4.5400	7	2.3%	-9.79	[-14.07; -5.51]
Living Wall {Wang 2011}	30.70	1.8500	22	32.69	5.0700	22	2.7%	-1.99	[-4.25; 0.27]
Living Wall {Wong 2010}	28.59	0.7000	11	30.50	8.7000	11	2.2%	-1.91	[-7.07; 3.25]
Living Wall {Olivieri 2014}	21.34	2.4100	53	34.43	3.4700	53	2.8%	-13.09	[-14.23; -11.95]
Living Wall {Coma 2017}	0.85	2.3700	13	10.04	16.6600	13	1.5%	-9.19	[-18.34; -0.04]
Living Wall {Djedjig 2015}	23.33	1.9200	9	23.61	2.7000	9	2.7%	-0.28	[-2.44; 1.88]
Living Wall {Shah 2023}	32.75	2.2546	4	35.38	1.2500	4	2.6%	-2.62	[-5.15; -0.10]
Living Wall - Festuca Glauca {Cortes 2022}	24.23	7.5000	26	23.75	6.0200	26	2.4%	0.48	[-3.22; 4.18]
Living Wall - Thymus Pulegioides {Cortes 2022}	24.76	8.2700	26	25.69	8.8000	26	2.3%	-0.93	[-5.57; 3.71]
Living Wall - Festuca Glauca {Cortes 2022}	24.23	7.5000	26	25.69	8.8000	26	2.3%	-1.46	[-5.90; 2.98]
Vertical Greenery Module System {Bianco 2016}	24.92	4.0800	13	37.42	14.4100	13	1.6%	-12.50	[-20.64; -4.36]
Green Module Wall {Gouws 2022}	22.76	2.4800	31	26.98	3.6500	31	2.7%	-4.22	[-5.77; -2.67]
Modular System {Jaafar 2015}	29.84	2.1830	240	30.63	5.9480	240	2.8%	-0.79	[-1.59; 0.02]
Geogreen module {Manso 2016}	18.72	1.7200	82	19.29	2.1000	82	2.8%	-0.57	[-1.16; 0.02]
Modular System - Nitida Lemon {Sudimac 2018}	16.28	3.4100	14	8.07	3.8300	14	2.6%	8.21	[5.52; 10.90]
Modular System - Geranium Macrorrhiuum {Sudimac 2018}	23.28	4.4800	14	8.07	3.8300	14	2.5%	15.21	[12.12; 18.30]
Modular System - Cordifolia Stock {Sudimac 2018}	17.00	6.2300	14	8.07	3.8300	14	2.4%	8.93	[5.10; 12.76]
Hanging potted pothos {Huang 2018}	30.20	3.7900	51	31.04	5.3800	51	2.7%	-0.84	[-2.65; 0.97]
Vertical Garden {Nugroho 2020}	29.64	3.5900	5	46.04	9.8000	5	1.5%	-16.40	[-25.55; -7.25]
Cork-based Living Wall {Cortes 2022}	24.76	8.2700	26	23.75	6.0200	26	2.4%	1.01	[-2.92; 4.94]
Continuous Living Wall {Cruciol-Barbosa 2023}	30.12	3.1100	56	32.50	2.9000	56	2.8%	-2.38	[-3.49; -1.27]
Felt layer {Nori 2013}	20.56	2.4000	24	25.60	10.9500	24	2.3%	-5.04	[-9.52; -0.56]
**Total (95% CI)**			**799**			**799**	**58.7%**	**-2.11**	**[-4.90; 0.68]**
Heterogeneity: $Tau^2 = 39.7557$; $Chi^2 = 656.94$, df = 24 ($P < 0.0001$); $I^2 = 96.3\%$									
**Total (95% CI)**			**13731**			**13731**	**100.0%**	**-2.27**	**[-3.95; -0.59]**
Heterogeneity: $Tau^2 = 23.6433$; $Chi^2 = 726.05$, df = 41 ($P < 0.0001$); $I^2 = 94.4\%$									
Test for subgroup differences: $Chi^2 = 0.00$, df = 1 ($P = 0.9942$)									

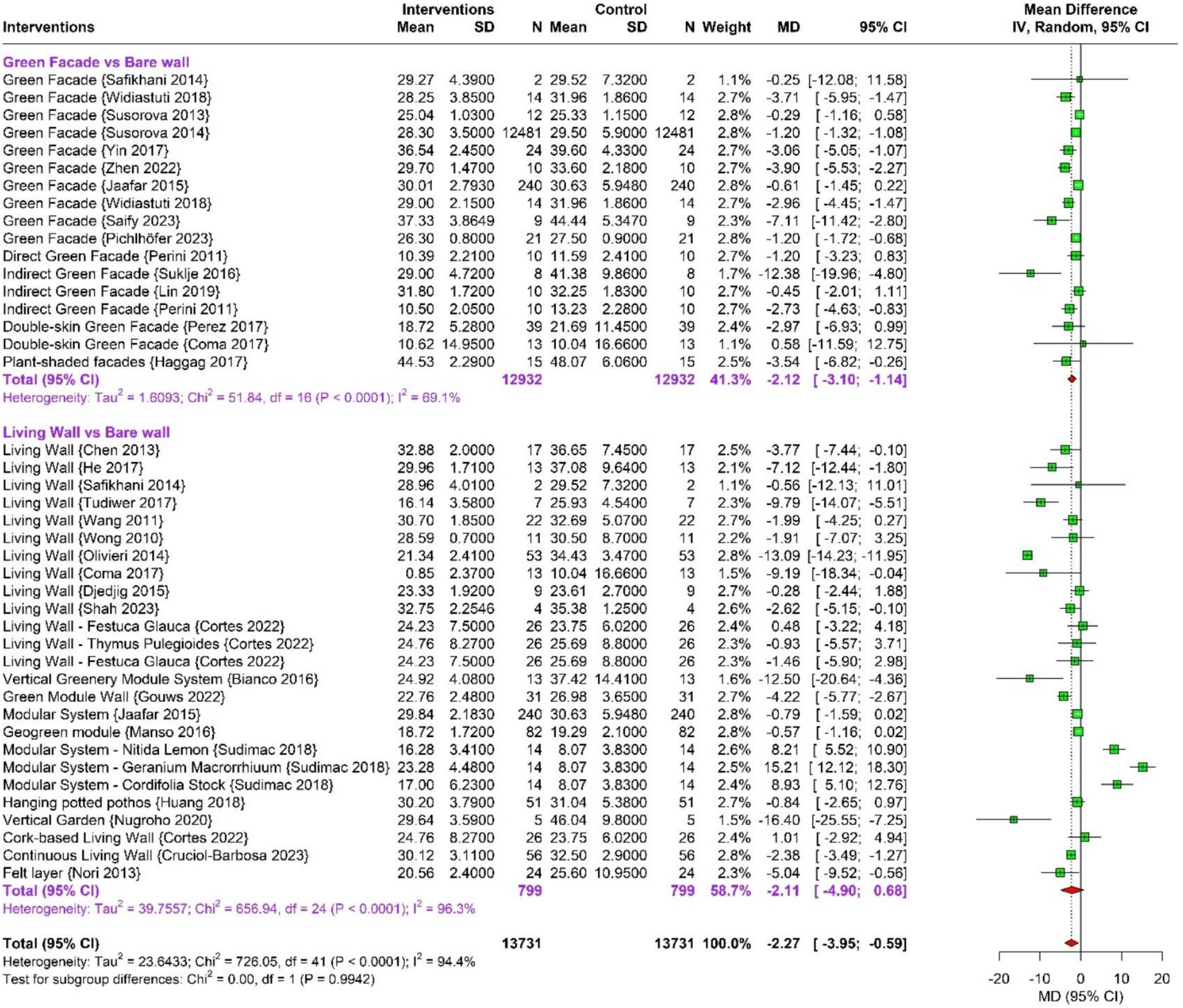

Green Façade vs Barewall (removing high RoB studies and Sudimac 2018) – Surface Temperature

	Interventions			Control					
Interventions	Mean	SD	N	Mean	SD	N	Weight	MD	95% CI
**Green Facade vs Bare wall**									
Green Facade {Safikhani 2014}	29.27	4.3900	2	29.52	7.3200	2	0.7%	-0.25	[-12.08; 11.58]
Green Facade {Widiastuti 2018}	28.25	3.8500	14	31.96	1.8600	14	3.1%	-3.71	[-5.95; -1.47]
Green Facade {Susorova 2013}	25.04	1.0300	12	25.33	1.1500	12	3.5%	-0.29	[-1.16; 0.58]
Green Facade {Susorova 2014}	28.30	3.5000	12481	29.50	5.9000	12481	3.5%	-1.20	[-1.32; -1.08]
Green Facade {Yin 2017}	36.54	2.4500	24	39.60	4.3300	24	3.2%	-3.06	[-5.05; -1.07]
Green Facade {Zhen 2022}	29.70	1.4700	10	33.60	2.1800	10	3.3%	-3.90	[-5.53; -2.27]
Green Facade {Jaafar 2015}	30.01	2.7930	240	30.63	5.9480	240	3.5%	-0.61	[-1.45; 0.22]
Green Facade {Widiastuti 2018}	29.00	2.1500	14	31.96	1.8600	14	3.3%	-2.96	[-4.45; -1.47]
Green Facade {Saify 2023}	37.33	3.8649	9	44.44	5.3470	9	2.3%	-7.11	[-11.42; -2.80]
Green Facade {Pichlhöfer 2023}	26.30	0.8000	21	27.50	0.9000	21	3.5%	-1.20	[-1.72; -0.68]
Direct Green Facade {Perini 2011}	10.39	2.2100	10	11.59	2.4100	10	3.2%	-1.20	[-3.23; 0.83]
Indirect Green Facade {Suklje 2016}	29.00	4.7200	8	41.38	9.8600	8	1.3%	-12.38	[-19.96; -4.80]
Indirect Green Facade {Lin 2019}	31.80	1.7200	10	32.25	1.8300	10	3.3%	-0.45	[-2.01; 1.11]
Indirect Green Facade {Perini 2011}	10.50	2.0500	10	13.23	2.2800	10	3.2%	-2.73	[-4.63; -0.83]
Double-skin Green Facade {Perez 2017}	18.72	5.2800	39	21.69	11.4500	39	2.4%	-2.97	[-6.93; 0.99]
Double-skin Green Facade {Coma 2017}	10.62	14.9500	13	10.04	16.6600	13	0.6%	0.58	[-11.59; 12.75]
Plant-shaded facades {Haggag 2017}	44.53	2.2900	15	48.07	6.0600	15	2.7%	-3.54	[-6.82; -0.26]
**Total (95% CI)**			**12932**			**12932**	**46.5%**	**-2.12**	**[-3.10; -1.14]**
Heterogeneity: Tau^2 = 1.6093; Chi^2 = 51.84, df = 16 (P < 0.0001); I^2 = 69.1%									
**Living Wall vs Bare wall**									
Living Wall {Chen 2013}	32.88	2.0000	17	36.65	7.4500	17	2.5%	-3.77	[-7.44; -0.10]
Living Wall {He 2017}	29.96	1.7100	13	37.08	9.6400	13	1.9%	-7.12	[-12.44; -1.80]
Living Wall {Safikhani 2014}	28.96	4.0100	2	29.52	7.3200	2	0.7%	-0.56	[-12.13; 11.01]
Living Wall {Tudiwer 2017}	16.14	3.5800	7	25.93	4.5400	7	2.3%	-9.79	[-14.07; -5.51]
Living Wall {Wang 2011}	30.70	1.8500	22	32.69	5.0700	22	3.1%	-1.99	[-4.25; 0.27]
Living Wall {Wong 2010}	28.59	0.7000	11	30.50	8.7000	11	2.0%	-1.91	[-7.07; 3.25]
Living Wall {Olivieri 2014}	21.34	2.4100	53	34.43	3.4700	53	3.4%	-13.09	[-14.23; -11.95]
Living Wall {Coma 2017}	0.85	2.3700	13	10.04	16.6600	13	1.0%	-9.19	[-18.34; -0.04]
Living Wall {Djedjig 2015}	23.33	1.9200	9	23.61	2.7000	9	3.1%	-0.28	[-2.44; 1.88]
Living Wall {Shah 2023}	32.75	2.2546	4	35.38	1.2500	4	3.0%	-2.62	[-5.15; -0.10]
Living Wall - Festuca Glauca {Cortes 2022}	24.23	7.5000	26	23.75	6.0200	26	2.5%	0.48	[-3.22; 4.18]
Living Wall - Thymus Pulegioides {Cortes 2022}	24.76	8.2700	26	25.69	8.8000	26	2.1%	-0.93	[-5.57; 3.71]
Living Wall - Festuca Glauca {Cortes 2022}	24.23	7.5000	26	25.69	8.8000	26	2.2%	-1.46	[-5.90; 2.98]
Vertical Greenery Module System {Bianco 2016}	24.92	4.0800	13	37.42	14.4100	13	1.2%	-12.50	[-20.64; -4.36]
Green Module Wall {Gouws 2022}	22.76	2.4800	31	26.98	3.6500	31	3.3%	-4.22	[-5.77; -2.67]
Modular System {Jaafar 2015}	29.84	2.1830	240	30.63	5.9480	240	3.5%	-0.79	[-1.59; 0.02]
Geogreen module {Manso 2016}	18.72	1.7200	82	19.29	2.1000	82	3.5%	-0.57	[-1.16; 0.02]
Hanging potted pothos {Huang 2018}	30.20	3.7900	51	31.04	5.3800	51	3.2%	-0.84	[-2.65; 0.97]
Vertical Garden {Nugroho 2020}	29.64	3.5900	5	46.04	9.8000	5	1.0%	-16.40	[-25.55; -7.25]
Cork-based Living Wall {Cortes 2022}	24.76	8.2700	26	23.75	6.0200	26	2.4%	1.01	[-2.92; 4.94]
Continuous Living Wall {Cruciol-Barbosa 2023}	30.12	3.1100	56	32.50	2.9000	56	3.4%	-2.38	[-3.49; -1.27]
Felt layer {Nori 2013}	20.56	2.4000	24	25.60	10.9500	24	2.2%	-5.04	[-9.52; -0.56]
**Total (95% CI)**			**757**			**757**	**53.5%**	**-3.72**	**[-5.71; -1.73]**
Heterogeneity: Tau^2 = 14.5706; Chi^2 = 443.07, df = 21 (P < 0.0001); I^2 = 95.3%									
**Total (95% CI)**			**13689**			**13689**	**100.0%**	**-3.10**	**[-4.26; -1.93]**
Heterogeneity: Tau^2 = 8.6071; Chi^2 = 540.16, df = 38 (P < 0.0001); I^2 = 93.0%									
Test for subgroup differences: Chi^2 = 2.28, df = 1 (P = 0.1310)									

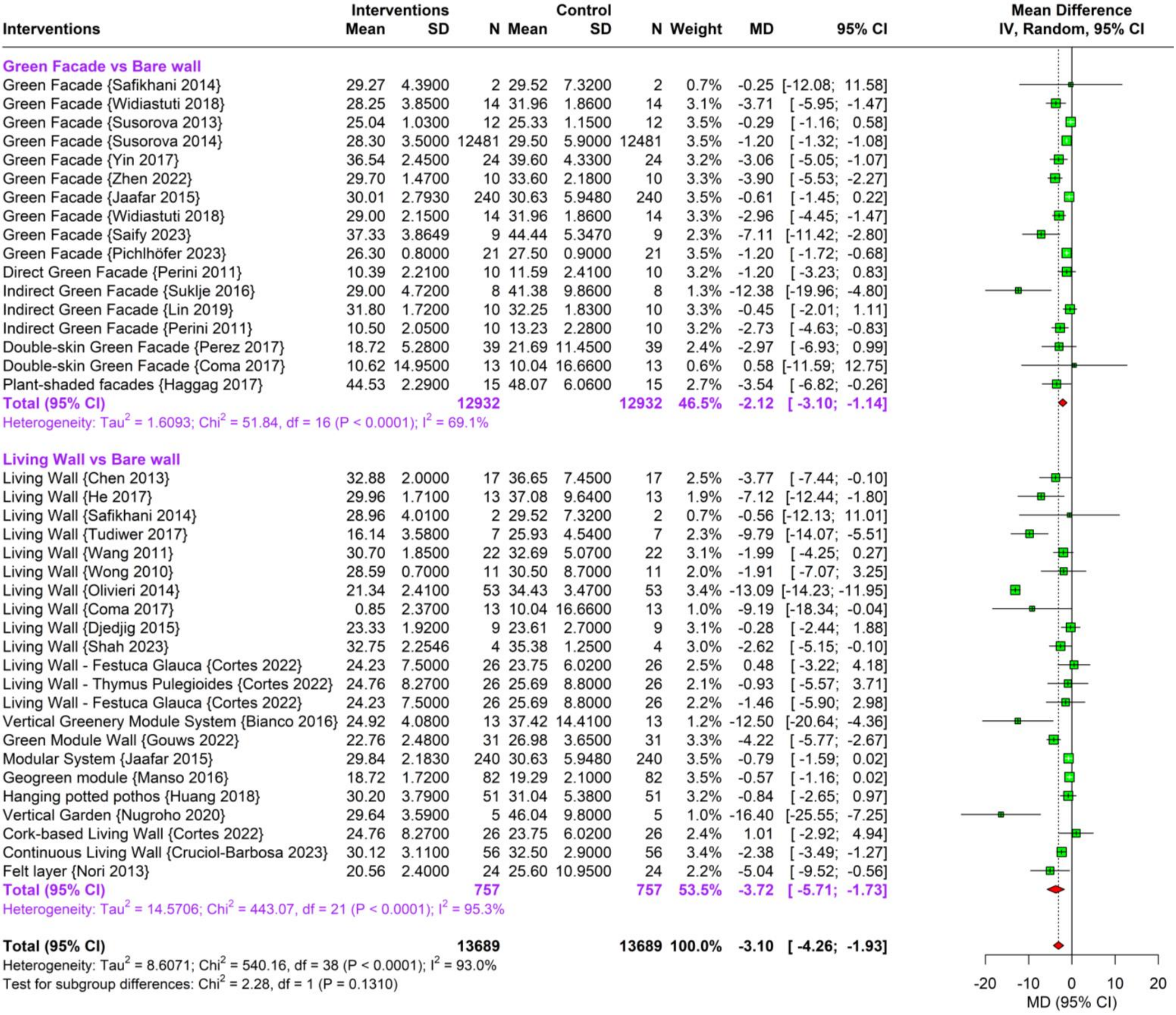

Green Façade vs Barewall (removing high RoB studies) – Indoor Temperature

Interventions	Interventions Mean	Interventions SD	N	Control Mean	Control SD	N	Weight	MD	95% CI
**Green Facade vs Bare wall**									
Green Facade {Safikhani 2014}	28.96	4.2064	24	30.46	6.3724	24	3.8%	-1.50	[-4.55; 1.55]
Green Facade {Widiastuti 2018}	28.14	2.3487	14	31.64	3.0536	14	5.0%	-3.50	[-5.52; -1.48]
Green Facade {Haggag 2017}	36.20	2.6780	15	37.60	6.6526	15	3.3%	-1.40	[-5.03; 2.23]
Green Facade {Susorova 2013}	23.41	2.0931	8	24.24	2.2557	8	4.8%	-0.82	[-2.96; 1.31]
Green Facade {Susorova 2014}	24.80	1.1000	12481	25.30	0.7000	12481	6.5%	-0.50	[-0.52; -0.48]
Green Facade {Widiastuti 2018}	24.21	1.8051	14	29.64	2.5603	14	5.4%	-5.43	[-7.07; -3.79]
Green Facade A {Saify 2023}	35.19	2.5062	8	35.97	2.8361	8	4.3%	-0.78	[-3.40; 1.84]
Green Façade B {Saify 2023}	29.50	4.4159	8	31.81	5.0351	8	2.5%	-2.31	[-6.95; 2.33]
Double-skin Green Façade {Perez 2017}	27.76	0.8794	25	30.72	1.2754	25	6.3%	-2.96	[-3.57; -2.35]
Double-skin Green Façade {Coma 2017}	4.85	1.2810	13	4.38	0.7679	13	6.2%	0.46	[-0.35; 1.27]
**Total (95% CI)**			**12610**			**12610**	**48.1%**	**-1.85**	**[-3.18; -0.52]**
Heterogeneity: $Tau^2 = 2.9287$; $Chi^2 = 112.8$, df = 9 ($P < 0.0001$); $I^2 = 92\%$									
**Living Wall vs Bare wall**									
Geogreen module {Manso 2016}	18.70	3.1641	76	19.27	7.0571	76	5.3%	-0.57	[-2.31; 1.17]
Living Wall {Chen 2013}	33.59	1.9059	17	36.12	4.5261	17	4.6%	-2.53	[-4.86; -0.19]
Living Wall {He 2017}	33.33	3.2004	12	33.58	3.3967	12	4.3%	-0.25	[-2.89; 2.39]
Living Wall {Safikhani 2014}	28.62	3.8427	24	30.46	6.3724	24	3.9%	-1.83	[-4.81; 1.14]
Living Wall {Olivieri 2014}	24.55	2.0245	53	31.22	2.2861	53	6.2%	-6.67	[-7.49; -5.85]
Living Wall {Coma 2017}	2.85	0.6887	13	4.38	0.7679	13	6.4%	-1.54	[-2.10; -0.98]
Living Wall {Djedjig 2015}	26.62	0.8561	12	26.71	1.3561	12	6.1%	-0.08	[-0.99; 0.82]
Living Wall {Shah 2023}	24.12	1.5478	4	28.50	1.2247	4	5.1%	-4.38	[-6.31; -2.44]
Continuous Living Wall {Cruciol-Barbosa 2023}	25.14	1.0336	58	26.81	2.6054	58	6.3%	-1.67	[-2.39; -0.95]
Felt layer {Nori 2013}	15.79	0.4149	24	17.00	0.0000	24	0.0%	-1.21	
Vertical greening systems {Yang 2023}	25.33	3.2787	9	25.78	3.1929	9	3.9%	-0.44	[-3.43; 2.55]
**Total (95% CI)**			**302**			**302**	**51.9%**	**-2.08**	**[-3.63; -0.53]**
Heterogeneity: $Tau^2 = 4.2446$; $Chi^2 = 153.54$, df = 9 ($P < 0.0001$); $I^2 = 94.1\%$									
**Total (95% CI)**			**12912**			**12912**	**100.0%**	**-1.98**	**[-2.90; -1.06]**
Heterogeneity: $Tau^2 = 3.4372$; $Chi^2 = 371.43$, df = 19 ($P < 0.0001$); $I^2 = 94.9\%$									
Test for subgroup differences: $Chi^2 = 0.07$, df = 1 ($P = 0.7985$)									

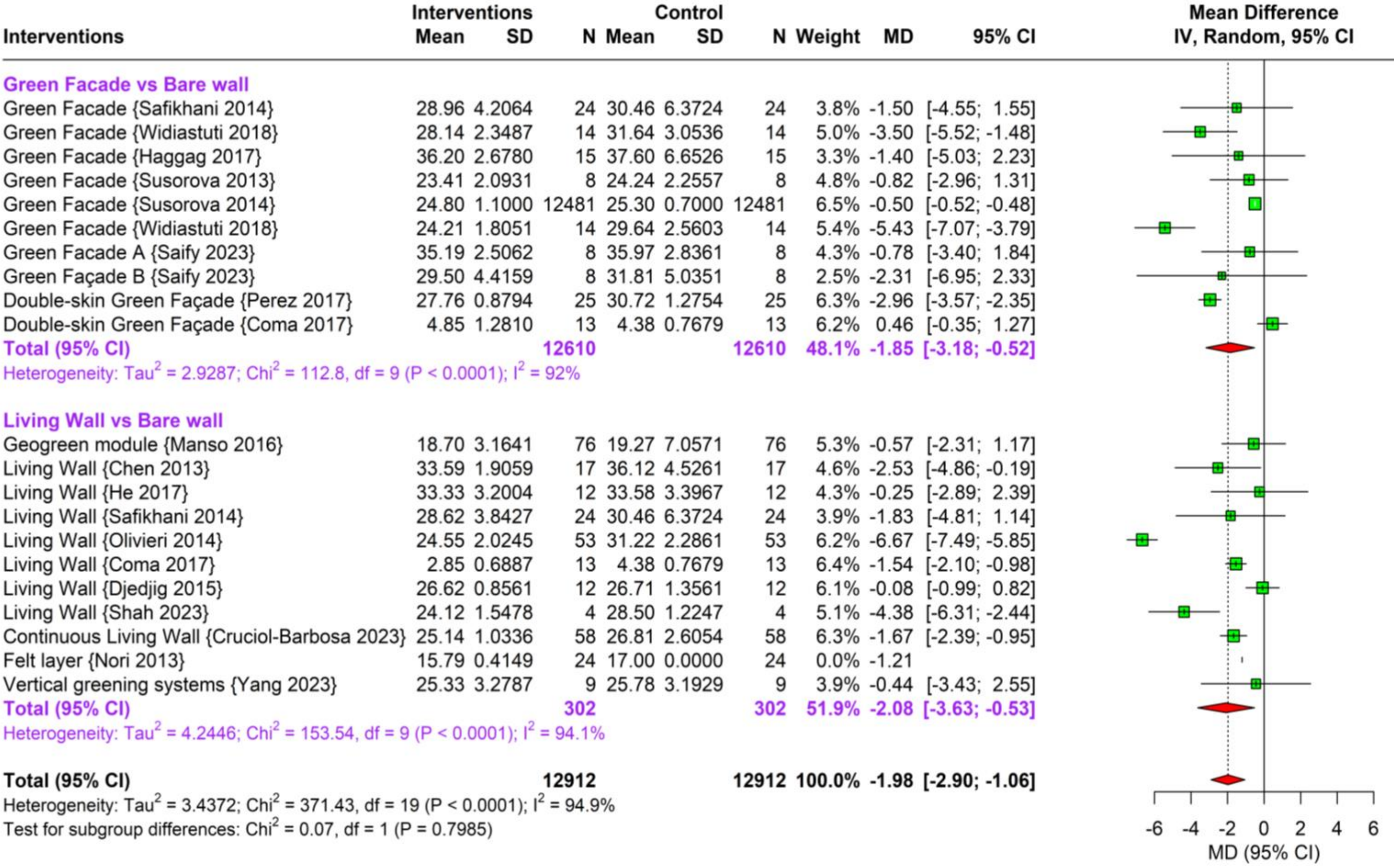

Shade vs No Shade (removing high RoB studies) – Indoor Temperature

Interventions	Interventions Mean	Interventions SD	N	Control Mean	Control SD	N	Weight	MD	95% CI
**Shade Sail vs Unshaded**									
HDPE Plastic Sheet {Jareemit 2022}	31.67	3.7739	12	32.38	4.3543	12	4.2%	-0.71	[-3.97; 2.55]
Mylar Rescue Blanket {Karanja 2023}	39.70	5.7600	865	39.50	6.4600	865	13.8%	0.20	[-0.38; 0.78]
White Cotton Bedsheet {Karanja 2023}	40.60	7.0400	865	39.50	6.4600	865	13.6%	1.10	[0.46; 1.74]
Blue Tarp {Karanja 2023}	41.90	8.8500	865	39.50	6.4600	865	13.2%	2.40	[1.67; 3.13]
Sunbrella Fabric {Karanja 2023}	41.80	8.2800	865	39.50	6.4600	865	13.4%	2.30	[1.60; 3.00]
Aluminium Foil {Karanja 2023}	39.50	5.2200	865	39.50	6.4600	865	13.9%	0.00	[-0.55; 0.55]
**Shade Structure vs Unshaded**									
Egg-crate Shading device {Calama-Gonzalez 2019}	0.79	21.1333	90	0.85	0.7756	90	2.7%	-0.06	[-4.42; 4.31]
Shaded {Lee 2020}	32.43	1.5549	14	32.79	1.4769	14	11.5%	-0.36	[-1.48; 0.77]
Shaded Roof {Zhang 2021}	23.35	2.7600	17	23.53	5.1005	17	5.3%	-0.18	[-2.93; 2.58]
**Misc vs Unshaded**									
Galvanized Steel Sheet {Jareemit 2022}	31.58	3.8009	12	32.38	4.3543	12	4.2%	-0.79	[-4.06; 2.48]
Polycarbonate Sheet {Jareemit 2022}	31.75	3.7203	12	32.38	4.3543	12	4.2%	-0.62	[-3.87; 2.62]
**Total (95% CI)**			**4482**			**4482**	**100.0%**	**0.66**	**[-0.14; 1.46]**

Heterogeneity: $Tau^2 = 1.0805$; $Chi^2 = 56.10$, df = 10 ($P < 0.0001$); $I^2 = 82.2\%$

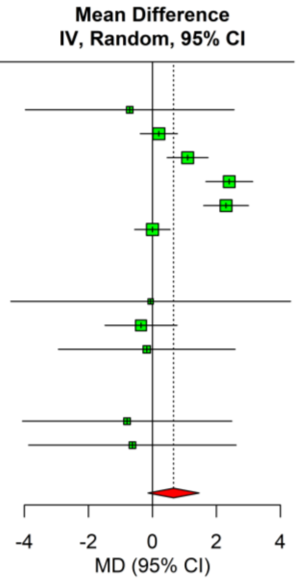

Supplement: online supplemental file 7 [file bmjph-3-2-s007.pdf]
