# Supplementary material for: Evaluation of community-based heat adaptation interventions: a systematic review
Source: BMJ Public Health. 2025 Jul 15;3(2):e002332. doi: 10.1136/bmjph-2024-002332 (PMC12273142; doi:10.1136/bmjph-2024-002332)

Annex 7 - Sensitivity analysis

Green Walls vs Bare Walls – Surface Temperature

Interventions	Interventions Mean	Interventions SD	N	Control Mean	Control SD	N	Weight	MD	95% CI
**Green Facade vs Bare wall**									
Green Facade {Safikhani 2014}	29.27	4.3900	2	29.52	7.3200	2	1.1%	-0.25	[-12.08; 11.58]
Green Facade {Widiastuti 2018}	28.25	3.8500	14	31.96	1.8600	14	2.6%	-3.71	[-5.95; -1.47]
Green Facade {Susorova 2013}	25.04	1.0300	12	25.33	1.1500	12	2.7%	-0.29	[-1.16; 0.58]
Green Facade {Susorova 2014}	28.30	3.5000	12481	29.50	5.9000	12481	2.7%	-1.20	[-1.32; -1.08]
Green Facade {Yin 2017}	36.54	2.4500	24	39.60	4.3300	24	2.6%	-3.06	[-5.05; -1.07]
Green Facade {Zhen 2022}	29.70	1.4700	10	33.60	2.1800	10	2.7%	-3.90	[-5.53; -2.27]
Green Facade {Jaafar 2015}	30.01	2.7930	240	30.63	5.9480	240	2.7%	-0.61	[-1.45; 0.22]
Green Facade {Widiastuti 2018}	29.00	2.1500	14	31.96	1.8600	14	2.7%	-2.96	[-4.45; -1.47]
Green Facade {Saify 2023}	37.33	3.8649	9	44.44	5.3470	9	2.3%	-7.11	[-11.42; -2.80]
Green Facade {Pichlhöfer 2023}	26.30	0.8000	21	27.50	0.9000	21	2.7%	-1.20	[-1.72; -0.68]
Direct Green Facade {Perini 2011}	10.39	2.2100	10	11.59	2.4100	10	2.6%	-1.20	[-3.23; 0.83]
Indirect Green Facade {Suklje 2016}	29.00	4.7200	8	41.38	9.8600	8	1.7%	-12.38	[-19.96; -4.80]
Indirect Green Facade {Lin 2019}	31.80	1.7200	10	32.25	1.8300	10	2.7%	-0.45	[-2.01; 1.11]
Indirect Green Facade {Perini 2011}	10.50	2.0500	10	13.23	2.2800	10	2.6%	-2.73	[-4.63; -0.83]
Double-skin Green Facade {Perez 2017}	18.72	5.2800	39	21.69	11.4500	39	2.3%	-2.97	[-6.93; 0.99]
Double-skin Green Facade {Coma 2017}	10.62	14.9500	13	10.04	16.6600	13	1.0%	0.58	[-11.59; 12.75]
Plant-shaded facades {Haggag 2017}	44.53	2.2900	15	48.07	6.0600	15	2.4%	-3.54	[-6.82; -0.26]
**Total (95% CI)**			**12932**			**12932**	**40.2%**	**-2.12**	**[-3.10; -1.14]**
Heterogeneity: $Tau^2 = 1.6093$; $Chi^2 = 51.84$, df = 16 ($P < 0.01$); $I^2 = 69\%$									
**Living Wall vs Bare wall**									
Living Wall {Chen 2013}	32.88	2.0000	17	36.65	7.4500	17	2.4%	-3.77	[-7.44; -0.10]
Living Wall {He 2017}	29.96	1.7100	13	37.08	9.6400	13	2.1%	-7.12	[-12.44; -1.80]
Living Wall {Safikhani 2014}	28.96	4.0100	2	29.52	7.3200	2	1.1%	-0.56	[-12.13; 11.01]
Living Wall {Tudiwer 2017}	16.14	3.5800	7	25.93	4.5400	7	2.3%	-9.79	[-14.07; -5.51]
Living Wall {Wang 2011}	30.70	1.8500	22	32.69	5.0700	22	2.6%	-1.99	[-4.25; 0.27]
Living Wall {Wong 2010}	28.59	0.7000	11	30.50	8.7000	11	2.1%	-1.91	[-7.07; 3.25]
Living Wall {Haggag 2014}	49.39	0.4300	7	55.96	0.4700	7	2.7%	-6.57	[-7.04; -6.10]
Living Wall {Olivieri 2014}	21.34	2.4100	53	34.43	3.4700	53	2.7%	-13.09	[-14.23; -11.95]
Living Wall {Coma 2017}	0.85	2.3700	13	10.04	16.6600	13	1.4%	-9.19	[-18.34; -0.04]
Living Wall {Djedjig 2015}	23.33	1.9200	9	23.61	2.7000	9	2.6%	-0.28	[-2.44; 1.88]
Living Wall {Shah 2023}	32.75	2.2546	4	35.38	1.2500	4	2.6%	-2.62	[-5.15; -0.10]
Living Wall - Festuca Glauca {Cortes 2022}	24.23	7.5000	26	23.75	6.0200	26	2.4%	0.48	[-3.22; 4.18]
Living Wall - Thymus Pulegioides {Cortes 2022}	24.76	8.2700	26	25.69	8.8000	26	2.2%	-0.93	[-5.57; 3.71]
Living Wall - Festuca Glauca {Cortes 2022}	24.23	7.5000	26	25.69	8.8000	26	2.2%	-1.46	[-5.90; 2.98]
Vertical Greenery Module System {Bianco 2016}	24.92	4.0800	13	37.42	14.4100	13	1.6%	-12.50	[-20.64; -4.36]
Green Module Wall {Gouws 2022}	22.76	2.4800	31	26.98	3.6500	31	2.7%	-4.22	[-5.77; -2.67]
Modular System {Jaafar 2015}	29.84	2.1830	240	30.63	5.9480	240	2.7%	-0.79	[-1.59; 0.02]
Geogreen module {Manso 2016}	18.72	1.7200	82	19.29	2.1000	82	2.7%	-0.57	[-1.16; 0.02]
Modular System - Nitida Lemon {Sudimac 2018}	16.28	3.4100	14	8.07	3.8300	14	2.5%	8.21	[5.52; 10.90]
Modular System - Geranium Macrorrhiuum {Sudimac 2018}	23.28	4.4800	14	8.07	3.8300	14	2.5%	15.21	[12.12; 18.30]
Modular System - Cordifolia Stock {Sudimac 2018}	17.00	6.2300	14	8.07	3.8300	14	2.4%	8.93	[5.10; 12.76]
Hanging potted pothos {Huang 2018}	30.20	3.7900	51	31.04	5.3800	51	2.6%	-0.84	[-2.65; 0.97]
Vertical Garden {Nugroho 2020}	29.64	3.5900	5	46.04	9.8000	5	1.4%	-16.40	[-25.55; -7.25]
Cork-based Living Wall {Cortes 2022}	24.76	8.2700	26	23.75	6.0200	26	2.3%	1.01	[-2.92; 4.94]
Continuous Living Wall {Cruciol-Barbosa 2023}	30.12	3.1100	56	32.50	2.9000	56	2.7%	-2.38	[-3.49; -1.27]
Felt layer {Nori 2013}	20.56	2.4000	24	25.60	10.9500	24	2.2%	-5.04	[-9.52; -0.56]
**Total (95% CI)**			**806**			**806**	**59.8%**	**-2.29**	**[-4.99; 0.40]**
Heterogeneity: $Tau^2 = 38.8103$; $Chi^2 = 889.22$, df = 25 ($P < 0.01$); $I^2 = 97\%$									
**Total (95% CI)**			**13738**			**13738**	**100.0%**	**-2.39**	**[-4.03; -0.74]**
Heterogeneity: $Tau^2 = 23.5085$; $Chi^2 = 1182.20$, df = 42 ($P < 0.01$); $I^2 = 96\%$									
Test for subgroup differences: $Chi^2 = 0.02$, df = 1 ($P = 0.90$)									

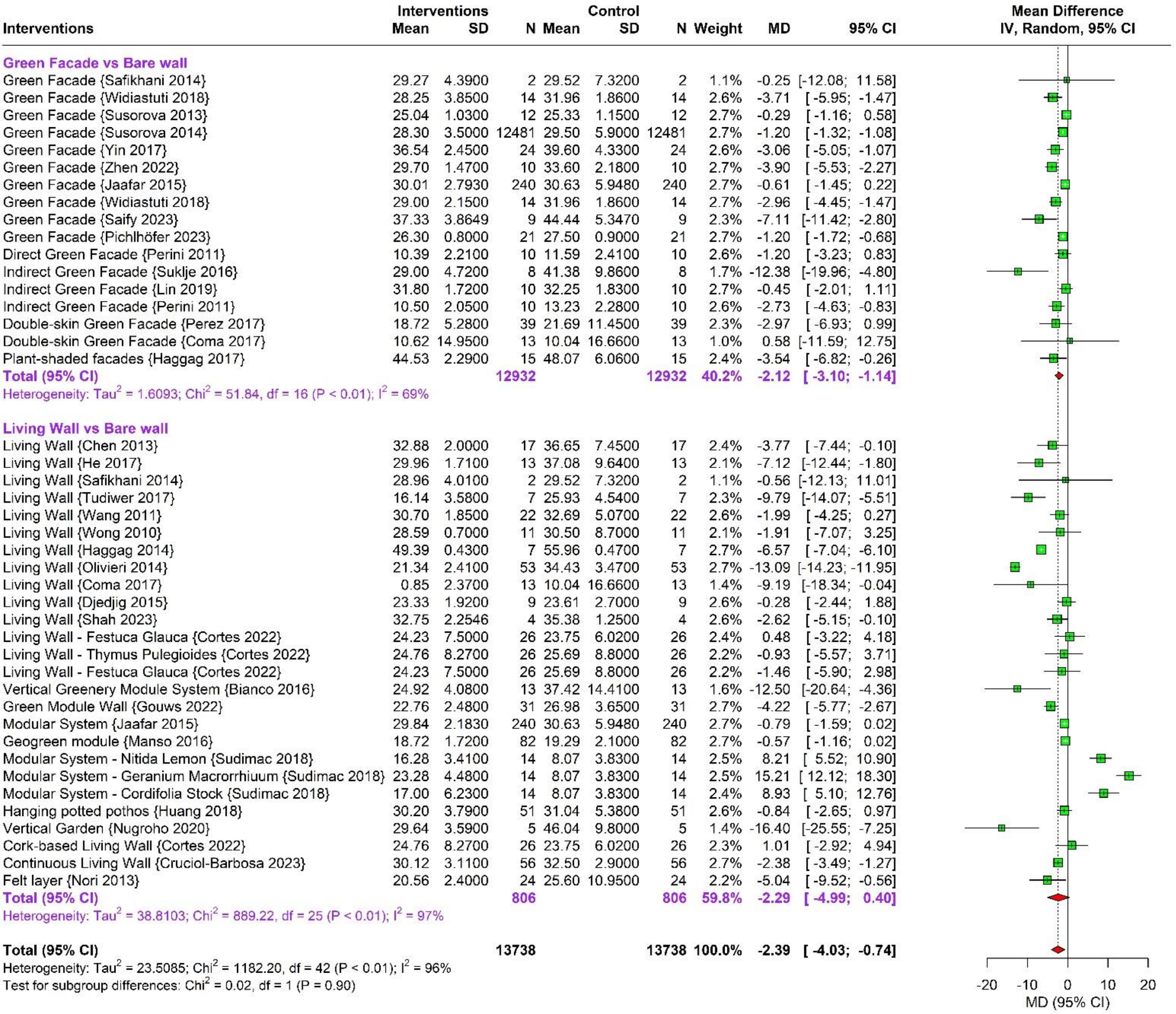

Green Walls vs Bare Walls – Surface Temperature (without Sudimac 2018)

Interventions	Interventions Mean	Interventions SD	N	Control Mean	Control SD	N	Weight	MD	95% CI
**Green Facade vs Bare wall**									
Green Facade {Safikhani 2014}	29.27	4.3900	2	29.52	7.3200	2	0.7%	-0.25	[-12.08; 11.58]
Green Facade {Widiastuti 2018}	28.25	3.8500	14	31.96	1.8600	14	3.0%	-3.71	[-5.95; -1.47]
Green Facade {Susorova 2013}	25.04	1.0300	12	25.33	1.1500	12	3.3%	-0.29	[-1.16; 0.58]
Green Facade {Susorova 2014}	28.30	3.5000	12481	29.50	5.9000	12481	3.4%	-1.20	[-1.32; -1.08]
Green Facade {Yin 2017}	36.54	2.4500	24	39.60	4.3300	24	3.1%	-3.06	[-5.05; -1.07]
Green Facade {Zhen 2022}	29.70	1.4700	10	33.60	2.1800	10	3.2%	-3.90	[-5.53; -2.27]
Green Facade {Jaafar 2015}	30.01	2.7930	240	30.63	5.9480	240	3.3%	-0.61	[-1.45; 0.22]
Green Facade {Widiastuti 2018}	29.00	2.1500	14	31.96	1.8600	14	3.2%	-2.96	[-4.45; -1.47]
Green Facade {Saify 2023}	37.33	3.8649	9	44.44	5.3470	9	2.2%	-7.11	[-11.42; -2.80]
Green Facade {Pichlhöfer 2023}	26.30	0.8000	21	27.50	0.9000	21	3.4%	-1.20	[-1.72; -0.68]
Direct Green Facade {Perini 2011}	10.39	2.2100	10	11.59	2.4100	10	3.0%	-1.20	[-3.23; 0.83]
Indirect Green Facade {Suklje 2016}	29.00	4.7200	8	41.38	9.8600	8	1.3%	-12.38	[-19.96; -4.80]
Indirect Green Facade {Lin 2019}	31.80	1.7200	10	32.25	1.8300	10	3.2%	-0.45	[-2.01; 1.11]
Indirect Green Facade {Perini 2011}	10.50	2.0500	10	13.23	2.2800	10	3.1%	-2.73	[-4.63; -0.83]
Double-skin Green Facade {Perez 2017}	18.72	5.2800	39	21.69	11.4500	39	2.3%	-2.97	[-6.93; 0.99]
Double-skin Green Facade {Coma 2017}	10.62	14.9500	13	10.04	16.6600	13	0.6%	0.58	[-11.59; 12.75]
Plant-shaded facades {Haggag 2017}	44.53	2.2900	15	48.07	6.0600	15	2.6%	-3.54	[-6.82; -0.26]
**Total (95% CI)**			**12932**			**12932**	**44.8%**	**-2.12**	**[-3.10; -1.14]**
Heterogeneity: $Tau^2 = 1.6093$; $Chi^2 = 51.84$, df = 16 ($P < 0.01$); $I^2 = 69\%$									
**Living Wall vs Bare wall**									
Living Wall {Chen 2013}	32.88	2.0000	17	36.65	7.4500	17	2.4%	-3.77	[-7.44; -0.10]
Living Wall {He 2017}	29.96	1.7100	13	37.08	9.6400	13	1.9%	-7.12	[-12.44; -1.80]
Living Wall {Safikhani 2014}	28.96	4.0100	2	29.52	7.3200	2	0.7%	-0.56	[-12.13; 11.01]
Living Wall {Tudiwer 2017}	16.14	3.5800	7	25.93	4.5400	7	2.2%	-9.79	[-14.07; -5.51]
Living Wall {Wang 2011}	30.70	1.8500	22	32.69	5.0700	22	3.0%	-1.99	[-4.25; 0.27]
Living Wall {Wong 2010}	28.59	0.7000	11	30.50	8.7000	11	1.9%	-1.91	[-7.07; 3.25]
Living Wall {Haggag 2014}	49.39	0.4300	7	55.96	0.4700	7	3.4%	-6.57	[-7.04; -6.10]
Living Wall {Olivieri 2014}	21.34	2.4100	53	34.43	3.4700	53	3.3%	-13.09	[-14.23; -11.95]
Living Wall {Coma 2017}	0.85	2.3700	13	10.04	16.6600	13	1.0%	-9.19	[-18.34; -0.04]
Living Wall {Djedjig 2015}	23.33	1.9200	9	23.61	2.7000	9	3.0%	-0.28	[-2.44; 1.88]
Living Wall {Shah 2023}	32.75	2.2546	4	35.38	1.2500	4	2.9%	-2.62	[-5.15; -0.10]
Living Wall - Festuca Glauca {Cortes 2022}	24.23	7.5000	26	23.75	6.0200	26	2.4%	0.48	[-3.22; 4.18]
Living Wall - Thymus Pulegioides {Cortes 2022}	24.76	8.2700	26	25.69	8.8000	26	2.1%	-0.93	[-5.57; 3.71]
Living Wall - Festuca Glauca {Cortes 2022}	24.23	7.5000	26	25.69	8.8000	26	2.2%	-1.46	[-5.90; 2.98]
Vertical Greenery Module System {Bianco 2016}	24.92	4.0800	13	37.42	14.4100	13	1.2%	-12.50	[-20.64; -4.36]
Green Module Wall {Gouws 2022}	22.76	2.4800	31	26.98	3.6500	31	3.2%	-4.22	[-5.77; -2.67]
Modular System {Jaafar 2015}	29.84	2.1830	240	30.63	5.9480	240	3.3%	-0.79	[-1.59; 0.02]
Geogreen module {Manso 2016}	18.72	1.7200	82	19.29	2.1000	82	3.4%	-0.57	[-1.16; 0.02]
Hanging potted pothos {Huang 2018}	30.20	3.7900	51	31.04	5.3800	51	3.1%	-0.84	[-2.65; 0.97]
Vertical Garden {Nugroho 2020}	29.64	3.5900	5	46.04	9.8000	5	1.0%	-16.40	[-25.55; -7.25]
Cork-based Living Wall {Cortes 2022}	24.76	8.2700	26	23.75	6.0200	26	2.3%	1.01	[-2.92; 4.94]
Continuous Living Wall {Cruciol-Barbosa 2023}	30.12	3.1100	56	32.50	2.9000	56	3.3%	-2.38	[-3.49; -1.27]
Felt layer {Nori 2013}	20.56	2.4000	24	25.60	10.9500	24	2.1%	-5.04	[-9.52; -0.56]
**Total (95% CI)**			**764**			**764**	**55.2%**	**-3.88**	**[-5.78; -1.97]**
Heterogeneity: $Tau^2 = 14.1723$; $Chi^2 = 624.29$, df = 22 ($P < 0.01$); $I^2 = 96\%$									
**Total (95% CI)**			**13696**			**13696**	**100.0%**	**-3.22**	**[-4.37; -2.07]**

Heterogeneity: $Tau^2 = 8.8224$; $Chi^2 = 988.72$, df = 39 ($P < 0.01$); $I^2 = 96\%$
Test for subgroup differences: $Chi^2 = 2.93$, df = 1 ($P = 0.09$)

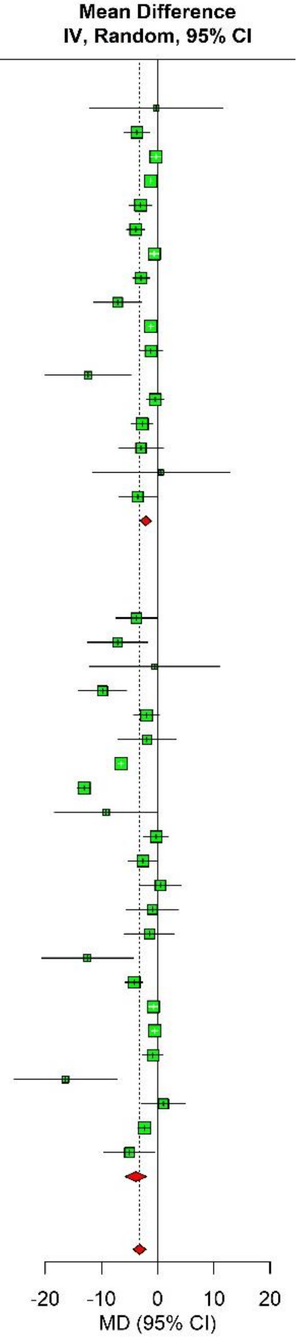

Modified Concrete vs Conventional Concrete – Surface Temperature

Interventions	Interventions Mean	SD	N	Control Mean	SD	N	Weight	MD	95% CI
**Modified Concrete vs Concrete**									
Concrete paver (PC) on concrete {Barišic´ 2022}	33.44	11.5401	18	30.61	9.1419	18	3.4%	2.83	[-3.97; 9.63]
Concrete paver (PC) on grass {Barišic´ 2022}	33.38	11.8764	20	30.20	9.1643	20	3.5%	3.18	[-3.40; 9.75]
Pervious/Porous concrete (P) on concrete {Barišic´ 2022}	33.22	10.9507	18	30.61	9.1419	18	3.5%	2.61	[-3.98; 9.20]
Pervious/Porous concrete (P) on grass {Barišic´ 2022}	33.55	11.1590	20	30.20	9.1643	20	3.6%	3.35	[-2.98; 9.68]
Pervious/Porous concrete (P)1 {Kevern 2012}	37.90	6.1290	24	35.50	4.7388	24	4.8%	2.40	[-0.70; 5.50]
Pervious/Porous concrete (P)2 {Takebayashi 2012}	35.22	8.3745	9	34.47	6.5715	9	3.3%	0.76	[-6.20; 7.71]
Water-retaining concrete, A {Takebayashi 2012}	34.61	8.7385	9	34.47	6.5715	9	3.3%	0.14	[-7.00; 7.29]
Water-retaining concrete, B {Takebayashi 2012}	35.78	8.8534	9	34.47	6.5715	9	3.2%	1.31	[-5.89; 8.51]
Interlocking block-porous {Takebayashi 2012}	33.61	7.5984	11	34.47	6.5715	11	3.7%	-0.86	[-6.79; 5.08]
Interlocking block water retaining {Takebayashi 2012}	34.17	7.4498	11	34.47	6.5715	11	3.7%	-0.30	[-6.17; 5.57]
Permeable concrete {Wang 2018}	33.00	9.7047	12	30.83	7.8721	12	3.3%	2.17	[-4.90; 9.24]
**Total (95% CI)**			**161**			**161**	**39.3%**	**1.74**	**[0.81; 2.68]**
Heterogeneity: $Tau^2 = 0$; $Chi^2 = 2.26$, df = 10 (P = 0.99); $I^2 = 0\%$									
**Bricks/Tiles/Pavers vs Concrete**									
Porous pavement 10% (PA1) {Ferrari 2020}	27.78	10.7823	9	27.94	10.1194	9	2.4%	-0.17	[-9.83; 9.49]
Porous pavement 20% (PA2) {Ferrari 2020}	27.67	11.4182	9	27.94	10.1194	9	2.4%	-0.28	[-10.25; 9.69]
Porous pavement 30% (PA3) {Ferrari 2020}	27.44	12.0687	9	27.94	10.1194	9	2.3%	-0.50	[-10.79; 9.79]
PA1-PA2 {Ferrari 2020}	26.06	10.1318	9	26.31	11.3639	9	2.4%	-0.26	[-10.20; 9.69]
PA1-PA3 {Ferrari 2020}	23.89	9.6946	9	26.31	11.3639	9	2.4%	-2.42	[-12.18; 7.34]
PA2-PA1 {Ferrari 2020}	26.31	11.3639	9	26.31	11.3639	9	2.2%	0.00	[-10.50; 10.50]
Cool pavement (Lime cement plaster) {Kolokotsa 2018}	33.49	2.8997	12	36.05	3.5482	12	5.0%	-2.56	[-5.15; 0.04]
Ceramic porous brick {Wang 2018}	29.33	9.1486	12	28.08	7.2796	12	3.5%	1.25	[-5.36; 7.86]
**Total (95% CI)**			**78**			**78**	**22.6%**	**-1.68**	**[-2.88; -0.48]**
Heterogeneity: $Tau^2 = 0$; $Chi^2 = 1.61$, df = 7 (P = 0.98); $I^2 = 0\%$									
**Coatings vs Concrete**									
Solar-reflective Cool Pavement1 {Middel 2020}	43.74	6.0457	9	43.71	5.8736	9	3.9%	0.03	[-5.47; 5.54]
Solar-reflective Cool Pavement2 {Middel 2020}	45.66	6.5787	7	44.56	6.2016	7	3.4%	1.10	[-5.60; 7.80]
**Total (95% CI)**			**16**			**16**	**7.3%**	**0.46**	**[-6.19; 7.11]**
Heterogeneity: $Tau^2 = 0$; $Chi^2 = 0.06$, df = 1 (P = 0.81); $I^2 = 0\%$									
**Metals vs Concrete**									
Conventional aluminum tile {Elqattan 2021}	43.11	1.0425	14	41.89	0.7931	14	5.4%	1.22	[0.54; 1.91]
Cool aluminum tile {Elqattan 2021}	29.63	0.2920	14	41.89	0.7931	14	5.4%	-12.26	[-12.70; -11.81]
Cool aluminum box {Elqattan 2021}	27.91	0.3302	14	41.89	0.7931	14	5.4%	-13.97	[-14.42; -13.52]
Iron {Liang 2019}	30.28	9.0799	9	30.68	9.6687	9	2.7%	-0.40	[-9.07; 8.27]
**Total (95% CI)**			**51**			**51**	**19.0%**	**-6.74**	**[-19.34; 5.85]**
Heterogeneity: $Tau^2 = 60.1892$; $Chi^2 = 1414.96$, df = 3 (P < 0.01); $I^2 = 100\%$									
**Miscellaneous vs Concrete**									
Cystosepiment {Liang 2019}	30.83	14.0178	9	30.68	9.6687	9	2.1%	0.16	[-10.97; 11.28]
**Natural Materials vs Concrete**									
Wood {Liang 2019}	30.33	11.6539	9	30.68	9.6687	9	2.4%	-0.34	[-10.24; 9.55]
Grass {Takebayashi 2012}	29.67	3.9686	9	34.47	6.5715	9	4.1%	-4.80	[-9.82; 0.22]
Soil {Takebayashi 2012}	32.28	8.2957	9	34.47	6.5715	9	3.3%	-2.19	[-9.10; 4.73]
**Total (95% CI)**			**27**			**27**	**9.8%**	**-3.39**	**[-8.55; 1.77]**
Heterogeneity: $Tau^2 = 0$; $Chi^2 = 0.78$, df = 2 (P = 0.68); $I^2 = 0\%$									
**Total (95% CI)**			**342**			**342**	**100.0%**	**-1.14**	**[-2.91; 0.63]**
Heterogeneity: $Tau^2 = 20.0097$; $Chi^2 = 1712.61$, df = 28 (P = 0); $I^2 = 98\%$									
Test for subgroup differences: $Chi^2 = 39.40$, df = 5 (P < 0.01)									

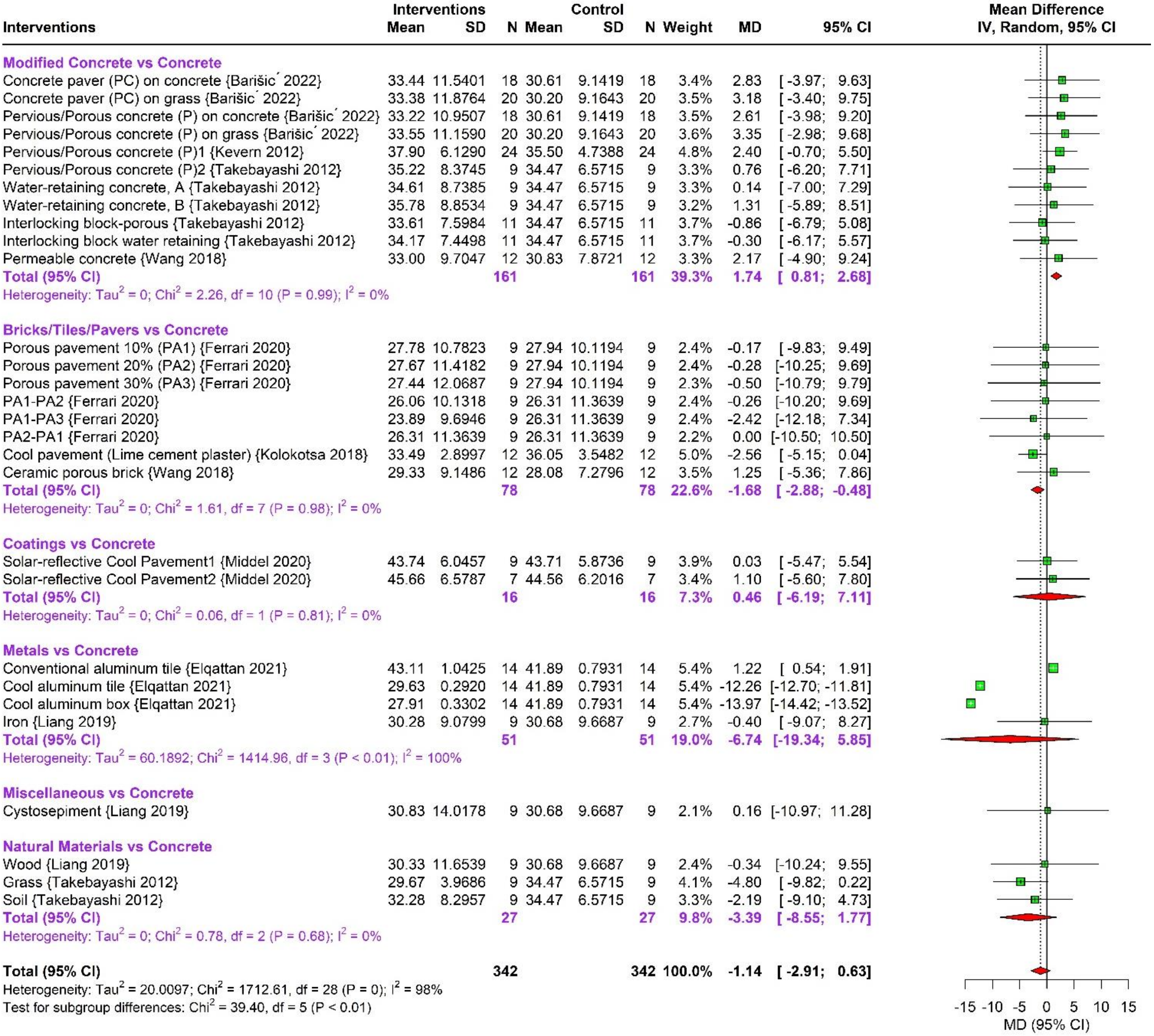

Modified Concrete vs Conventional Concrete – Surface Temperature (without Elqattan 2021)

	Interventions			Control					
Interventions	Mean	SD	N	Mean	SD	N	Weight	MD	95% CI
**Modified Concrete vs Concrete**									
Concrete paver (PC) on concrete {Barišic´ 2022}	33.44	11.5401	18	30.61	9.1419	18	2.4%	2.83	[-3.97; 9.63]
Concrete paver (PC) on grass {Barišic´ 2022}	33.38	11.8764	20	30.20	9.1643	20	2.6%	3.18	[-3.40; 9.75]
Pervious/Porous concrete (P) on concrete {Barišic´ 2022}	33.22	10.9507	18	30.61	9.1419	18	2.6%	2.61	[-3.98; 9.20]
Pervious/Porous concrete (P) on grass {Barišic´ 2022}	33.55	11.1590	20	30.20	9.1643	20	2.8%	3.35	[-2.98; 9.68]
Pervious/Porous concrete (P)1 {Kevern 2012}	37.90	6.1290	24	35.50	4.7388	24	9.0%	2.40	[-0.70; 5.50]
Pervious/Porous concrete (P)2 {Takebayashi 2012}	35.22	8.3745	9	34.47	6.5715	9	2.3%	0.76	[-6.20; 7.71]
Water-retaining concrete, A {Takebayashi 2012}	34.61	8.7385	9	34.47	6.5715	9	2.2%	0.14	[-7.00; 7.29]
Water-retaining concrete, B {Takebayashi 2012}	35.78	8.8534	9	34.47	6.5715	9	2.2%	1.31	[-5.89; 8.51]
Interlocking block-porous {Takebayashi 2012}	33.61	7.5984	11	34.47	6.5715	11	3.1%	-0.86	[-6.79; 5.08]
Interlocking block water retaining {Takebayashi 2012}	34.17	7.4498	11	34.47	6.5715	11	3.2%	-0.30	[-6.17; 5.57]
Permeable concrete {Wang 2018}	33.00	9.7047	12	30.83	7.8721	12	2.2%	2.17	[-4.90; 9.24]
**Total (95% CI)**			**161**			**161**	**34.4%**	**1.74**	**[0.81; 2.68]**
Heterogeneity: $Tau^2 = 0$; $Chi^2 = 2.26$, df = 10 (P = 0.99); $I^2 = 0\%$									
**Bricks/Tiles/Pavers vs Concrete**									
Porous pavement 10% (PA1) {Ferrari 2020}	27.78	10.7823	9	27.94	10.1194	9	1.2%	-0.17	[-9.83; 9.49]
Porous pavement 20% (PA2) {Ferrari 2020}	27.67	11.4182	9	27.94	10.1194	9	1.2%	-0.28	[-10.25; 9.69]
Porous pavement 30% (PA3) {Ferrari 2020}	27.44	12.0687	9	27.94	10.1194	9	1.1%	-0.50	[-10.79; 9.79]
PA1-PA2 {Ferrari 2020}	26.06	10.1318	9	26.31	11.3639	9	1.2%	-0.26	[-10.20; 9.69]
PA1-PA3 {Ferrari 2020}	23.89	9.6946	9	26.31	11.3639	9	1.2%	-2.42	[-12.18; 7.34]
PA2-PA1 {Ferrari 2020}	26.31	11.3639	9	26.31	11.3639	9	1.1%	0.00	[-10.50; 10.50]
Cool pavement (Lime cement plaster) {Kolokotsa 2018}	33.49	2.8997	12	36.05	3.5482	12	11.5%	-2.56	[-5.15; 0.04]
Ceramic porous brick {Wang 2018}	29.33	9.1486	12	28.08	7.2796	12	2.5%	1.25	[-5.36; 7.86]
**Total (95% CI)**			**78**			**78**	**21.0%**	**-1.68**	**[-2.88; -0.48]**
Heterogeneity: $Tau^2 = 0$; $Chi^2 = 1.61$, df = 7 (P = 0.98); $I^2 = 0\%$									
**Coatings vs Concrete**									
Solar-reflective Cool Pavement1 {Middel 2020}	43.74	6.0457	9	43.71	5.8736	9	3.5%	0.03	[-5.47; 5.54]
Solar-reflective Cool Pavement2 {Middel 2020}	45.66	6.5787	7	44.56	6.2016	7	2.5%	1.10	[-5.60; 7.80]
**Total (95% CI)**			**16**			**16**	**6.0%**	**0.46**	**[-6.19; 7.11]**
Heterogeneity: $Tau^2 = 0$; $Chi^2 = 0.06$, df = 1 (P = 0.81); $I^2 = 0\%$									
**Metals vs Concrete**									
Conventional aluminum tile {Elqattan 2021}	43.11	1.0425	14	41.89	0.7931	14	28.4%	1.22	[0.54; 1.91]
Iron {Liang 2019}	30.28	9.0799	9	30.68	9.6687	9	1.5%	-0.40	[-9.07; 8.27]
**Total (95% CI)**			**23**			**23**	**29.9%**	**1.21**	**[-0.41; 2.83]**
Heterogeneity: $Tau^2 = 0$; $Chi^2 = 0.13$, df = 1 (P = 0.71); $I^2 = 0\%$									
**Miscellaneous vs Concrete**									
Cystosepiment {Liang 2019}	30.83	14.0178	9	30.68	9.6687	9	0.9%	0.16	[-10.97; 11.28]
**Natural Materials vs Concrete**									
Wood {Liang 2019}	30.33	11.6539	9	30.68	9.6687	9	1.2%	-0.34	[-10.24; 9.55]
Grass {Takebayashi 2012}	29.67	3.9686	9	34.47	6.5715	9	4.2%	-4.80	[-9.82; 0.22]
Soil {Takebayashi 2012}	32.28	8.2957	9	34.47	6.5715	9	2.3%	-2.19	[-9.10; 4.73]
**Total (95% CI)**			**27**			**27**	**7.7%**	**-3.39**	**[-8.55; 1.77]**
Heterogeneity: $Tau^2 = 0$; $Chi^2 = 0.78$, df = 2 (P = 0.68); $I^2 = 0\%$									
**Total (95% CI)**			**314**			**314**	**100.0%**	**0.40**	**[-0.40; 1.19]**
Heterogeneity: $Tau^2 = 0.9826$; $Chi^2 = 17.54$, df = 26 (P = 0.89); $I^2 = 0\%$									
Test for subgroup differences: $Chi^2 = 48.02$, df = 5 (P < 0.01)									

Mean Difference
IV, Random, 95% CI

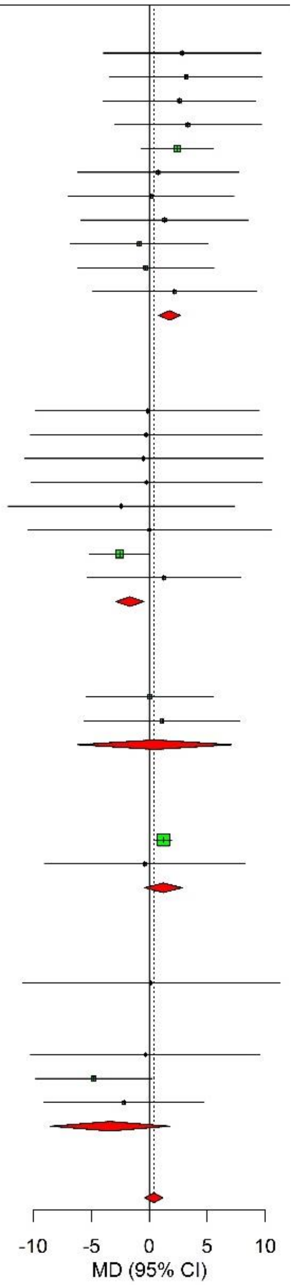

Modifications in Building Roofs vs Conventional Roof – Indoor Temperature

Interventions	Interventions Mean	SD	N	Control Mean	SD	N	Weight	MD	95% CI
**Reflective Roof vs Conventional Roof**									
Solar reflective White Paint {Vellingiri 2020}	33.50	1.1200	15	31.60	0.2100	15	11.5%	1.90	[1.32; 2.48]
High Reflective Paint Roof {Seo 2023}	32.43	1.0965	7	35.79	1.5774	7	9.9%	-3.36	[-4.78; -1.93]
Reflective Cooling {Lei 2024}	39.07	4.3684	17	39.33	4.8398	17	6.0%	-0.27	[-3.37; 2.83]
Exterior Weather Shield Ultra Clean Coating {Rawat 2022}	31.35	3.1277	24	34.00	3.8258	24	8.5%	-2.65	[-4.63; -0.68]
Exterior TR E2000 {Rawat 2022}	31.48	3.6540	24	34.00	3.8258	24	8.1%	-2.52	[-4.64; -0.41]
Exterior Weather Shield E1000 {Rawat 2022}	32.61	3.3404	24	34.00	3.8258	24	8.3%	-1.39	[-3.42; 0.64]
Exterior Weather Shield Flex {Rawat 2022}	31.57	3.5577	24	34.00	3.8258	24	8.2%	-2.43	[-4.52; -0.34]
Ceramic coating {Garg 2016}	20.00	32.5250	13	33.38	1.7612	13	0.4%	-13.38	[-31.08; 4.33]
Reflective White Paint {Akbari 2003}	25.93	6.2075	27	29.42	9.9967	27	3.9%	-3.50	[-7.94; 0.94]
**Total (95% CI)**			**175**			**175**	**64.8%**	**-1.69**	**[-3.35; -0.02]**
Heterogeneity: $Tau^2 = 3.3312$; $Chi^2 = 84.7$, df = 8 ($P < 0.01$); $I^2 = 91\%$									
**Recycled materials vs Conventional Roof**									
Modroof {Vellingiri 2020}	33.80	1.0300	15	33.75	0.7600	15	11.4%	0.05	[-0.60; 0.70]
**Plastic vs Conventional Roof**									
Thermocol Sheet {Vellingiri 2020}	33.50	0.7300	15	33.25	1.0900	15	11.4%	0.25	[-0.41; 0.91]
**Misc vs Conventional Roof**									
Radiative cooling {Lei 2024}	38.80	3.9655	17	39.33	4.8398	17	6.2%	-0.54	[-3.51; 2.44]
Aerated concrete {Lei 2024}	39.47	4.0054	17	39.33	4.8398	17	6.2%	0.13	[-2.85; 3.12]
**Total (95% CI)**			**34**			**34**	**12.4%**	**-0.20**	**[-4.46; 4.05]**
Heterogeneity: $Tau^2 = 0$; $Chi^2 = 0.1$, df = 1 ($P = 0.76$); $I^2 = 0\%$									
**Total (95% CI)**			**239**			**239**	**100.0%**	**-1.05**	**[-2.20; 0.10]**
Heterogeneity: $Tau^2 = 2.4948$; $Chi^2 = 85.57$, df = 12 ($P < 0.01$); $I^2 = 86\%$									
Test for subgroup differences: $Chi^2 = 6.17$, df = 3 ($P = 0.10$)									

Mean Difference IV, Random, 95% CI

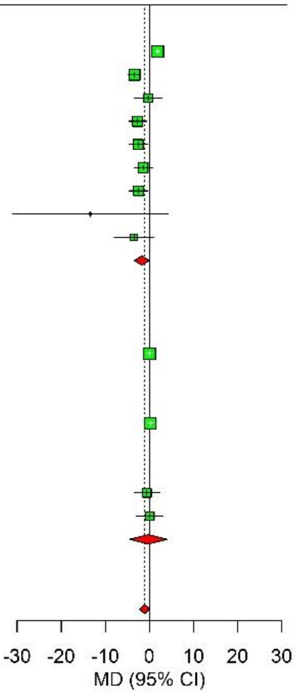

Modifications in Building Roofs vs Conventional Roof – Indoor Temperature (without Garg 2016)

Interventions	Interventions Mean	SD	N	Control Mean	SD	N	Weight	MD	95% CI
**Reflective Roof vs Conventional Roof**									
Solar reflective White Paint {Vellingiri 2020}	33.50	1.1200	15	31.60	0.2100	15	11.6%	1.90	[1.32; 2.48]
High Reflective Paint Roof {Seo 2023}	32.43	1.0965	7	35.79	1.5774	7	9.9%	-3.36	[-4.78; -1.93]
Reflective Cooling {Lei 2024}	39.07	4.3684	17	39.33	4.8398	17	6.0%	-0.27	[-3.37; 2.83]
Exterior Weather Shield Ultra Clean Coating {Rawat 2022}	31.35	3.1277	24	34.00	3.8258	24	8.5%	-2.65	[-4.63; -0.68]
Exterior TR E2000 {Rawat 2022}	31.48	3.6540	24	34.00	3.8258	24	8.2%	-2.52	[-4.64; -0.41]
Exterior Weather Shield E1000 {Rawat 2022}	32.61	3.3404	24	34.00	3.8258	24	8.4%	-1.39	[-3.42; 0.64]
Exterior Weather Shield Flex {Rawat 2022}	31.57	3.5577	24	34.00	3.8258	24	8.2%	-2.43	[-4.52; -0.34]
Reflective White Paint {Akbari 2003}	25.93	6.2075	27	29.42	9.9967	27	3.9%	-3.50	[-7.94; 0.94]
**Total (95% CI)**			**162**			**162**	**64.6%**	**-1.61**	**[-3.22; 0.01]**
Heterogeneity: $Tau^2 = 3.2923$; $Chi^2 = 82.39$, df = 7 ($P < 0.01$); $I^2 = 92\%$									
**Recycled materials vs Conventional Roof**									
Modroof {Vellingiri 2020}	33.80	1.0300	15	33.75	0.7600	15	11.5%	0.05	[-0.60; 0.70]
**Plastic vs Conventional Roof**									
Thermocol Sheet {Vellingiri 2020}	33.50	0.7300	15	33.25	1.0900	15	11.5%	0.25	[-0.41; 0.91]
**Misc vs Conventional Roof**									
Radiative cooling {Lei 2024}	38.80	3.9655	17	39.33	4.8398	17	6.2%	-0.54	[-3.51; 2.44]
Aerated concrete {Lei 2024}	39.47	4.0054	17	39.33	4.8398	17	6.2%	0.13	[-2.85; 3.12]
**Total (95% CI)**			**34**			**34**	**12.4%**	**-0.20**	**[-4.46; 4.05]**
Heterogeneity: $Tau^2 = 0$; $Chi^2 = 0.1$, df = 1 ($P = 0.76$); $I^2 = 0\%$									
**Total (95% CI)**			**226**			**226**	**100.0%**	**-1.00**	**[-2.11; 0.11]**
Heterogeneity: $Tau^2 = 2.4714$; $Chi^2 = 83.30$, df = 11 ($P < 0.01$); $I^2 = 87\%$									
Test for subgroup differences: $Chi^2 = 6.20$, df = 3 ($P = 0.10$)									

Mean Difference IV, Random, 95% CI

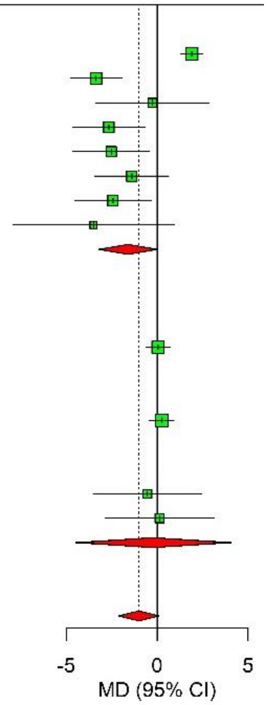

Supplement: online supplemental file 8 [file bmjph-3-2-s008.pdf]
