# Supplementary material for: Evaluation of community-based heat adaptation interventions: a systematic review
Source: BMJ Public Health. 2025 Jul 15;3(2):e002332. doi: 10.1136/bmjph-2024-002332 (PMC12273142; doi:10.1136/bmjph-2024-002332)
Supplement: online supplemental file 9 [file bmjph-3-2-s009.docx]

| **Supplementary Table 1: Summary of findings for the effect of individual-level adaptation interventions** | | | | | | | | |
| --- | --- | --- | --- | --- | --- | --- | --- | --- |
| Author, year | Study design/  setting | Country | | Intervention | | Population characteristics /Mortality data | Outcomes Assessed | |
| **Heat Action Plan (HAP)** | | | | | | | | |
| Chau 2009 ^157^ | Pre-post  (city-wide) | Hong Kong | | National HAP-2000  (Very Hot Weather Warning System, heat shelters, water, hotline) | | Census & Statistics Department  (1997-1999 vs 2000-2005) | IHD: 1.23 increase in deaths per day among elderly (95% CI: 0.32, 2.14) (control) Stroke: 0.97 increase in deaths per day among elderly (95% CI: 0.02, 1.92) (control) | |
| Foiuillet 2008 ^198^ | Pre-post  (country-wide) | France | | National-2003  (National Heat Wave Plan)  Data surveillance, emergency plans and air-conditioning equipment for hospitals and old homes, warning system | | French National Institute for Medical Research (1975-1999 vs 2000-2003, 2004-2006) | Excess mortality deficit: -4388 deaths (95%CI: -4920 to -3855) | |
| de'Donato 2015 ^141^ | Pre-post  (multiple cities) | UK, France, Finland, Italy, Hungary, Spain Sweden, Greece | | National – 2004  (Early warning systems, health  surveillance, information campaigns, targeted prevention measures to at-risk groups.) | | Mortality data  (1996-2002 vs 2004-2010) | Attributable deaths: 623-985 less deaths in three cities | |
| de'Donato 2018 ^140^ | Pre-post  (multiple cities) | Italy | | National-2004  (Heat Health Watch Warning System, helpline, informative campaigns, healthcare staff trainings, emergency protocols, cooling interventions) | | Municipal registry mortality data (1999-2002 vs 2005-2008, 2009-2012, 2013-2016) | Attributable deaths: 1261-2780 less deaths | |
| Hess 2018 ^143^ | Pre-post  (city-wide) | India | | City-wide-2014  (community outreach, early warning system, healthcare capacity building) | | Municipal Corporation (2007-2010 vs 2014-2015) | Post-to-pre-HAP nonlagged mortality IRR: Tmax over 40°C: 0.95 ((0.73–1.22)  Tmax over 45°C: 0.73 (0.29–1.81) Mortality reduced: mean 1,190 (95%CI 162–2,218)/year | |
| Martínez-Solanas 2019 ^146^ | Pre-post  (country-wide) | Spain | | National-2004  (Heat Health Prevention Plan)  Weather forecasts, information dissemination, hotline and emergency services. | | Spanish National Statistics Institute (1993–2002 vs 2004-2013) | Attributable deaths:  Pre: 0.67%,  Post: 0.56% | |
| Morabito 2011 ^160^ | Pre-post  (multiple municipalities) | Italy | | National-2003  (Active Surveillance of the Frail Elderly) | | Mortality Registry of the Tuscany Region  (1997-2002 vs 2004-2007) | Heat mortality Lack of HHWS (1999–2002): OR 1.23 Experimental HHWS running only for Florence (2004–2005): OR 1.21 official HHWS extended to the whole Florentine area (2006–2007): OR 1.12 | |
| Nitschke 2016 ^161^ | Ecological  (city-wide) | Australia | | National-2009 (Hazard Leader: Heat advice, collaborative heat plan, review of emergency services, hotlines) | | Department for Health and Ageing (2009 vs 2014) | Estimated excess deaths: 2009: 34.5 2014: 38.2 | |
| Schifano 2012 ^148^ | Pre-post (multiple cities) | Italy | | National-2004  (Heat Health Watch Warning System: monitoring of high-risk patients) | | Elderly 65+ years old Local mortality registries, Rapid mortality surveillance system (1998-2002 vs 2006-2010) | Mortality risk: dropped from 37% to 13% between 9-12°C | |
| Benmarhnia 2016 ^139^ | Quasi-experimental (DID) (city-wide) | Canada | | National-2004  (Public advisory, surveillance, healthcare capacity building, public pools, air-conditioned shelters) | | Montreal death registry data (2000-2003 vs 2004-2007) | Mortality:4.87 deaths/day (95% CI: – 5.38,0.34) for hot days | |
| Heo 2019 ^142^ | Quasi-experimental (DID) (multiple cities) | South Korea | | National-2008  (Monitoring of high-risk patients, shaded shelters, occupational heat education, regulate working hours) | | Korea Center for Disease Control & Prevention (2009-2014) | Cardiovascular mortality  For aged 75+ and unemployed: -5.797 deaths/1,000,000 people/day (95% CI: -10.856, -0.739) For aged 75+ and widowed: -4.524 deaths/1,000,000 population/day (95% CI: -8.617, -0.431]) For aged 19–64 and no education: -0.091 deaths/1,000,000 population/day (95% CI: 0.140, 0.042) For aged 65+ with university education or higher degrees: -0.612 deaths/1,000,000 population (95% CI: -1.140, -0.084) For aged 75+ with university education or higher degrees: -1.688 deaths/1,000,000 population (95% CI: -3.050, -0.325)   Respiratory mortality For aged 0-19: -0.09 deaths/1,000,000 population/day (95% CI: -0.128, -0.053) For aged 65+ and widowed: -0.31 deaths/1,000,000 population/day (95% CI: -0.526, -0.094) For aged 65+ and single:-0.349 deaths/1,000,000 population/day (95% CI: -0.534, -0.163) For aged 19–64 and no education: -0.032 deaths/1,000,000 population/day (95% CI: -0.055, -0.009) For aged 65+ with elementary education: -0.884 deaths/1,000,000 population/day (95% CI: -1.582, -0.185) For aged 75+ with university education or higher degrees: -0.999 deaths/1,000,000 population/day (95% CI: -1.947, -0.050) | |
| Jing 2016 ^144^ | Quasi-experimental (multiple neighborhoods) | China | | Community trial  (Heat Wave Intervention Program: Heat early warning & preparation, 24hrs consulting services, heat-related training) | | Surveys  2014 (N=2240),  2015 (N=2356) | Health-related illness: OR=0.769 (overall), 0.495 (rural), 1.281 (urban)  Cost-effectiveness ratio: Intervention: $15.06 Control: $15.69 | |
| Xu 2018^149^ | Quasi-experimental (multiple neighborhoods) | China | | Community trial  (Heat Wave Intervention Program: Heat early warning & preparation, 24hrs consulting services, heat-related training) | | Surveys 2014, 2015  (N=2400) | Knowledge: ß = 0.387, P < 0.001 Attitude: ß = 0.166, P < 0.01 | |
| Mattern 2000 ^153^ | Pre-post | USA | | Community trial  (Bridging the Gap: heat-health education and about heat-related resource, and obtain information about their perceptions of and effective barriers to heat-related morbidity and the associated medical conditions) | | Elderly 65+ years old (N=34) | Pretest: Contact for assistance: 67%  Heat hotline: 3%  Posttest:  Contact for assistance: 94% Heat hotline: 29% Use of thermometer: 97% | |
| Liotta 2018 ^145^ | Quasi-experimental retrospective cohort (multiple districts) | Italy | | Community trial  (Long Live Elderly: health promotion campaign, periodic checkups, awareness, health services) | | Elderly 75+ years old  (cases: n=6483, control: n=5724) | Cumulative mortality rate: Intervention group: 25 ±1.4h; Cl 95%: 23–29 Control group: 29 ±6.7h; Cl 95%: 17–43. | |
| Orlando 2021 ^145^ | Quasi-experimental retrospective cohort (multiple districts) | Italy | | Community trial  (Long Live Elderly: health promotion campaign, periodic checkups, awareness, health services)) | | Elderly 75+ years old (cases: n=6483, control: n=5724) | The EMR in the intervention and controls was 2.70% and 3.81%, respectively. The rate ratio was 0.70 (95% CI: 0.54–0.92, p-value 0.01). IRR of the interventions, with respect to the controls, was 0.76 (95% CI: 0.59–0.98). After adjusting for other variables, the IRR was 0.44 (95% CI: 0.32–0.60). | |
| **Heat Warning System** | | | | | | | | |
| Mehiriz 2018^159^ | RCT | Canada | | Community trial (automated phone heat warnings) | | Intervention: n=662, Control: n=666 | Increased knowledge and behaviors in intervention group | |
| Weinberger 2018 ^162^ | Pre-post  (multiple cities) | USA | | National (NWS heat alerts) | | US National Center for Health Statistics  (2001-2006) | Overall percent change in mortality rate: −0.5% (95% CI: -2.8, 1.9) Philadelphia:  Percent change in mortality rate: 4.4% (95% CI: -8.3, −0.3)  Death averted per year: 45.1 (95% CI: 3.1, 84.1) | |
| Weinberger 2021 ^163^ | Pre-post  (multiple counties) | USA | | National (NWS heat alerts) | | Medicare beneficiaries aged 65+ years.  US National Center for Health Statistics (2006-2016) | Mortality risk: RR: 1.005 (95% CI: 0.997, 1.013) | |
| **Heat Education** | | | | | | | | |
| Joubert 2011 ^151^ | Pre-post | United Arab Emirates | | IEC material dissemination | | Companies’ employees  (N=465 companies) | Heat related illness:  79.5% decrease in in heat-related treatment and emergency cases 50% reduction in serious cases | |
| Das 2016 ^150^ | Pre-post  (state-wide) | India | | State-wide media campaign | | Office of Special Relief Commissioner, Govt. of Odisha  (2005-2006 vs 2007-2012) | Repeated use of video media to be the most effective in reducing deaths followed by newspapers and radio. | |
| Khan 2023 ^178^ | Quasi-experimental (Pre-post) | Pakistan | | Heat  Emergency Awareness and Treatment | | Patients with potential heat emergency symptoms  Pre (4181), Post (4022) | Diagnosis rate:  Pre 3% (*n* = 125/4181), Post 7.5% (*n* = 7.5/4022) (*p* < 0.001)  Temperature monitoring:  Pre 0.9% (*n* = 41/4181), Post 13% (*n* = 496/4022) (*p* < 0.001) | |
| Nitschke 2017 ^154^ | RCT | Australia | | Community trial (Heat Health Messages study) | | Elderly 65+ years old (N=637) | Knowledge increase: 94% (intervention), 88% (control) Self-reported heat stress: RR 0.37; 95% CI: 0.22–0.63 | |
| Li 2022b ^177^ | RCT | China | | School heat health education trial | | 3rd-5th class students (Intervention: n=405,  control school: n=539) | KAP Score change:  Students: 19.9% (95%CI: 16.3%, 23.6%), 9.60% (95%CI: 5.35%, 13.9%), 9.94% (95%CI: 8.26%, 18.3%) Parents: 22.5% (95%CI: 17.8%, 27.1%), 7.22% (95%CI: 0.96%, 13.5%), 5.22% (95%CI: 0.73%, 9.71%)  Boys’ slightly higher than girls’ Older students’ higher than younger students’  Higher educated parents’ higher Female parents’ higher than in male parents’ | |
| Razzak 2022 ^155^ | Quasi-experimental (multiple neighborhoods) | Pakistan | | CHW-led community trial (Heat Emergency Awareness and Treatment Trial) | | Intervention: n=8668,  Control: n=9877 | Hospital visits: RR 0.62; 95% CI: 0.49–0.77 | |
| Santos 2022 ^156^ | RCT | USA | | Heat Education and Awareness Tool | | Agricultural workers (cane cutters) (Intervention: n=37, Control: n=38) | Physiological strain index (PSImax): 1.96 (t = 3.81, p<0.001) at high versus low/medium-low effort | |
| Sorensen 2023 ^179^ | Pre-post | Caribbean region (37 countries) | | Climate and health capacity building course | | Health professionals (n=132) | Significant changes in health professional communication, engagement and application of climate and health knowledge and skills | |
| **Modification in Clothing** | | | | | | | | |
| Li 1996a ^170^ | Two groups | Japan | | Trousers, skirts | | Female employees (N=10) | Rectal temperature (Range of oscillation): Skirt group: 0.67+0.07°C (March), 0.80+0.08°C (July) Trousers group: 0.69+0.05°C (March), 0.59+0.08°C (July)  Min Core temperature: Skirt group: 36.70+0.15°C (March), 36.40+0.13°C (July) Trousers group: 36.48+0.09°C (March), 36.51+0.08°C (July)  Leg skin temperature increase (evening to night): Skirt group: 1.78+0.22°C (March), 2.93+0.17°C (July) Trousers group: 1.33+0.30°C (March), 2.20+0.13°C (July) | |
| Kuwabara 2020 ^169^ | Case-crossover | Japan | | Ventilated garments | | Male construction workers  (N=10) | The overall, chest, and forearm skin temperatures of the workers not wearing VWW were significantly higher than those of workers wearing VWW. | |
| Chan 2016a ^165^ | Experiment/Pilot/Post only | Hong Kong | | Cooling vest | | Various industry workers (N=169) | Physical strain alleviation (PSA: 21.1% Construction: 14.8% Horticulture and cleaning: 18.8% Kitchen and catering: 27.4% Airport apron service: 26.5% | |
| Chan 2016b ^168^ | Case-crossover | Hong Kong | | Light/loose garment | | Construction workers (N=184) | New uniform:  Preference: 36% higher  Work performance: 35% higher | |
| Chan 2017 ^166^ | Case-crossover | Hong Kong | | Cooling vest | | Male construction workers  (N=140) | Reduced heart rate thermal sensation, perceived exertion, heat strain  Higher rating on preference, and perceived fitness, heat stress alleviation, sensations of comfort, skin dryness and cooling effect Perceptual heat stain alleviation: twice that of traditional Preference=91% | |
| Dehghan 2023 ^180^ | Two groups | Iran | | Evaporative cooling vest | | Male construction workers  (N=60) | Heart rate: Users 111.1 ± 7.6 bpm, Non-users 114.3 ± 7.9 (P ˂ 0.05)  Oral temperature: Users 36.36 ± 0.41, Non-users 36.37 ± 0.55  Physiological Strain Index: Users 2.53 ± 0.66, Non-users 3.64 ± 0.81 (P ˂ 0.05)  PeSI: Users 4.64 ± 0.79, Non-users 6.41 ± 0.85 (P ˂ 0.05)  HSSI: Users 10.12 ± 1.71, Non-users 15.88 ± 0.1.43 (P ˂ 0.05) | |
| Yang 2017 ^18^ | RCT | Hong Kong | | Light/loose garment | | Construction workers (N16) | Perpetual strain alleviated and comfort increased significantly | |
| Zhao 2018 ^167^ | Case-crossover | Hong Kong | | Cooling vest | | Male construction workers (N=14) | Physiological and perceptual strain and heat strain were significantly reduced. | |
| Ashtekar 2019 ^164^ | Experiment | India | | Cooling vest | | Male construction workers  (N=29) | Mean skin temperature reduction:  PCG: 38.66 ± 33.98°F Normal clothing: 32.36 ± 33.44°F (p < .05).  Mean sweat loss PCG: 0.365 ± 0.257 kg Normal clothing: 0.658 ± 0.342 kg (p < .05)  Heart rate, along with back and chest skin temperatures were significantly reduced with wearing PCG. | |
| Chicas 2021 ^17^ | RCT | USA | | Cooling vest, cooling bandana | | Agricultural workers (N=78) | Core body temperature exceeding 38°C: Bandana: 38% OR=0.7 (90% CI=0.2,3.2) Vest: 60% OR=1.8 (90% CI=0.4,7.9) Combination: 53% OR=1.3 (90% CI=0.3,5.6) Control: 46%  Heat-related illness Bandana: 32% Vest: 40% Combination: 20% Control: 60% | |
| **Water-Rest-Shade** | | | | | | | | |
| Bodin 2016 ^171^ | Quasi-experimental | El Salvador | | Water.rest.shade | | Agricultural workers  (cane cutters) (N=130) | Self-reported water consumption increased 25%. Heat stress symptoms decreased.  Individual daily production increased from 5.1 to 7.3 tons/person/day. | |
| Wegman 2018 ^172^ | Quasi-experimental | El Salvador | | Water.rest.shade | | Agricultural workers  (cane cutters) (N=117) | eGFR over the 5-month harvest: Intervention: -3.4 mL/min/1.73m2 (95% CI: -5.5, -1.3) Control: -5.3 (95% CI -7.9, -2.7) | |
| Glaser 2020 ^173^ | Pre-post | Nicaragua | | Water.rest.shade | | Agricultural workers  (cane cutters) (N=567) | eGFR: 6 mL/min/1.73 m2 less (95% CI: 2-9 mL/min/1.73 m2)  Incident kidney injury: 70% lower (95% CI: 90%-50%) | |
| Glaser 2022 ^174^ | Pre-post | Nicaragua | | Water.rest.shade | | Agricultural workers  (cane cutters) (N=525) | Almost a three-fourths reduction in kidney injury | |
| **Miscellaneous interventions** | | | | | | | | |
| Takahashi 2015 ^176^ | Community trial | Japan | | 1) heat health warning,  2) water delivery | | Elderly people 65-84 years old  (N=1072) | Heat health warning: Increased hat or parasol use Heat health warning + water: Increased nighttime AC use, water intake, cooling body, reduced activities in heat | |
| Lou 2021 ^199^ | RCT | China | | 1) education,  2) subsidy support,  3) cooling spray | | Elderly people 50+ years old  (N=44) | Education:  Home-cooling practice: 0.76 (95% CI: 0.33–1.19) Learning behaviors: 1.04 (95% CI: 0.65–1.43) Outdoor protection: 0.64 (95% CI: 0.10–1.18)  Subsidy support: Home-cooling practice: 0.55 (95% CI: 0.17–0.93)  Learni ng behaviors: 0.26 (95% CI: -0.08–0.61) Outdoor protection: 0.04 (95% CI: -0.44–0.53)  Cooling-spray:  Home-cooling practice: 0.10 (95% CI: -0.25–0.46)  Learning behaviors: 0.26 (95% CI: -0.07–0.58) Outdoor protection: -0.19 (95% CI: -0.65–0.26) | |
| Huang 2022 ^175^ | RCT | China | | 1) education,  2) subsidy support  3) cooling spray | | Elderly people 50+ years old  (N=44) | Education:  SBP: -8.25mmHg (95%CI: -2.54, -7.78; P = 0.01) HR: −0.91beats/min LSD: −0.07 to 0.06h   Subsidy support: HR: −5.05beats/min LSD: −0.03 to 0.05h   Cooling-spray:  SBP: 3.34mmHg increase (95%CI: 1.76, 4.93; P = 0.04) DSD: -0.21h (95%CI: –0.31, 0.11; P = 0.05) LSD: −0.04 to 0.21h | |
| Maideen 2023 ^200^ | Experimental | Australia | | Fan/Muslin/Flannelette over a stroller | |  | Moist muslin draping plus battery-operated clip-on fan reduces air temperature by 4.7°C and WBGT by 1.4°C.  Dry muslin increases in-stroller temp (*Ta*: +2.6°C; WBGT: +0.9°C)  Flannelette increases in-stroller temp (*Ta*: +3.7°C; WBGT: +1.4°C) | |
| bpm=beats per minute  CHW=Community Health Worker  DSD=Deep Sleep Duration  EMR=Excess Mortality Rate  eGFR= Estimated Glomerular Filtration Rate  HHWS=Heat Health Warning System | | | HR=Heart Rate  HSSI=Heat Strain Score Index  IEC= Information, Education and Communication  IHD=Ischemic Heart Disease  IRR=Incidence Rate Ratios | | KAP=Knowledge, Attitude, Practice  LSD=Light Sleep Duration  NWS=National Weather Service  PCG= Personal Cooling Garment  PeSI=Perceptual Strain Index | | | RCT=Randomized Control Trial  RR=Relative Risk/rate  SBP=Systolic Blood Pressure  Tmax=Maximum Temperature  WBGT=Wet Bulb Globe Temperature |
